# An improved transformer-based concrete crack classification method

**DOI:** 10.1038/s41598-024-54835-x

**Published:** 2024-03-14

**Authors:** Guanting Ye, Wei Dai, Jintai Tao, Jinsheng Qu, Lin Zhu, Qiang Jin

**Affiliations:** 1 College of Hydraulic and Civil Engineering, Xinjiang Agricultural University, Urumqi, 830052 China; 2 College of International Education, Xinjiang Agricultural University, Urumqi, 830052 China

**Keywords:** Image feature extraction, Transformer, Deep learning, Structural health monitoring, Crack detection, Civil engineering, Engineering, Materials science

## Abstract

In concrete structures, surface cracks are an important indicator for assessing the durability and serviceability of the structure. Existing convolutional neural networks for concrete crack identification are inefficient and computationally costly. Therefore, a new Cross Swin transformer-skip (CSW-S) is proposed to classify concrete cracks. The method is optimized by adding residual links to the existing Cross Swin transformer network and then trained and tested using a dataset with 17,000 images. The experimental results show that the improved CSW-S network has an extended range of extracted image features, which improves the accuracy of crack recognition. A detection accuracy of 96.92% is obtained using the trained CSW-S without pretraining. The improved transformer model has higher recognition efficiency and accuracy than the traditional transformer model and the classical CNN model.

## Introduction

Concrete is one of the most commonly used materials in civil engineering, and concrete structures are subjected to external loads, Such as live loads (overloading, vehicle impact, etc.)^1^, natural environments (temperature changes, humidity, acid rain, wind loads and ice loads, etc.)^2^, easily lead to fatigue effects and material degradation. Cracks in concrete structures can affect the local or overall structural safety.

Current crack detection of concrete structures is usually performed by manually capturing crack patterns and analyzing them. This method has disadvantages such as low efficiency, high cost, high risk and unguaranteed detection accuracy^3^. Image processing techniques such as linear array scanning camera^4^, RGB-D sensor^5^, black box camera^6^, accelerometer^7^, laser scanner^8,9^, fuzzy clustering method^10^, X-ray^11^, and fast tomography^12^ have been widely used to detect cracks in concrete structures. However, with the development of vision-based inspection equipment and image processing technology, computer vision has become the main method to detect cracks in concrete structures. In recent years, deep learning^13^ has greatly contributed to the development of computer vision, and methods based on deep learning of convolutional neural networks have been widely used for concrete crack detection. Deep learning based methods include: image recognition, target detection, image segmentation.

The classification-based approach lies in determining whether the image/image patch contains cracks.The method based on convolutional neural network (CNN) was first applied to crack detection^14^. The application of computer vision in crack detection of concrete structures has attracted more and more experts’ attention^15–18^. Li et al.^19^ proposed a unified, purely vision-based approach to detect and classify various typical defects. Gopalakrishnan et al.^20^ proposed different classifiers based on VGG-16 neural network model for automatic classification of hot mix asphalt (HMA) and silicate cement concrete (PCC) images. The single layer trained classifier based on pre-trained VGG-16 showed the best performance.

Because detection-based methods aim to generate bounding boxes containing cracks, existing object detection methods, such as fast-RCNN, YOLO^21^ and SSD^22^, are often used for crack detection. Cha and Choi’s^16^ First Crack Detection Study Using Faster R-CNN. The faster RCNN is more accurate than the CNN and K-value models and can accurately locate cracks. Huyan et al.^23^ proposed a novel architecture called cju-NET that outperforms traditional image processing methods on test datasets. Ali et al.^24^ proposed an autonomous unmanned aerial vehicle (UAV) system integrated with an improved region-based faster convolutional neural network (Faster R-CNN) for identifying various types of structural damage, and map detected damage in GPS-denied environments. Meng et al.^25^ proposed an automatic real-time crack detection method based on UAV. The results show that this method can significantly improve the MIoU value of crack edge detection and the accuracy of maximum crack width measurement by automatically approaching the vicinity of the crack under non-ideal shooting conditions. Zeng et al.^26^ proposed a UAV damage detection task planning method based on Bayesian risk, which not only minimizes the UAV path length but also reduces the related SHM cost. Zhang et al.^27^ built a database containing many road damage images (e.g., strip cracks, web cracks, potholes, and ruts) and trained an improved SSD-Mobile Net deep neural network. They indicated that the improved method obtained a high average precision (AP). Zhang^28^ used an unmanned aerial vehicle (UAV) road damage database to enhance the utilization of basic features in You Only Look Once Version 3 (YOLO v3). Adding MLAB between the backbone network and the feature fusion part effectively increases the mAP value of the proposed network to 68.75%, while the accuracy of the original network is only 61.09%. Zheng^29^ proposed an improved crack detection method of YOLO v4 lightweight visual model, which meets the real-time operation requirements without sacrificing accuracy. The detection accuracy, recall rate and F1 score of this method are 93.96% respectively., 90.12% and 92%. Qu^30^ proposed an improved first-level target detector based on the YOLOv5 method, which improved the problem of missed detection and false detection of large targets, and had better detection results. Compared with the original model size YOLOv5_S, in PASCAL VOC and The mAP@ [0.5:0.95] on the MS COCO dataset is improved by 4.4% and 1.4% respectively. Ye et al.^31^ proposed an improved YOLOv7 network, and the experimental results show that the network not only can effectively detect crack images of different sizes, but also achieves satisfactory results in validating the robustness of noise contaminated images of different types and different intensities.

Segmentation-based methods are pixel-level crack detection methods in which each pixel is categorized as cracked or uncracked. It obtains pixel-level positional information, which allows for more important characterization information of the cracks to be obtained from the detection results. Lightweight real-time crack segmentation methods for cracks in complex background scenes have recently emerged. For example, Choi and Cha^32^ proposed an original convolutional neural network. Compared with the latest model, this model has 88 times fewer parameters than the latest model, but it can still obtain better evaluation indicators. Additionally, the model processes 1025 × 512 pixel real-time (36 FPS) images 46 times faster than recently developed images. Kang and Cha^33^ proposed a new semantic transformer representation network (STRNet) to solve the problem of real-time segmentation of pixel-level cracks in complex scenes. 1203 images were used for training, and further extensive comprehensive enhancement was performed using 545 Test images (1280 × 720, 1024 × 512) are studied; its performance is compared with recently developed advanced networks (Attention U-net, CrackSegNet, Deeplab V3+ , FPHBN, and Unet+ +), among which STRNet outperforms on the evaluation metrics Top performer—It achieved the fastest processing at 49.2 frames per second. Das and Leung et al.^34^ proposed a new deep learning model called strain hardening Segmentation Network (SHSnet). The calculation accuracy of this model is 85%, the required parameters are 1 order of magnitude less than the traditional model, and the calculation time is saved > 95%. Zhang et al.^35^ proposed a crack identification method based on improved U-Net. To detect cracks more accurately, they used the generalized die loss function. Zhu et al.^36^ proposed a multi-objective detection algorithm for pavement damage detection, which proved reasonable robustness in the classification and location of pavement damage. Das et al.^37^. employed a deep convolutional neural network to detect and pinpoint thin cracks in strain-hardened cementitious composites (SHCC) under real-world conditions. The model was trained on an NVIDIA 1060 6 GB GPU with a 227 × 227 × 3 image as the input size, and the training results showcased the network’s robustness and superiority in handling variations in image quality, adaptation to new datasets, and human-like level reasoning capabilities. Furthermore, they proposed an image processing technique to extract the crack density parameter from the outcomes of a custom deep convolutional neural network. Their findings demonstrated the network’s ability to accurately classify thin cracks in SHCC despite the presence of interfering factors such as sensors, markers, and inhomogeneous sample edges. Regardless of test configuration, structure size or geometry, cracks can be controlled below 100 μm, up to a few percent of strain. Li and Zhao^38^ introduced a high-resolution concrete damage image synthesis method based on Conditional Generative Adversarial Network (CGAN). Initially, they trained a low-resolution generator and GPU. They then utilized the low-resolution generator to synthesize a 512 × 1024 resolution low-resolution damage image, which was combined with the corresponding 1024 × 2048 resolution semantic high-resolution map to generate a high-resolution damage image at 1024 × 2048 resolution. The results show that the synthesized images have good realism and can be used for training and testing of concrete damage detection networks based on deep learning. Omar and Nehdi^39^ utilized Unmanned Aerial Vehicle (UAV) infrared thermography to detect concrete bridge decks. They achieved this by capturing thermal images using a high-resolution thermal imager during low altitude flights, analyzing the images using a k-means clustering tech-nique, and subsequently segmenting the spliced images and identifying the target thresholds. Their findings demonstrated that UAVs equipped with high-resolution thermal infrared imagery can effectively detect sub-surface anomalies in bridge decks without disrupting traffic flow, providing a valuable tool for rapidly assessing bridge conditions and conserving maintenance funds.

From the above research, it can be seen that classification-based, object detection and segmentation methods have been widely used in crack detection. However, due to different conditions, cracks on the concrete surface have unique characteristics, such as stains, impurities, etc. Different damages have different pixel ratios across the entire image, in order for the network to have better generalization capabilities when dealing with different sizes, shapes and types of cracks. Therefore, researchers proposed the Transformer architecture^40^. The transformer model employs a multi-head self-attention mechanism in order to obtain global connectivity between each pixel point and capture semantic associations over longer intervals. In order to improve efficiency while maintaining high accuracy, Krichene et al.^41^ proposed a new architecture, DoT, a dual-transformer model that jointly trains two transformers by optimizing task-specific losses. The results show that for accuracy For a small decrease, DoT improves training and inference times by at least 50%.The Vision Transformer proposed by Alexey Dosovitskiy et al.^42^ tested the Vision Transformer on the dataset ImageNet-21 k with 21 k categories and a total of 14 M images, and achieved better test results than ResNet. Therefore, it performs well in analyzing and predicting larger image data.

Vision Transformer is comparable to state-of-the-art convolutional networks and can be used to obtain similar features at both shallow and deep layers. In the image classification task, the image is classified by dividing it into small chunks, then joining these chunks into a sequence, and finally feeding this sequence into the Transformer. The advantage of Vision Transformer is that it can parallelise the training and has the ability to have global information. Nah et al.^43^ proposed the Swin transformer (Swin T), which reduces the complexity of operations through a technique that employs a local sliding window. Although global attention-based Transformers excel in performance, they are typically high in complexity and computationally intensive. However, local attention-based Transformers limit the interactions of each token’s receptive field, thus slowing down the growth of the receptive field. Therefore, when the resolution of the image is high, the data processing of ViT and SwinT will be low. Other researchers^44^ proposed a cross-window self-attention mechanism and devised the CSWin transformer (CSWT). In the self-attention module CSwin, a cross-shaped window self-attention mechanism is proposed, which can compute horizontal and vertical self-attention in parallel, thus obtaining better results with less computation. In addition, Locally Enhanced Position Encoding (LePE) can better handle local position information and support arbitrary shape inputs.The following differences exist between CSwin and Swin:

- (1) Facet delineation method: Swin uses non-overlapping convolutions of size 4 × 4 (step = 4) to delineate facets, while CSwin uses overlapping convolutions of size 7 × 7 (step = 4) to delineate facets.
  - (2) Window division method: Swin uses square windows for division, while CSwin uses horizontal and vertical rectangular windows for division, which improves the sensory field to some extent.
    - (3) Facet merging method: Swin uses interval extraction of elements and then concat for facet merging, while CSwin uses convolution of size 3 × 3 (stride = 2) instead of this method.

Due to its unique structural design, it is effective in terms of detection speed, but CSW is not accurate enough in identifying cracks in concrete structures in complex situations. In this paper, to overcome the limitations of the above networks, a new network, CSWin transformer-skip (CSW-S), is proposed based on CSWin transformer. CSW-S compensates for the slow detection and low accuracy of other network models. Cracks in concrete structures can be detected using this image classification technique, and it is expected to achieve better performance. The core structure of this paper is as follows. First, the model structure of CSW-S is described. Next, the details of the CSW-S dataset are provided, and the hyperparameter settings are listed. Then, the test results of concrete crack images are analyzed using the trained CSW-S. Finally, the performance and future work of the method are discussed.

The architecture of the proposed CSW-S.

**Figure 1:**
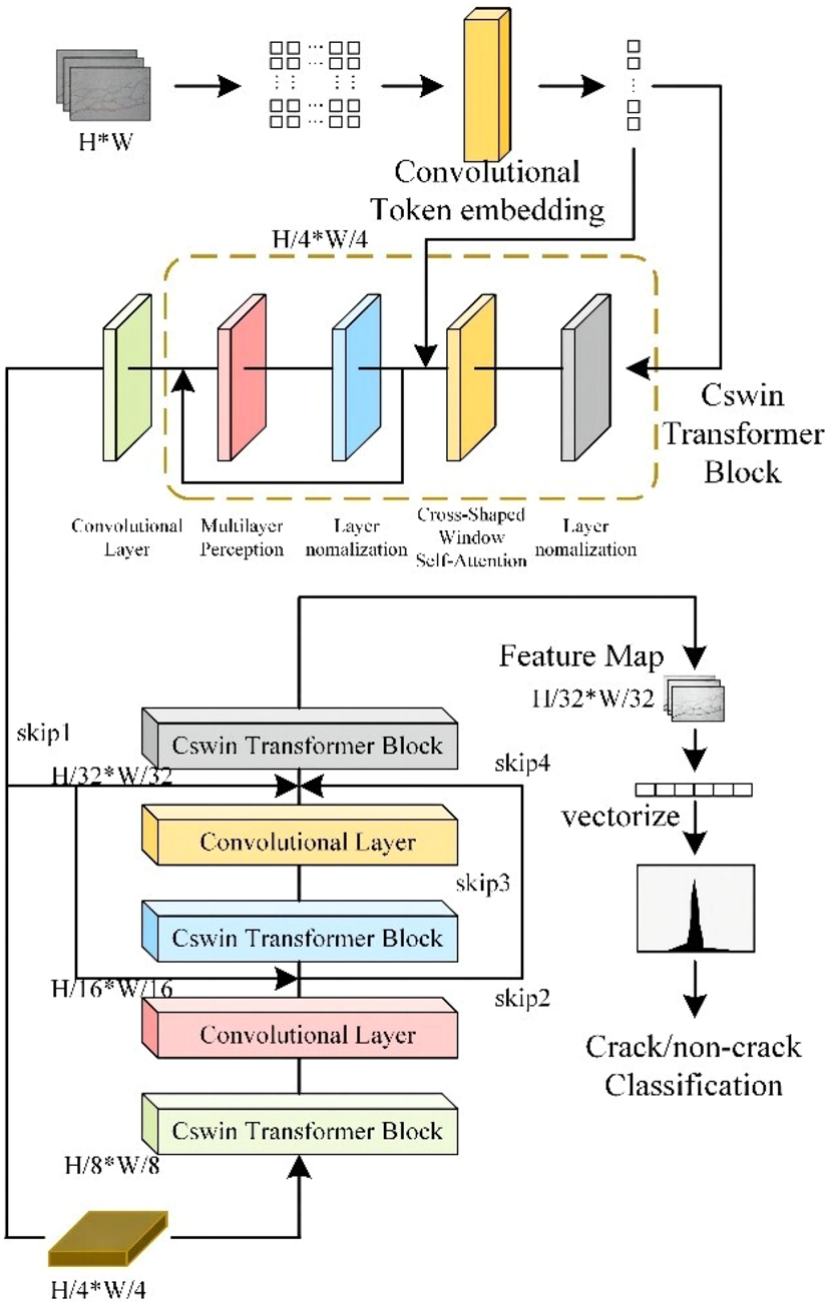
Framework of CSwin Transformer-skip.

Based on the research of He K. et al.^45^, the CSW-S architecture is redesigned, as shown in Fig. 1. This architecture consists of three parts: a convolutional token embedding layer, a CSWin transformer block, and a convolutional layer. In the convolutional token embedding layer, a convolutional layer with overlap (step size 4, convolutional kernel size 7 × 7) is used to transform the input into a patch token of size H/4*W/4.

The result is then fed into four identical CSWin transformer blocks, and the autonomy mechanism in the blocks is used to extract the information in the image. Then, the features are extracted. In the convolutional layer, the image size is modified, the extracted feature maps is vectorized, and the values are mapped to (0,1) probabilities to classify the image information. In addition, we modify the connectivity of the network structure by adding residual links to enable information from different layers to flow, effectively improving the network performance. The details of the specific network structure are shown in Table 1.

**Table 1:** CSW-S network structure details.

Structure	Input		Convolution kernel	Passage	Step length	
Convolutional token enbedding	24 × 24 × 3		7 × 7	64	54	
Structure	Input	depth	number head	dim	reso	split-size
Stage1	3136 × 64	1	2	64	56	1
Stage2	3136 × 64	2	4	128	28	2
Stage3	3136 × 64	2	8	256	14	7
Stage4	3136 × 64	1	16	512	7	7

Dim refers to the dimension of the fully connected network spread into and number head the number of multi-headed attention heads. Each head is responsible for different correlations. The resolution is the size of the picture before it is spread into a vector, and depth is the number of repetitions.

### Cross-shaped window self-attention

**Figure 2:**
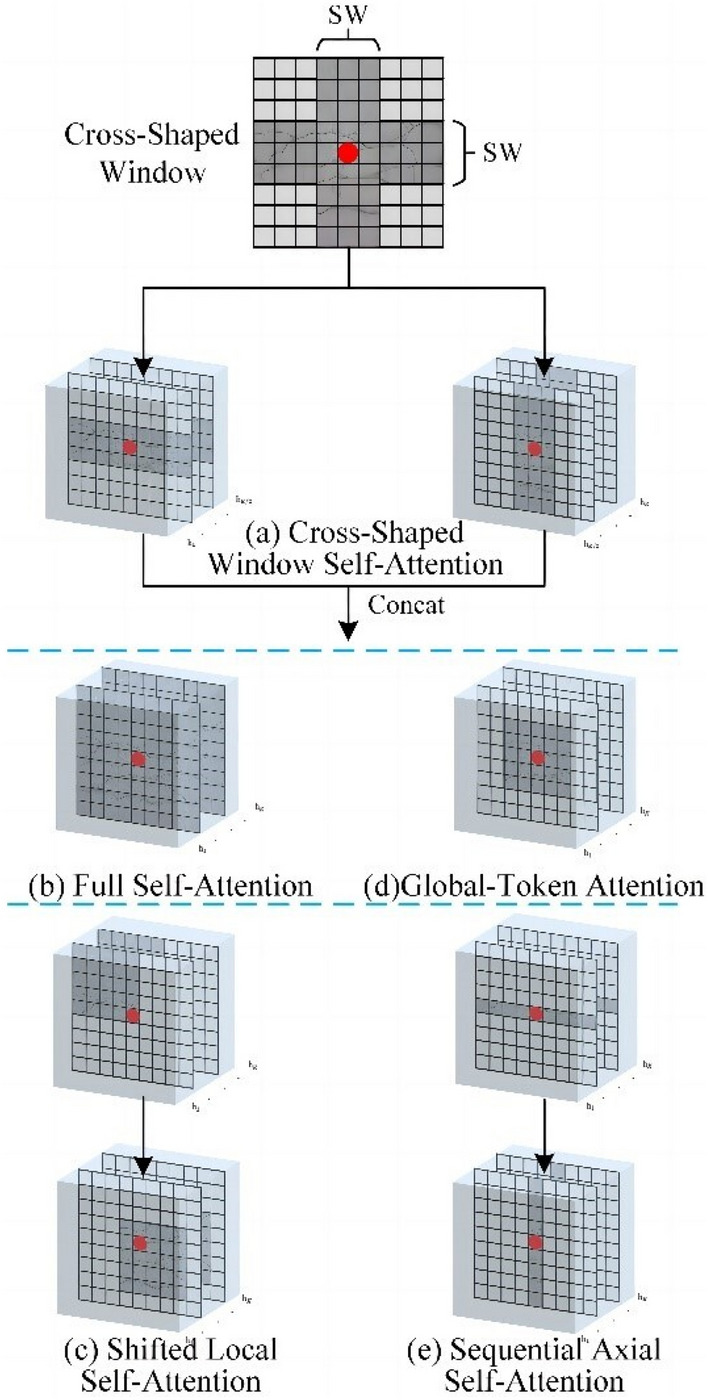
Cross-window self-attention mechanism.

To expand the perceptual field of the transformer without computing global self-attention, the current mainstream practice is to compute self-attention for the transformer of local attention and then expand the perceptual field by a shift window. However, the token in each self-attention block is still a limited self-attention region. Many blocks must be stacked to fuse more feature information and thus achieve a global perceptual field. This brings the problem of a more significant number of parameters and more complex computational difficulty. In the latest research in computer vision, cross-shaped window self-attention is used to solve this problem. Most networks, such as the Swin transformer, only use the self-attention mechanism, and only one small square of information can be extracted at a time in the face of image feature extraction. The cross-shaped window self-attention used in this model can be used to extract the image features within the horizontal or vertical stripes to expand the attention range of tokens within a transformer’s block, as shown in Fig. 2. Each stripe is obtained by splitting the input features into equal-width stripes. The effect of stripe width is mathematically analyzed, and the stripe width is varied for different layers of the transformer network, enabling powerful modeling capabilities while limiting the computational cost.

### Locally-enhanced positional encoding

In this paper, to compensate for the shortcomings of self-attention (SA), a new locally-enhanced positional encoding (LePE) is used. Unlike APE and CPE in vision transformer, in which location information is directly added to the input token in self-attention and then sent into the vision transformer, in Swin transformer, RPE is used to embed location information directly into the correlation calculation of the transformer block. LePE is used to learn the location information of a value directly using deep convolution, which is then summed with residuals and embedded it into the transformer block very conveniently. The position of the value is encoded directly, and the position information is fused into the SA after a matrix operation. Moreover, the depthwise convolution of the values is used to adjust the size of the parameters and make the calculation easier.

### Residual connections

In engineering inspection, especially in some larger projects, the amount of data is often quite large. CSWin transformer is a highly accurate target detection network due to its relatively highly complex structure. As the depth of the network increases, gradient dissipation and gradient explosion may occur. Therefore, this study aims to improve the performance and convergence of the neural network while using the network’s powerful ability to extract the target features. Local low-level features in the shallow layer are retained to enhance the high-level features in the deep layer. The modified network in this paper can better fuse the information between the shallow and deep layers and thus avoid spurious gradient explosion and gradient disappearance problems.

**Figure 3:**
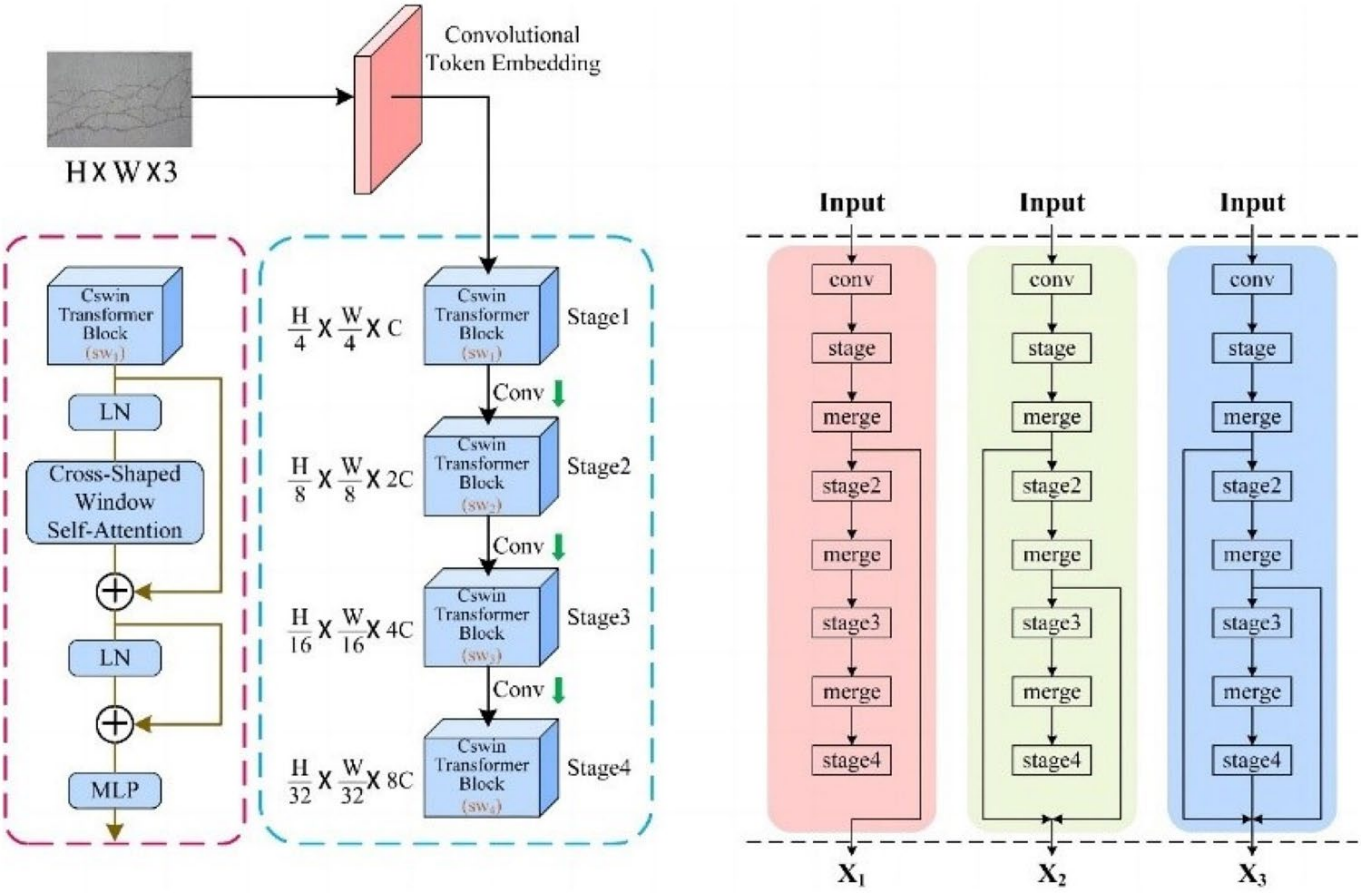
Residual linking process.

Therefore, a skip connection is added to the original skeleton, as shown in Fig. 3. First, the features between stage 1 and stage 2, the features between stage 2 and stage 3, and the features of the layering stage 4 are fused. The result is fused again with the three features in the first stage, the first two features in the second stage, and directly with the features in the first step of the third stage and then output. The use of several stages can better capture long-range dependencies and global information. It also allows the convolutional layers to be stacked more abstractly and the information to be more condensed.

## Classification

To classify the input data, a softmax layer is essential. The softmax layer uses the softmax function given in Eq. (1) to estimate the likelihood p(y(i) = j(i) | x(i);w) for each of the k classes j(i), where w is the weight, i is the i-th example of the n input examples, and ${W}_{K}^{T}{x}^{(i)}$ is the input to the softmax layer. The right-hand side of the equation is a k-dimensional vector that represents the k estimated likelihoods that $1/{\sum }_{j=1}^{k}{e}^{{W}_{J}^{T}{X}^{(i)}}$ to normalize the probability distribution.

1 $$\begin{array}{c}p({y}^{(i)}={j}^{(i)}\mid {x}^{(i)};W)=\left[\begin{array}{c}p({y}^{(i)}=1\mid {x}^{(i)};W)\\ p({y}^{(i)}=2\mid {x}^{(i)};W)\\ \vdots \\ p({y}^{(i)}=k\mid {x}^{(i)};W)\end{array}\right]\\ =\frac{1}{\sum_{j=1}^{k}{e}^{{W}_{j}^{T}{x}^{(i)}}}\left[\begin{array}{c}{e}^{{W}_{1}^{T}{x}^{(\varphi )}}\\ {e}^{{W}_{2}^{T}{x}^{(i)}}\\ \vdots \\ {e}^{{W}_{k}^{T}{x}^{(i)}}\end{array}\right],\\ i=1,\dots ,N\end{array}$$

## Training and performance evaluation

### Data set

In addition, in the process of crack recognition, the training of neural network requires a sufficient number of images, if the number of trained images is not enough, it may limit the application in practice. Yang et al.^46^ used 278 spalling and 954 crack images and adopted VGG-16 CNN architecture for training, and the classification accuracy of this method on the SCCS database reached 93.36%. Zou et al.^47^ built a DeepCrack network on SegNet’s encoder-decoder architecture and trained 3500 images with size of 512 × 512. achieves F-measure over 0.87 on the three challenging datasets in average. Achieves f-measure over 0.87 on the three challenging datasets in average. Silva and Lucena et al.^48^ developed a deep learning crack detection model based on CNN, which was trained using a dataset containing 3500 concrete surface images and obtained an accuracy of 92.27%. Therefore, while designing better network models, increasing the number of trained crack images will help to improve the application of network models in practical engineering.

In this paper, a dataset was prepared using a multipurpose pavement inspection vehicle to detect cracks and acquire images. The database contains 8000 images of concrete pavement cracks of size 4000 4000. These images were cropped to 224 pixels 224 pixels, and a dataset with 17,000 cropped cracked images was obtained. For faster training and testing of CSW-S, the cracks were numbered and manually labeled with crack contours to construct the pavement crack detection dataset. The dataset was randomly divided into a training set of 11,900 images and a test set of 5100 images at a ratio of 7:3.

**Figure 4:**
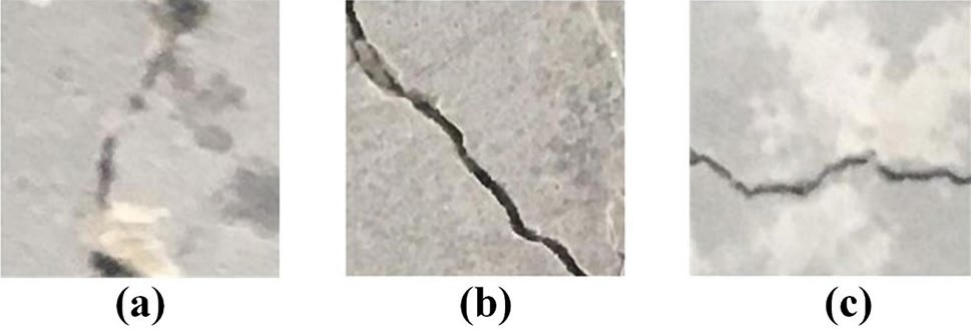
Visualized (**a**) blurred images, (**b**) clear images, (**c**) overexposed images.

The transformer model was used to complete the binary classification problem. The photos must be manually classified into two categories (cracked and uncracked). In order to make the classification of the transformer model more accurate, the training set and the test set contain photos of various forms of cracks with impurities, dark spots, and graffiti as shown in Table 2. All these photos were used in the transformer model to extract and learn the characteristic disturbance information of cracks. The Transformer model uses the dataset shown in Fig. 4, which we visualized. The CSW-S model is used to extract the crack features hidden in the photos to achieve crack classification.

**Table 2:** Types of images in the dataset.

Dataset	Image size	Training set	Validation set	Total number
Crack data set	224^2^	11,900	5100	17,000
Longitudina	224^2^	3566	1558	5124
Transverse	224^2^	5962	2591	8553
Fatigue	224^2^	2372	951	3323

### Hyper-parameter

The goal of training the CSWin transformer-skip was to increase the training data’s variability and avoid overfitting. The dropout method was used between two fully connected layers to reduce overfitting by preventing complex adaptation to the training data. The output of each neuron was set to zero with a probability of 0.5. The graphics processing unit (GPU) was used to accelerate the training of the CSWin transformer. Further acceleration was achieved by using a rectified linear unit (ReLU) as the activation function, which is more effective than the hyperbolic tangent (tanh) and sigmoid functions used in traditional neuronal models for training and evaluation. The neural network was trained using the stochastic gradient descent (SGD) method, and the PyTorch deep learning framework was used to train the CSWin transformer-skip. In training, the initial global learning rate was set to 1e–5, and the weight decay was set to 0.0005. The stochastic gradient descent (SGD) method was used to update the network parameters with a minimum batch size of 2 for each iteration. A total of 230 iterations were used to train the network. All experiments in this paper were performed using a single QuadroRTXA5000 GPU.

## Testing and discussions.

To evaluate the performance of the proposed model, we conducted tests on the dataset provided in Section. “Training and performance evaluation” and validated the network performance through three experiments: investigating the network hyperparameters, the dataset size, and comparing it with different network models, respectively. This section briefly outlines the general flow of each method.

### Evaluation

To quantitatively assess the performance of the model, several evaluation factors commonly used in classification tasks were used. These include the true positive (TP), true negative (TN), false-positive (FP), and false-negative (FN). True positives are samples that were correctly classified as cracked, and true negatives are samples that were correctly classified as uncracked. Similarly, false-positives are samples that were uncracked but incorrectly classified as cracked by the network. False-negatives are cracked samples incorrectly classified as uncracked by the network. Table 2 shows the network’s accuracy, precision, and completeness. Recall can be interpreted as the percentage of crack samples identified by the network to the total number of cracks in the dataset. Accuracy, on the other hand, is the percentage of predicted cracks that are cracked. The accuracy, accuracy, and completeness are calculated as follows:

2 $$Accuracy=\frac{TP+TN}{TP+TN+FP+FN}$$

3 $$Presion=\frac{TP}{TP+FP}$$

4 $$Recall=\frac{TP}{TP+TN}$$

5 $$Specificity=\frac{TN}{TN+TP}$$

6 $$F1score = 2 \times \frac{Presion \cdot Recall}{{Presion + Recall}}$$

7 $$MIOU = \frac{1}{2}\left( {\frac{TP}{{TP + FN + FN}} + \frac{TN}{{TN + FP + FN}}} \right)$$

### Training and validation

**Figure 5:**
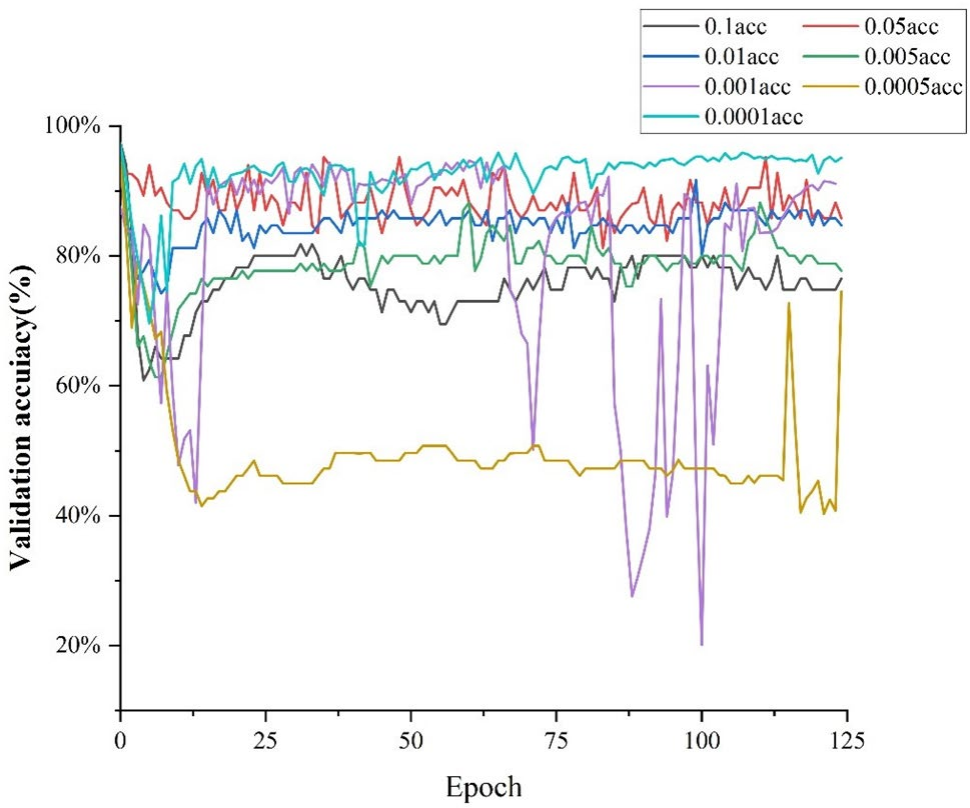
Training results for different learning rates.

During the training of CSW-S, the learning rate affects the verification accuracy and convergence speed. To select the best base learning rate, the base learning rates used in this paper were set to 0.1, 0.05, 0.01, 0.005, 0.001, 0.0005 and 0.0001. The CSW-S was trained for 124 epochs at different base learning rates, and validation was performed once after each epoch. The recorded confirmation accuracies are shown in Fig. 5.

According to Fig. 5, the training accuracy and convergence speed as a whole decreases as the base learning rate varies from 0.0001 to 0.1, and peaks at 0.0001. When the base learning rate is 0.0005, this will lead to an increase in accuracy first and then remain at 45%, indicating that the training of CSW-S is non-converging. The key finding from the training results is that choosing a small base learning rate within a certain range allows the CNN to converge faster during training and achieve higher verification accuracy.

As the highest accuracy of 95.9% was achieved after the 109th epoch training, the training result at a base learning rate of 0.0001 was finally chosen as the image classifier.

### Effect of dataset size on model performance

**Figure 6:**
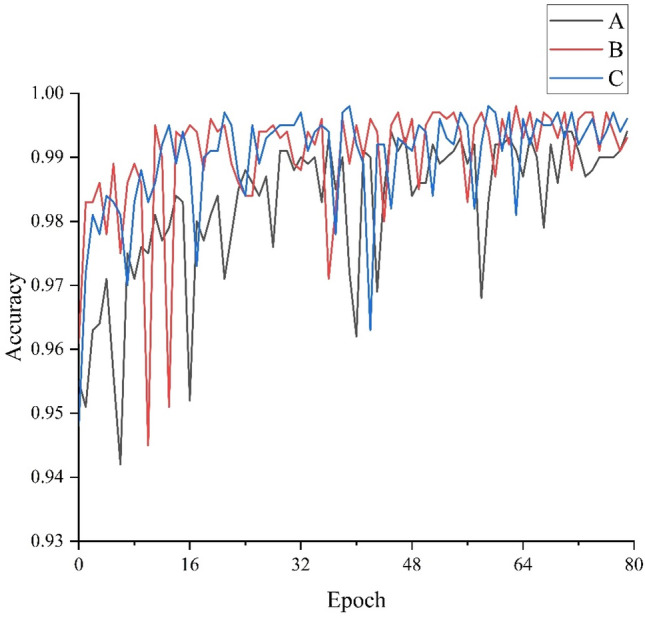
Network training graphs for different dataset Model Comparison.

To investigate the effect of dataset size on model performance. There are 3 datasets A, B, C. 1000, 2000, 3000 images were randomly selected from all the images respectively. To ensure comparability of the model results, the ratio of the number of cracks to the background was kept consistent across the three datasets. As presented in Section. “The architecture of the proposed CSW-S”, the proposed model was trained and tested with three different datasets by validating the proposed model. The results show that the Accuracy values of the model increase continuously as the size of the dataset increases. This is shown in Fig. 6. The model using dataset C has the highest curve for each defect detection and represents the best performance.

Five network models were chosen to compare with the CSW-S network model: two classical convolutional neural networks, GoogLeNet and AlexNet, and three artificial neural networks using self-attention, vision transformer (ViT), Swin transformer (SwinT), and CSWin transformer (CSW). Table 3 show the results of the quantitative comparison between the different methods.

**Table 3:** Evaluation on crack detection.

Methods	Accuracy	Precsion	Recall	Specificity	F1score	MIOU
ViT	78.15%	91.59%	62%	94.31%	73.94%	63.50%
GoogleNet	90.47%	88.08%	93.60%	87.33%	90.75%	82.58%
AlexNet	94.92%	92.98%	**97.17%**	92.66%	95.02%	90.32%
CSW	95.66%	97.35%	93.88%	97.45%	95.58%	91.69%
SwinT	96.15%	95.44%	**96.94%**	95.37%	96.18%	92.59%
CSW-S	96.92%	98.65%	95.13%	98.70%	96.85%	94.03%

Significant values are in bold.

ViT networks have three parts: an embedding layer, a transformer encoder layer, and an MLP head layer. ViT differs significantly from a convolutional neural network (CNN) in the way it processes features. The input image is first divided into 16 × 16 patches and fed into the vision transformer network. Apart from the first layer embedding, there are no convolutional operations in ViT, and interactions between different locations are only achieved through the self-attention layer. CNNs have limitations in modeling global information due to limitations in the size of the convolutional kernel. ViT allows all locations in the image to interact globally through self-attention. ViT demonstrates that a transformer pretrained on a large dataset, without modifications for the visual task, can perform similarly or even better than the best CNNs. The resulting experiments yielded performance metrics of 78.15%, 62%, and 73.94% for accuracy, recall, and F1 score, respectively.

In GoogLeNet, a multiscale fusion network structure, in which an inception structure is introduced to fuse feature information at different scales, is proposed. A 1*1 convolutional kernel is also used for dimensionality reduction and mapping. The fully connected layer is discarded in favor of an average pooling layer, significantly reducing the model parameters. As a result, not only is the accuracy rate very high but the number of parameters is lower than in other convolutional neural networks, effectively preventing overfitting. The resulting experiments yielded performance metrics of 90.47%, 93.60%, and 90.75% for accuracy, recall, and F1 score, respectively.

A crack detection method based on the convolutional neural network AlexNet has been carried out in many experiments. AlexNet has an eight-layer structure. The first five layers are convolutional layers, and the last three layers are fully connected layers. Compared to previous models, ResNet has deeper layers, pioneers the use of ReLU as a nonlinear mapping function, and can be trained using multiple CPUs. This effectively solves the problem of overfitting that other previous networks were prone to and achieves higher recognition accuracy. However, as with other earlier networks, when the values of the input model are very large or very small, saturation occurs, i.e., the gradient of neurons approaches 0, and therefore, the gradient disappearance problem occurs. Therefore, it is often difficult to process more complex image data. The resulting performance metrics for accuracy, recall, and F1 score were 94.92%, 97.17%, and 95.02%, respectively.

The Swin transformer draws on many design concepts and prior knowledge of convolutional neural networks. The sequence length and computational complexity are by computing self-attention within a small window. In addition, a pooling-like operation called patch merging, where four adjacent small patches are combined into one large patch to obtain features of different sizes, is proposed. The resulting experiments yielded performance metrics of 96.15%, 96.94%, and 96.18% for accuracy, recall, and F1 score, respectively.

Table 3 shows that compared with other network models, an accuracy of 96.92%, a precision of 98.65%, a specificity of 98.70%, an F1 score of 96.85%, and an MIoU of 94.03% were achieved using the proposed network model, which was better than the other network models. However, the recall, which was 95.13%, was lower than that of AlexNet (97.17%) and CSW (96.94%). In particular, the accuracy, precision, recall, specificity, F1 score, and MIoU improved by 1.26%, 1.3%, 1.25%, 1.25%, 1.27%, and 2.34%, respectively, using the proposed CSW-S compared to CSW. The experimental results demonstrated the superior performance of the proposed CSW-S for concrete crack classification and proved the effectiveness of the CSW-based improvements. However, in terms of detection speed, although there was a decrease in the FPS of the improved model, it had little impact on practical inspection, as the detection accuracy is more important than the detection speed for the detection of cracks in concrete.

## Conclusion

This paper presents a crack detection framework based on CSWin transformer for crack detection of concrete pavement. The image dataset consists of 1000 images with 4000 × 4000 pixel resolution, cropped to 224 × 224 pixels, resulting in 17,000 cracked cropped image datasets. In order to ensure the performance of the network, we adjusted the network by designing different parameters and obtained the highest accuracy of 95.9%. In order to investigate the effect of data set size on model performance, we randomly selected 1000,2000,3000 images with cracks from the data set we made, and tested the proposed network model. It was proved that the accuracy value of our network increased with the increase of data set size. Because the methodology of this paper is intended to provide quality assessment and monitoring of newly constructed concrete pavements, the photographs of cracks in the selected dataset were taken under conditions free of complex environmental factors (such as obstacles like leaves, rubbish, etc.). The method is only suitable for testing concrete pavements with surfaces free of obstructions. It may not be able to provide accurate results in the case of complex pavement environments.

In the proposed CSW-S model, multiple fusion residual links are added, and compared with other networks, the model has extremely high feature extraction capability on the target, while capturing remote dependencies and global information better. Using this method, the shallow and deep image information is better fused to produce more concentrated fracture characteristics and reduce model computational complexity, gradient explosion, and gradient disappearance. Compared with the existing commonly used network framework, the accuracy of the model can reach 96.92%, the accuracy can reach 98.65%, and the F1 score can reach 96.85% without pre-training. Therefore, the model can be used as a new technology and method for crack identification in the health monitoring of concrete pavement engineering. In future studies, more images of concrete damage types under different conditions will be added to the existing database to improve the adaptability and robustness of the proposed method, and comparative studies will be conducted.

### Supplementary Information

Supplementary Information 1.

Supplementary Information 2.

Supplementary Information 3.

Supplementary Information 4.

## Data Availability

The datasets used and/or analysed during the current study available from the corresponding author on reasonable request.

## References

[1] Chen F, Jahanshahi MR, Wu R, Joffe C (2017). A texture-Based video processing methodology using bayesian data fusion for autonomous crack detection on metallic surfaces. Comput. Aided Civ. Eng..

[2] Golewski GL (2023). The Phenomenon of cracking in cement concretes and reinforced concrete structures: The mechanism of cracks formation, causes of their initiation, types and places of occurrence, and methods of detection—A review. Buildings.

[3] Huang J, Liu W, Sun X (2014). A pavement crack detection method combining 2D with 3D information based on dempster-shafer theory. Comput. Aided Civ. Eng..

[4] Gavilán M (2011). Adaptive road crack detection system by pavement classification. Sensors.

[5] Jahanshahi MR, Jazizadeh F, Masri SF, Becerik-Gerber B (2013). Unsupervised approach for autonomous pavement-defect detection and quantification using an inexpensive depth sensor. J. Comput. Civ. Eng..

[6] Jo Y, Ryu S (2015). Pothole detection system using a black-box camera. Sensors.

[7] Radopoulou SC, Brilakis I (2017). Automated detection of multiple pavement defects. J. Comput. Civ. Eng..

[8] Zhang D (2018). Automatic pavement defect detection using 3D laser profiling technology. Autom. Constr..

[9] Bursanescu, L., Bursanescu, M., Hamdi, M., Lardigue, A. & Paiement, D. Three-dimensional infrared laser vision system for road surface features analysis. In *Presented at the ROMOPTO 2000: Sixth Conference on Optics* (ed. Vlad, V. I.) 801 10.1117/12.432808 (Bucharest, Romania, 2001).

[10] Abu-Mahfouz I, Banerjee A (2017). Crack detection and identification using vibration signals and fuzzy clustering. Proced. Comput. Sci..

[11] Suzuki T, Aoki M (2010). Damage Identification Of Cracked Concrete by X-Ray Computed Tomography Method.

[12] Das AK, Leung CK (2022). Fast tomography: A greedy, heuristic, mesh size–independent methodology for local velocity reconstruction for AE waves in distance decaying environment in semi real-time. Struct. Health Monit..

[13] Heaton J (2018). Ian Goodfellow, Yoshua Bengio, and Aaron Courville: Deep learning: The MIT Press, 2016, 800 pp, ISBN: 0262035618.. Genet. Progr. Evolv. Mach..

[14] Zhang L, Yang F, Daniel Zhang Y, Zhu YJ, Zhang L, Yang F, Daniel Zhang Y, Zhu YJ (2016). Road crack detection using deep convolutional neural network. 2016 IEEE International Conference on Image Processing (ICIP).

[15] Cha Y, Choi W, Büyüköztürk O (2017). Deep learning-based crack damage detection using convolutional neural networks. Comput. Aided Civ. Eng..

[16] Cha Y, Choi W, Suh G, Mahmoudkhani S, Büyüköztürk O (2018). Autonomous structural visual inspection using region-based deep learning for detecting multiple damage types. Comput. Aided Civ. Eng..

[17] Dorafshan S, Thomas RJ, Maguire M (2018). Comparison of deep convolutional neural networks and edge detectors for image-based crack detection in concrete. Constr. Build. Mater..

[18] Yang X (2018). Automatic pixel-level crack detection and measurement using fully convolutional network. Comput. Aided Civ. Eng..

[19] Li R, Yuan Y, Zhang W, Yuan Y (2018). Unified vision-based methodology for simultaneous concrete defect detection and geolocalization. Comput. Aided Civ. Eng..

[20] Gopalakrishnan K, Khaitan SK, Choudhary A, Agrawal A (2017). Deep convolutional neural networks with transfer learning for computer vision-based data-driven pavement distress detection. Constr. Build. Mater..

[21] Redmon, J., Divvala, S., Girshick, R. & Farhadi, A. You only look once: Unified, real-time object detection. In *Proceedings of the IEEE conference on computer vision and pattern recognition* 779–788 (2016).

[22] Leibe, B., Matas, J., Sebe, N., Welling, M. (Eds.) 2016. In *Proc. Computer Vision – ECCV 2016: 14th European Conference*, Amsterdam, The Netherlands, October 11–14, 2016, Part I, Lecture Notes in Computer Science. 10.1007/978-3-319-46448-0 (Springer International Publishing, Cham, 2016)

[23] Huyan J, Li W, Tighe S, Xu Z, Zhai J (2020). CrackU-net: A novel deep convolutional neural network for pixelwise pavement crack detection. Struct. Control Health Monit..

[24] Ali R, Kang D, Suh G, Cha Y-J (2021). Real-time multiple damage mapping using autonomous UAV and deep faster region-based neural networks for GPS-denied structures. Autom. Constr..

[25] Meng S, Gao Z, Zhou Y, He B, Djerrad A (2023). Real-time automatic crack detection method based on drone. Comput. Aided Civ. Eng..

[26] Zeng J, Wu Z, Todd MD, Hu Z (2023). Bayes risk-based mission planning of unmanned aerial vehicles for autonomous damage inspection. Mech. Syst. Signal Process..

[27] Zhang K, Li H, Wang Z, Zhao X, Farhangdoust S, Meyendorf NG (2020). Feature recognition and detection for road damage based on intelligent inspection terminal. Smart Structures and NDE for Industry 4.0, Smart Cities, and Energy Systems.

[28] Zhang Y (2022). Road damage detection using UAV images based on multi-level attention mechanism. Autom. Constr..

[29] Zhang J, Qian S, Tan C (2023). Automated bridge crack detection method based on lightweight vision models. Complex Intell. Syst..

[30] Qu Z, Gao L, Wang S, Yin H, Yi T (2022). An improved YOLOv5 method for large objects detection with multi-scale feature cross-layer fusion network. Image Vis. Comput..

[31] Ye G (2023). Autonomous surface crack identification of concrete structures based on the YOLOv7 algorithm. J. Build. Eng..

[32] Choi W, Cha Y-J (2020). SDDNet: Real-time crack segmentation. IEEE Trans. Ind. Electron..

[33] Kang DH, Cha Y-J (2022). Efficient attention-based deep encoder and decoder for automatic crack segmentation. Struct. Health Monit..

[34] Das AK, Leung CKY, Kunieda M, Kanakubo T, Kanda T, Kobayashi K (2023). A novel deep learning model for end-to-end characterization of thin cracking in SHCCs. Strain Hardening Cementitious Composites.

[35] Zhang L, Shen J, Zhu B (2021). A research on an improved Unet-based concrete crack detection algorithm. Struct. Health Monit..

[36] Zhu J (2022). Pavement distress detection using convolutional neural networks with images captured via UAV. Autom. Constr..

[37] Das AK, Leung C, K. Y. & Wan, K. T.  (2021). Application of deep convolutional neural networks for automated and rapid identification and computation of crack statistics of thin cracks in strain hardening cementitious composites (SHCCs). Cem. Concr. Compos..

[38] Li S, Zhao X (2023). High-resolution concrete damage image synthesis using conditional generative adversarial network. Autom. Constr..

[39] Omar T, Nehdi ML (2017). Remote sensing of concrete bridge decks using unmanned aerial vehicle infrared thermography. Autom. Constr..

[40] Vaswani, A. *et al*. Attention is all you need. *Adv. Neural Inf. Proc. Syst.* **30** (2017).

[41] Krichene, S., Mueller, T. & Eisenschlos, J. DoT: An efficient Double Transformer for NLP tasks with tables. In *Findings of the Association for Computational Linguistics: ACL-IJCNLP 2021*. 3273–3283 (2021).

[42] Dosovitskiy, A. *et al.* An image is worth 16x16 words: Transformers for image recognition at scale. arXiv preprint arXiv:2010.11929, 2020.

[43] Nah S, Kim TH, Lee KM, Nah S, Kim TH, Lee KM (2017). Deep multi-scale convolutional neural network for dynamic scene deblurring. 2017 IEEE Conference on Computer Vision and Pattern Recognition (CVPR).

[44] Cai, Z. *et al.* A unified multi-scale deep convolutional neural network for fast object detection. In *Proc. Computer Vision–ECCV 2016: 14th European Conference*, Amsterdam, The Netherlands, October 11–14, 2016, Part IV 14. 354–370 (Springer International Publishing, 2016).

[45] He K, Zhang X, Ren S, Sun J, He K, Zhang X, Ren S, Sun J (2016). Deep residual learning for image recognition. 2016 IEEE Conference on Computer Vision and Pattern Recognition (CVPR).

[46] Yang L, Yang L (2017). A robotic system towards concrete structure spalling and crack database. 2017 IEEE International Conference on Robotics and Biomimetics (ROBIO).

[47] Zou Q (2019). DeepCrack: Learning hierarchical convolutional features for crack detection. IEEE Trans. Image Process..

[48] Silva WRLD, Lucena DSD, Silva WRLD, Lucena DSD (2018). Concrete cracks detection based on deep learning image classification. The 18th International Conference on Experimental Mechanics.

