# Supplementary figures and images for: An improved transformer-based concrete crack classification method (part 2 of 7)
Source: Sci Rep. 2024 Mar 14;14:6226. doi: 10.1038/s41598-024-54835-x (PMC10940720; doi:10.1038/s41598-024-54835-x)

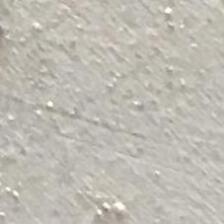

Supplement: Supplementary file 1 — Supplementary Information 1. [file 41598_2024_54835_MOESM1_ESM.zip › 5000/train/Negative/00561.jpg]

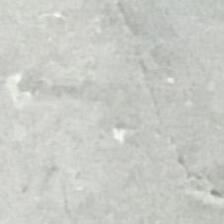

Supplement: Supplementary file 1 — Supplementary Information 1. [file 41598_2024_54835_MOESM1_ESM.zip › 5000/train/Negative/00562.jpg]

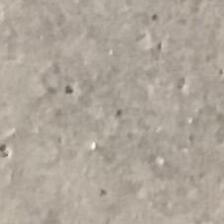

Supplement: Supplementary file 1 — Supplementary Information 1. [file 41598_2024_54835_MOESM1_ESM.zip › 5000/train/Negative/00563.jpg]

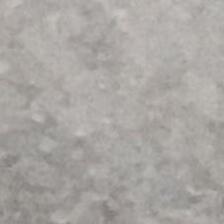

Supplement: Supplementary file 1 — Supplementary Information 1. [file 41598_2024_54835_MOESM1_ESM.zip › 5000/train/Negative/00564.jpg]

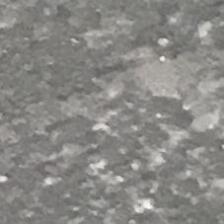

Supplement: Supplementary file 1 — Supplementary Information 1. [file 41598_2024_54835_MOESM1_ESM.zip › 5000/train/Negative/00565.jpg]

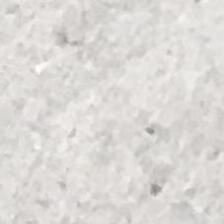

Supplement: Supplementary file 1 — Supplementary Information 1. [file 41598_2024_54835_MOESM1_ESM.zip › 5000/train/Negative/00566.jpg]

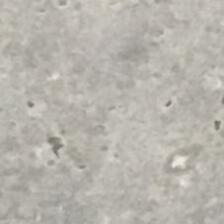

Supplement: Supplementary file 1 — Supplementary Information 1. [file 41598_2024_54835_MOESM1_ESM.zip › 5000/train/Negative/00567.jpg]

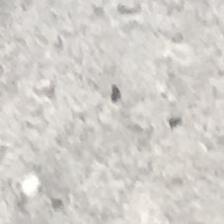

Supplement: Supplementary file 1 — Supplementary Information 1. [file 41598_2024_54835_MOESM1_ESM.zip › 5000/train/Negative/00568.jpg]

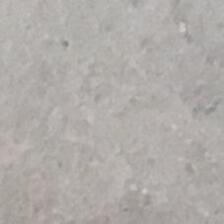

Supplement: Supplementary file 1 — Supplementary Information 1. [file 41598_2024_54835_MOESM1_ESM.zip › 5000/train/Negative/00569.jpg]

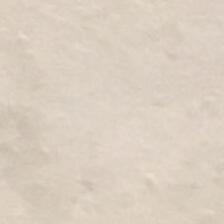

Supplement: Supplementary file 1 — Supplementary Information 1. [file 41598_2024_54835_MOESM1_ESM.zip › 5000/train/Negative/00570.jpg]

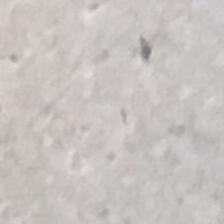

Supplement: Supplementary file 1 — Supplementary Information 1. [file 41598_2024_54835_MOESM1_ESM.zip › 5000/train/Negative/00571.jpg]

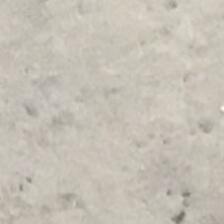

Supplement: Supplementary file 1 — Supplementary Information 1. [file 41598_2024_54835_MOESM1_ESM.zip › 5000/train/Negative/00572.jpg]

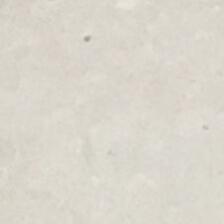

Supplement: Supplementary file 1 — Supplementary Information 1. [file 41598_2024_54835_MOESM1_ESM.zip › 5000/train/Negative/00573.jpg]

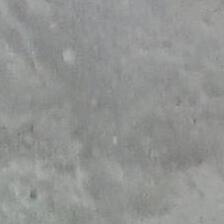

Supplement: Supplementary file 1 — Supplementary Information 1. [file 41598_2024_54835_MOESM1_ESM.zip › 5000/train/Negative/00574.jpg]

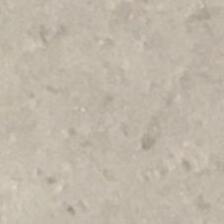

Supplement: Supplementary file 1 — Supplementary Information 1. [file 41598_2024_54835_MOESM1_ESM.zip › 5000/train/Negative/00575.jpg]

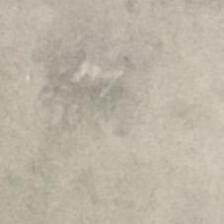

Supplement: Supplementary file 1 — Supplementary Information 1. [file 41598_2024_54835_MOESM1_ESM.zip › 5000/train/Negative/00576.jpg]

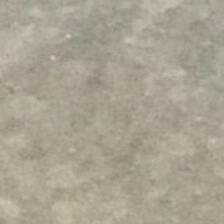

Supplement: Supplementary file 1 — Supplementary Information 1. [file 41598_2024_54835_MOESM1_ESM.zip › 5000/train/Negative/00577.jpg]

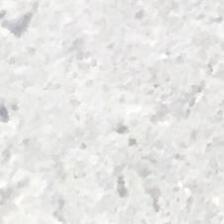

Supplement: Supplementary file 1 — Supplementary Information 1. [file 41598_2024_54835_MOESM1_ESM.zip › 5000/train/Negative/00578.jpg]

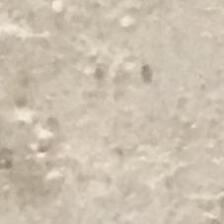

Supplement: Supplementary file 1 — Supplementary Information 1. [file 41598_2024_54835_MOESM1_ESM.zip › 5000/train/Negative/00579.jpg]

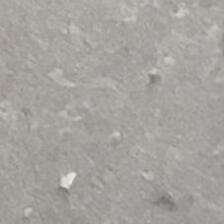

Supplement: Supplementary file 1 — Supplementary Information 1. [file 41598_2024_54835_MOESM1_ESM.zip › 5000/train/Negative/00580.jpg]

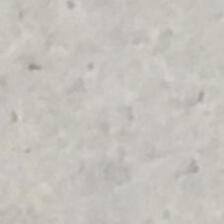

Supplement: Supplementary file 1 — Supplementary Information 1. [file 41598_2024_54835_MOESM1_ESM.zip › 5000/train/Negative/00581.jpg]

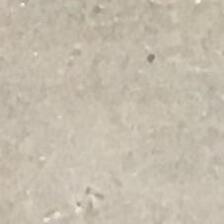

Supplement: Supplementary file 1 — Supplementary Information 1. [file 41598_2024_54835_MOESM1_ESM.zip › 5000/train/Negative/00582.jpg]

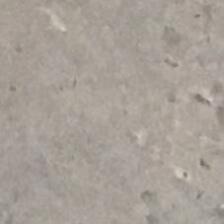

Supplement: Supplementary file 1 — Supplementary Information 1. [file 41598_2024_54835_MOESM1_ESM.zip › 5000/train/Negative/00583.jpg]

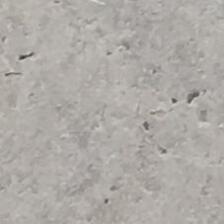

Supplement: Supplementary file 1 — Supplementary Information 1. [file 41598_2024_54835_MOESM1_ESM.zip › 5000/train/Negative/00584.jpg]

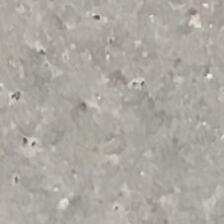

Supplement: Supplementary file 1 — Supplementary Information 1. [file 41598_2024_54835_MOESM1_ESM.zip › 5000/train/Negative/00585.jpg]

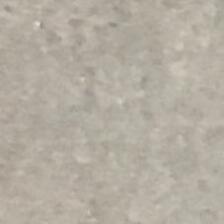

Supplement: Supplementary file 1 — Supplementary Information 1. [file 41598_2024_54835_MOESM1_ESM.zip › 5000/train/Negative/00586.jpg]

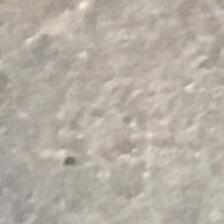

Supplement: Supplementary file 1 — Supplementary Information 1. [file 41598_2024_54835_MOESM1_ESM.zip › 5000/train/Negative/00587.jpg]

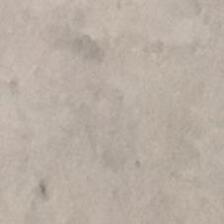

Supplement: Supplementary file 1 — Supplementary Information 1. [file 41598_2024_54835_MOESM1_ESM.zip › 5000/train/Negative/00588.jpg]

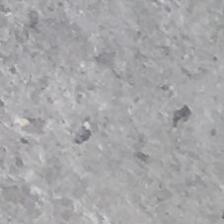

Supplement: Supplementary file 1 — Supplementary Information 1. [file 41598_2024_54835_MOESM1_ESM.zip › 5000/train/Negative/00589.jpg]

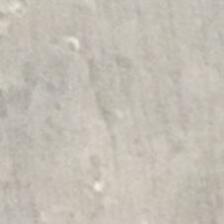

Supplement: Supplementary file 1 — Supplementary Information 1. [file 41598_2024_54835_MOESM1_ESM.zip › 5000/train/Negative/00590.jpg]

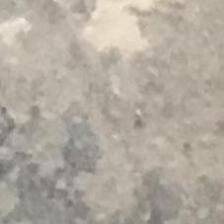

Supplement: Supplementary file 1 — Supplementary Information 1. [file 41598_2024_54835_MOESM1_ESM.zip › 5000/train/Negative/00591.jpg]

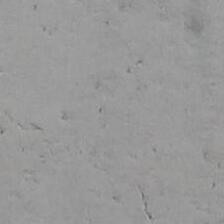

Supplement: Supplementary file 1 — Supplementary Information 1. [file 41598_2024_54835_MOESM1_ESM.zip › 5000/train/Negative/00592.jpg]

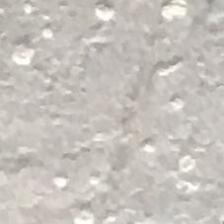

Supplement: Supplementary file 1 — Supplementary Information 1. [file 41598_2024_54835_MOESM1_ESM.zip › 5000/train/Negative/00593.jpg]

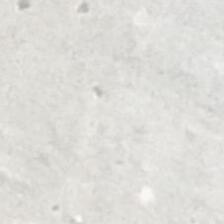

Supplement: Supplementary file 1 — Supplementary Information 1. [file 41598_2024_54835_MOESM1_ESM.zip › 5000/train/Negative/00594.jpg]

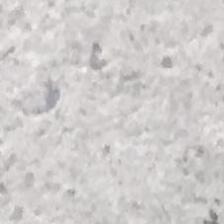

Supplement: Supplementary file 1 — Supplementary Information 1. [file 41598_2024_54835_MOESM1_ESM.zip › 5000/train/Negative/00595.jpg]

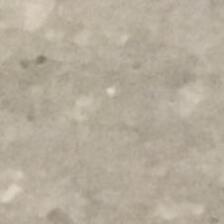

Supplement: Supplementary file 1 — Supplementary Information 1. [file 41598_2024_54835_MOESM1_ESM.zip › 5000/train/Negative/00596.jpg]

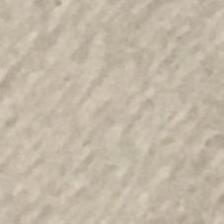

Supplement: Supplementary file 1 — Supplementary Information 1. [file 41598_2024_54835_MOESM1_ESM.zip › 5000/train/Negative/00597.jpg]

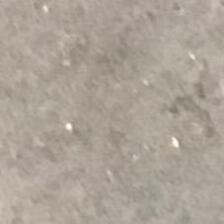

Supplement: Supplementary file 1 — Supplementary Information 1. [file 41598_2024_54835_MOESM1_ESM.zip › 5000/train/Negative/00598.jpg]

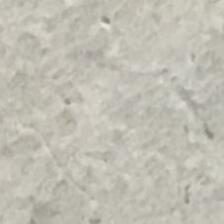

Supplement: Supplementary file 1 — Supplementary Information 1. [file 41598_2024_54835_MOESM1_ESM.zip › 5000/train/Negative/00599.jpg]

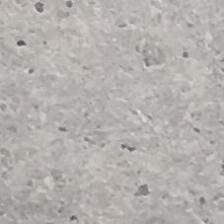

Supplement: Supplementary file 1 — Supplementary Information 1. [file 41598_2024_54835_MOESM1_ESM.zip › 5000/train/Negative/00600.jpg]

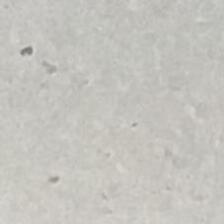

Supplement: Supplementary file 1 — Supplementary Information 1. [file 41598_2024_54835_MOESM1_ESM.zip › 5000/train/Negative/00601.jpg]

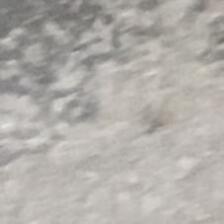

Supplement: Supplementary file 1 — Supplementary Information 1. [file 41598_2024_54835_MOESM1_ESM.zip › 5000/train/Negative/00602.jpg]

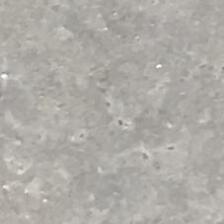

Supplement: Supplementary file 1 — Supplementary Information 1. [file 41598_2024_54835_MOESM1_ESM.zip › 5000/train/Negative/00603.jpg]

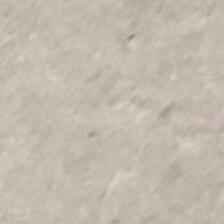

Supplement: Supplementary file 1 — Supplementary Information 1. [file 41598_2024_54835_MOESM1_ESM.zip › 5000/train/Negative/00604.jpg]

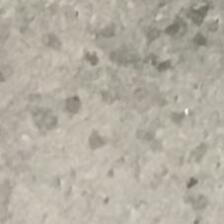

Supplement: Supplementary file 1 — Supplementary Information 1. [file 41598_2024_54835_MOESM1_ESM.zip › 5000/train/Negative/00605.jpg]

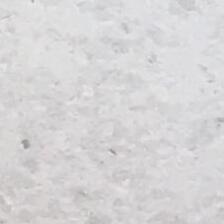

Supplement: Supplementary file 1 — Supplementary Information 1. [file 41598_2024_54835_MOESM1_ESM.zip › 5000/train/Negative/00606.jpg]

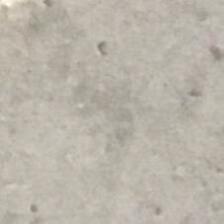

Supplement: Supplementary file 1 — Supplementary Information 1. [file 41598_2024_54835_MOESM1_ESM.zip › 5000/train/Negative/00607.jpg]

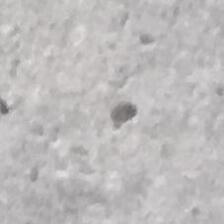

Supplement: Supplementary file 1 — Supplementary Information 1. [file 41598_2024_54835_MOESM1_ESM.zip › 5000/train/Negative/00608.jpg]

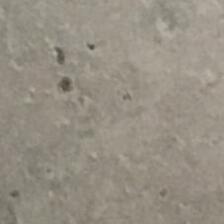

Supplement: Supplementary file 1 — Supplementary Information 1. [file 41598_2024_54835_MOESM1_ESM.zip › 5000/train/Negative/00609.jpg]

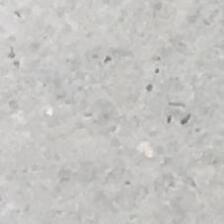

Supplement: Supplementary file 1 — Supplementary Information 1. [file 41598_2024_54835_MOESM1_ESM.zip › 5000/train/Negative/00610.jpg]

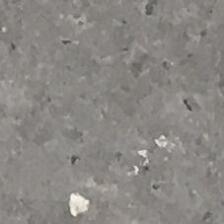

Supplement: Supplementary file 1 — Supplementary Information 1. [file 41598_2024_54835_MOESM1_ESM.zip › 5000/train/Negative/00611.jpg]

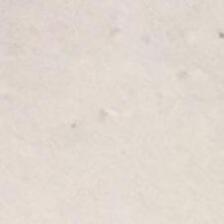

Supplement: Supplementary file 1 — Supplementary Information 1. [file 41598_2024_54835_MOESM1_ESM.zip › 5000/train/Negative/00612.jpg]

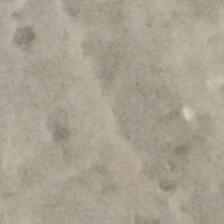

Supplement: Supplementary file 1 — Supplementary Information 1. [file 41598_2024_54835_MOESM1_ESM.zip › 5000/train/Negative/00613.jpg]

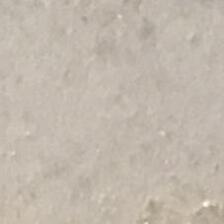

Supplement: Supplementary file 1 — Supplementary Information 1. [file 41598_2024_54835_MOESM1_ESM.zip › 5000/train/Negative/00614.jpg]

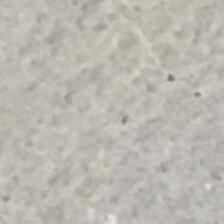

Supplement: Supplementary file 1 — Supplementary Information 1. [file 41598_2024_54835_MOESM1_ESM.zip › 5000/train/Negative/00615.jpg]

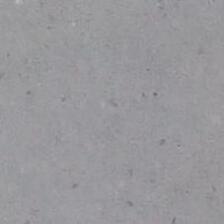

Supplement: Supplementary file 1 — Supplementary Information 1. [file 41598_2024_54835_MOESM1_ESM.zip › 5000/train/Negative/00616.jpg]

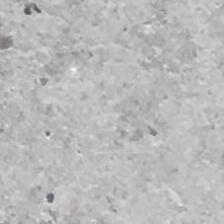

Supplement: Supplementary file 1 — Supplementary Information 1. [file 41598_2024_54835_MOESM1_ESM.zip › 5000/train/Negative/00617.jpg]

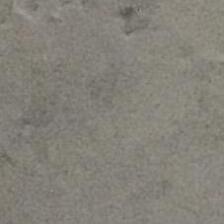

Supplement: Supplementary file 1 — Supplementary Information 1. [file 41598_2024_54835_MOESM1_ESM.zip › 5000/train/Negative/00618.jpg]

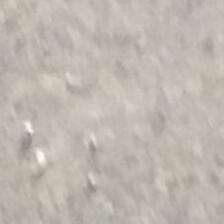

Supplement: Supplementary file 1 — Supplementary Information 1. [file 41598_2024_54835_MOESM1_ESM.zip › 5000/train/Negative/00619.jpg]

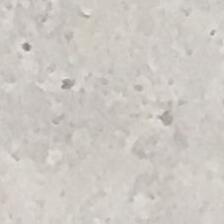

Supplement: Supplementary file 1 — Supplementary Information 1. [file 41598_2024_54835_MOESM1_ESM.zip › 5000/train/Negative/00620.jpg]

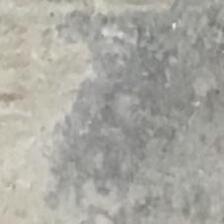

Supplement: Supplementary file 1 — Supplementary Information 1. [file 41598_2024_54835_MOESM1_ESM.zip › 5000/train/Negative/00621.jpg]

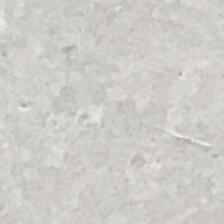

Supplement: Supplementary file 1 — Supplementary Information 1. [file 41598_2024_54835_MOESM1_ESM.zip › 5000/train/Negative/00622.jpg]

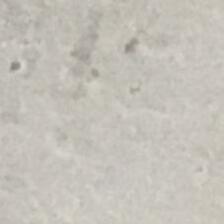

Supplement: Supplementary file 1 — Supplementary Information 1. [file 41598_2024_54835_MOESM1_ESM.zip › 5000/train/Negative/00623.jpg]

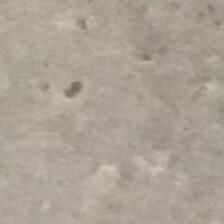

Supplement: Supplementary file 1 — Supplementary Information 1. [file 41598_2024_54835_MOESM1_ESM.zip › 5000/train/Negative/00624.jpg]

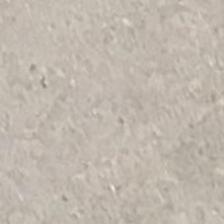

Supplement: Supplementary file 1 — Supplementary Information 1. [file 41598_2024_54835_MOESM1_ESM.zip › 5000/train/Negative/00625.jpg]

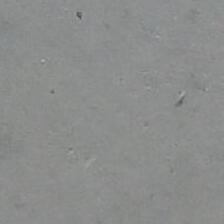

Supplement: Supplementary file 1 — Supplementary Information 1. [file 41598_2024_54835_MOESM1_ESM.zip › 5000/train/Negative/00626.jpg]

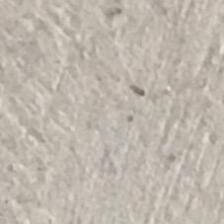

Supplement: Supplementary file 1 — Supplementary Information 1. [file 41598_2024_54835_MOESM1_ESM.zip › 5000/train/Negative/00627.jpg]

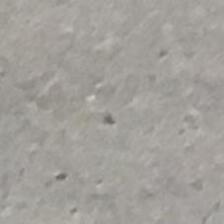

Supplement: Supplementary file 1 — Supplementary Information 1. [file 41598_2024_54835_MOESM1_ESM.zip › 5000/train/Negative/00628.jpg]

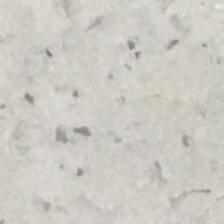

Supplement: Supplementary file 1 — Supplementary Information 1. [file 41598_2024_54835_MOESM1_ESM.zip › 5000/train/Negative/00629.jpg]

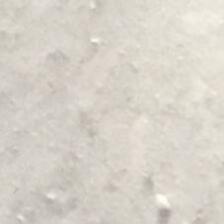

Supplement: Supplementary file 1 — Supplementary Information 1. [file 41598_2024_54835_MOESM1_ESM.zip › 5000/train/Negative/00630.jpg]

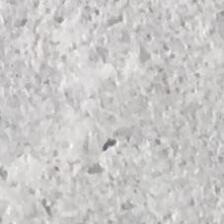

Supplement: Supplementary file 1 — Supplementary Information 1. [file 41598_2024_54835_MOESM1_ESM.zip › 5000/train/Negative/00631.jpg]

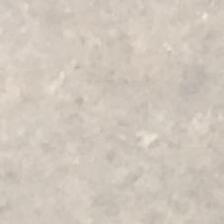

Supplement: Supplementary file 1 — Supplementary Information 1. [file 41598_2024_54835_MOESM1_ESM.zip › 5000/train/Negative/00632.jpg]

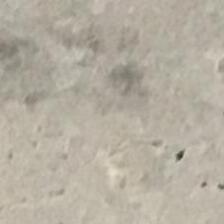

Supplement: Supplementary file 1 — Supplementary Information 1. [file 41598_2024_54835_MOESM1_ESM.zip › 5000/train/Negative/00633.jpg]

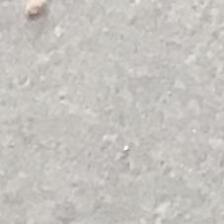

Supplement: Supplementary file 1 — Supplementary Information 1. [file 41598_2024_54835_MOESM1_ESM.zip › 5000/train/Negative/00634.jpg]

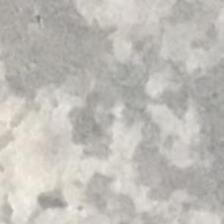

Supplement: Supplementary file 1 — Supplementary Information 1. [file 41598_2024_54835_MOESM1_ESM.zip › 5000/train/Negative/00635.jpg]

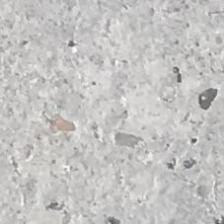

Supplement: Supplementary file 1 — Supplementary Information 1. [file 41598_2024_54835_MOESM1_ESM.zip › 5000/train/Negative/00636.jpg]

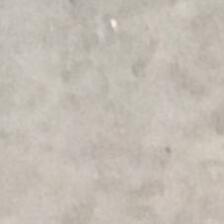

Supplement: Supplementary file 1 — Supplementary Information 1. [file 41598_2024_54835_MOESM1_ESM.zip › 5000/train/Negative/00637.jpg]

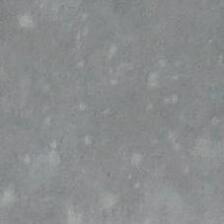

Supplement: Supplementary file 1 — Supplementary Information 1. [file 41598_2024_54835_MOESM1_ESM.zip › 5000/train/Negative/00638.jpg]

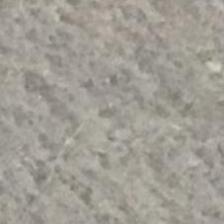

Supplement: Supplementary file 1 — Supplementary Information 1. [file 41598_2024_54835_MOESM1_ESM.zip › 5000/train/Negative/00639.jpg]

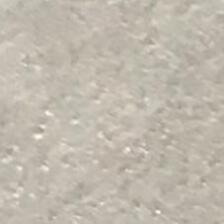

Supplement: Supplementary file 1 — Supplementary Information 1. [file 41598_2024_54835_MOESM1_ESM.zip › 5000/train/Negative/00640.jpg]

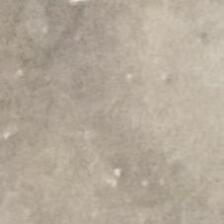

Supplement: Supplementary file 1 — Supplementary Information 1. [file 41598_2024_54835_MOESM1_ESM.zip › 5000/train/Negative/00641.jpg]

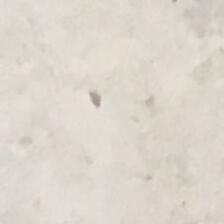

Supplement: Supplementary file 1 — Supplementary Information 1. [file 41598_2024_54835_MOESM1_ESM.zip › 5000/train/Negative/00642.jpg]

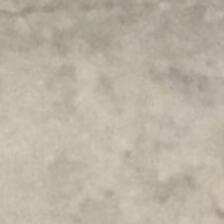

Supplement: Supplementary file 1 — Supplementary Information 1. [file 41598_2024_54835_MOESM1_ESM.zip › 5000/train/Negative/00643.jpg]

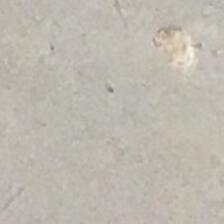

Supplement: Supplementary file 1 — Supplementary Information 1. [file 41598_2024_54835_MOESM1_ESM.zip › 5000/train/Negative/00644.jpg]

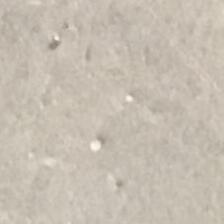

Supplement: Supplementary file 1 — Supplementary Information 1. [file 41598_2024_54835_MOESM1_ESM.zip › 5000/train/Negative/00645.jpg]

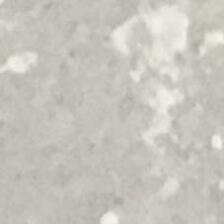

Supplement: Supplementary file 1 — Supplementary Information 1. [file 41598_2024_54835_MOESM1_ESM.zip › 5000/train/Negative/00646.jpg]

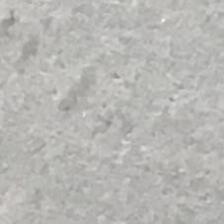

Supplement: Supplementary file 1 — Supplementary Information 1. [file 41598_2024_54835_MOESM1_ESM.zip › 5000/train/Negative/00647.jpg]

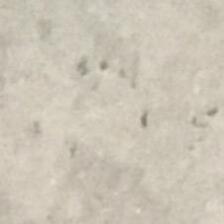

Supplement: Supplementary file 1 — Supplementary Information 1. [file 41598_2024_54835_MOESM1_ESM.zip › 5000/train/Negative/00648.jpg]

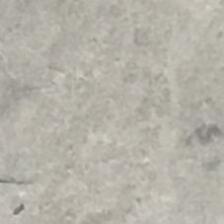

Supplement: Supplementary file 1 — Supplementary Information 1. [file 41598_2024_54835_MOESM1_ESM.zip › 5000/train/Negative/00649.jpg]

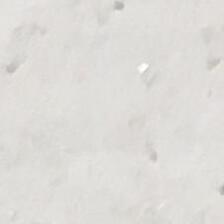

Supplement: Supplementary file 1 — Supplementary Information 1. [file 41598_2024_54835_MOESM1_ESM.zip › 5000/train/Negative/00650.jpg]

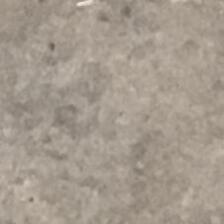

Supplement: Supplementary file 1 — Supplementary Information 1. [file 41598_2024_54835_MOESM1_ESM.zip › 5000/train/Negative/00651.jpg]

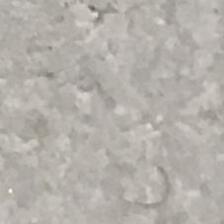

Supplement: Supplementary file 1 — Supplementary Information 1. [file 41598_2024_54835_MOESM1_ESM.zip › 5000/train/Negative/00652.jpg]

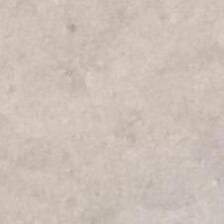

Supplement: Supplementary file 1 — Supplementary Information 1. [file 41598_2024_54835_MOESM1_ESM.zip › 5000/train/Negative/00653.jpg]

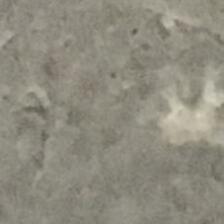

Supplement: Supplementary file 1 — Supplementary Information 1. [file 41598_2024_54835_MOESM1_ESM.zip › 5000/train/Negative/00654.jpg]

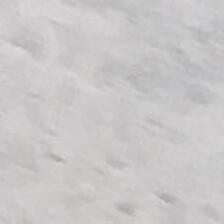

Supplement: Supplementary file 1 — Supplementary Information 1. [file 41598_2024_54835_MOESM1_ESM.zip › 5000/train/Negative/00655.jpg]

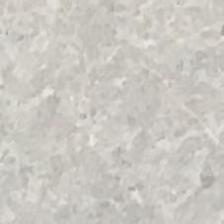

Supplement: Supplementary file 1 — Supplementary Information 1. [file 41598_2024_54835_MOESM1_ESM.zip › 5000/train/Negative/00656.jpg]

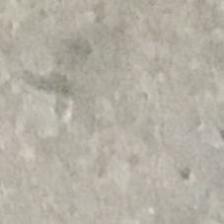

Supplement: Supplementary file 1 — Supplementary Information 1. [file 41598_2024_54835_MOESM1_ESM.zip › 5000/train/Negative/00657.jpg]

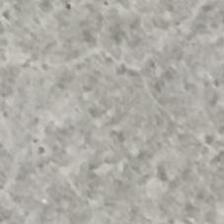

Supplement: Supplementary file 1 — Supplementary Information 1. [file 41598_2024_54835_MOESM1_ESM.zip › 5000/train/Negative/00658.jpg]

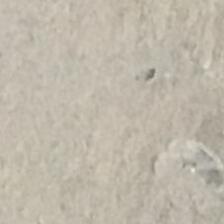

Supplement: Supplementary file 1 — Supplementary Information 1. [file 41598_2024_54835_MOESM1_ESM.zip › 5000/train/Negative/00659.jpg]

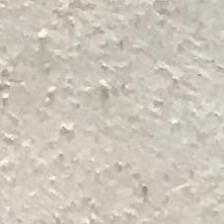

Supplement: Supplementary file 1 — Supplementary Information 1. [file 41598_2024_54835_MOESM1_ESM.zip › 5000/train/Negative/00660.jpg]
