# Supplementary figures and images for: An improved transformer-based concrete crack classification method (part 1 of 7)
Source: Sci Rep. 2024 Mar 14;14:6226. doi: 10.1038/s41598-024-54835-x (PMC10940720; doi:10.1038/s41598-024-54835-x)

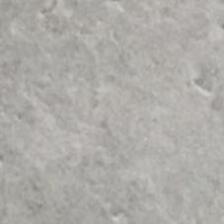

Supplement: Supplementary file 1 — Supplementary Information 1. [file 41598_2024_54835_MOESM1_ESM.zip › 5000/train/Negative/00461.jpg]

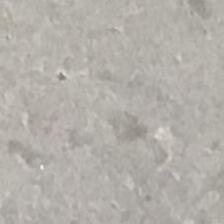

Supplement: Supplementary file 1 — Supplementary Information 1. [file 41598_2024_54835_MOESM1_ESM.zip › 5000/train/Negative/00462.jpg]

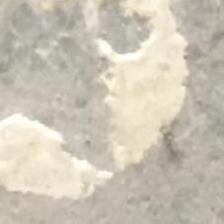

Supplement: Supplementary file 1 — Supplementary Information 1. [file 41598_2024_54835_MOESM1_ESM.zip › 5000/train/Negative/00463.jpg]

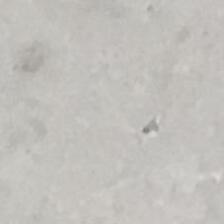

Supplement: Supplementary file 1 — Supplementary Information 1. [file 41598_2024_54835_MOESM1_ESM.zip › 5000/train/Negative/00464.jpg]

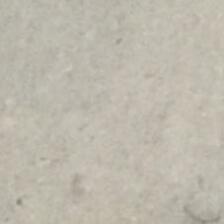

Supplement: Supplementary file 1 — Supplementary Information 1. [file 41598_2024_54835_MOESM1_ESM.zip › 5000/train/Negative/00465.jpg]

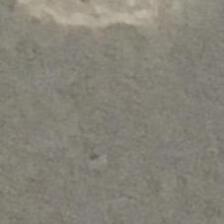

Supplement: Supplementary file 1 — Supplementary Information 1. [file 41598_2024_54835_MOESM1_ESM.zip › 5000/train/Negative/00466.jpg]

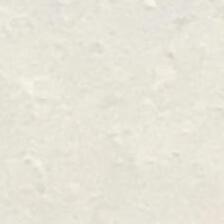

Supplement: Supplementary file 1 — Supplementary Information 1. [file 41598_2024_54835_MOESM1_ESM.zip › 5000/train/Negative/00467.jpg]

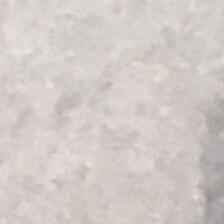

Supplement: Supplementary file 1 — Supplementary Information 1. [file 41598_2024_54835_MOESM1_ESM.zip › 5000/train/Negative/00468.jpg]

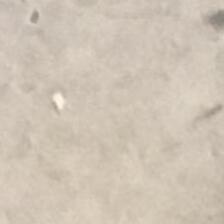

Supplement: Supplementary file 1 — Supplementary Information 1. [file 41598_2024_54835_MOESM1_ESM.zip › 5000/train/Negative/00469.jpg]

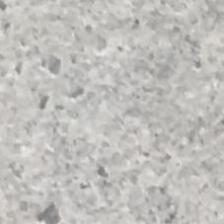

Supplement: Supplementary file 1 — Supplementary Information 1. [file 41598_2024_54835_MOESM1_ESM.zip › 5000/train/Negative/00470.jpg]

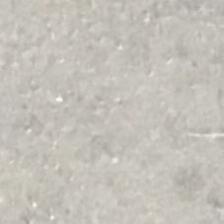

Supplement: Supplementary file 1 — Supplementary Information 1. [file 41598_2024_54835_MOESM1_ESM.zip › 5000/train/Negative/00471.jpg]

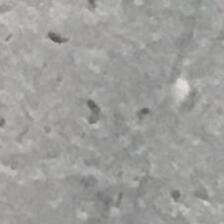

Supplement: Supplementary file 1 — Supplementary Information 1. [file 41598_2024_54835_MOESM1_ESM.zip › 5000/train/Negative/00472.jpg]

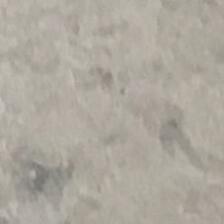

Supplement: Supplementary file 1 — Supplementary Information 1. [file 41598_2024_54835_MOESM1_ESM.zip › 5000/train/Negative/00473.jpg]

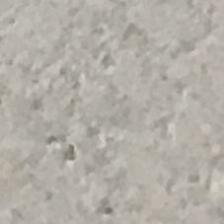

Supplement: Supplementary file 1 — Supplementary Information 1. [file 41598_2024_54835_MOESM1_ESM.zip › 5000/train/Negative/00474.jpg]

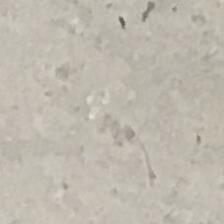

Supplement: Supplementary file 1 — Supplementary Information 1. [file 41598_2024_54835_MOESM1_ESM.zip › 5000/train/Negative/00475.jpg]

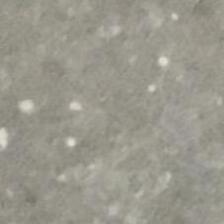

Supplement: Supplementary file 1 — Supplementary Information 1. [file 41598_2024_54835_MOESM1_ESM.zip › 5000/train/Negative/00476.jpg]

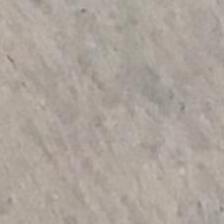

Supplement: Supplementary file 1 — Supplementary Information 1. [file 41598_2024_54835_MOESM1_ESM.zip › 5000/train/Negative/00477.jpg]

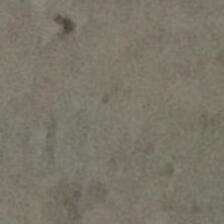

Supplement: Supplementary file 1 — Supplementary Information 1. [file 41598_2024_54835_MOESM1_ESM.zip › 5000/train/Negative/00478.jpg]

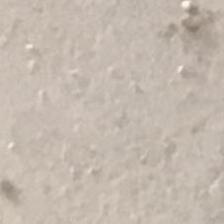

Supplement: Supplementary file 1 — Supplementary Information 1. [file 41598_2024_54835_MOESM1_ESM.zip › 5000/train/Negative/00479.jpg]

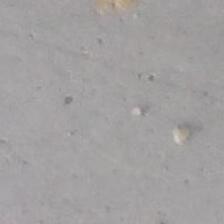

Supplement: Supplementary file 1 — Supplementary Information 1. [file 41598_2024_54835_MOESM1_ESM.zip › 5000/train/Negative/00480.jpg]

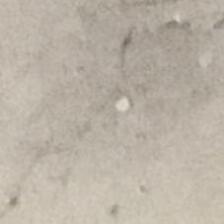

Supplement: Supplementary file 1 — Supplementary Information 1. [file 41598_2024_54835_MOESM1_ESM.zip › 5000/train/Negative/00481.jpg]

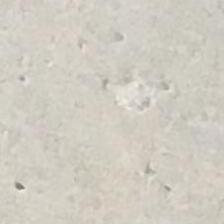

Supplement: Supplementary file 1 — Supplementary Information 1. [file 41598_2024_54835_MOESM1_ESM.zip › 5000/train/Negative/00482.jpg]

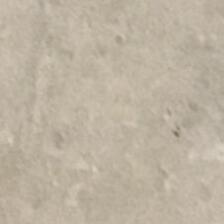

Supplement: Supplementary file 1 — Supplementary Information 1. [file 41598_2024_54835_MOESM1_ESM.zip › 5000/train/Negative/00483.jpg]

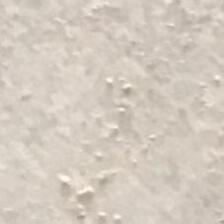

Supplement: Supplementary file 1 — Supplementary Information 1. [file 41598_2024_54835_MOESM1_ESM.zip › 5000/train/Negative/00484.jpg]

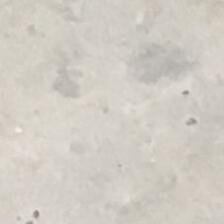

Supplement: Supplementary file 1 — Supplementary Information 1. [file 41598_2024_54835_MOESM1_ESM.zip › 5000/train/Negative/00485.jpg]

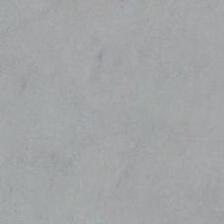

Supplement: Supplementary file 1 — Supplementary Information 1. [file 41598_2024_54835_MOESM1_ESM.zip › 5000/train/Negative/00486.jpg]

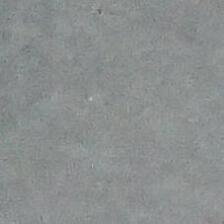

Supplement: Supplementary file 1 — Supplementary Information 1. [file 41598_2024_54835_MOESM1_ESM.zip › 5000/train/Negative/00487.jpg]

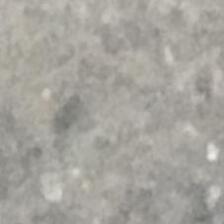

Supplement: Supplementary file 1 — Supplementary Information 1. [file 41598_2024_54835_MOESM1_ESM.zip › 5000/train/Negative/00488.jpg]

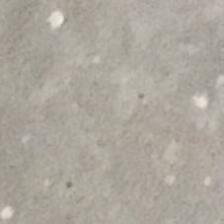

Supplement: Supplementary file 1 — Supplementary Information 1. [file 41598_2024_54835_MOESM1_ESM.zip › 5000/train/Negative/00489.jpg]

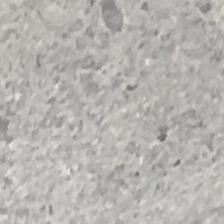

Supplement: Supplementary file 1 — Supplementary Information 1. [file 41598_2024_54835_MOESM1_ESM.zip › 5000/train/Negative/00490.jpg]

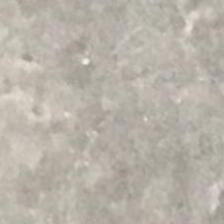

Supplement: Supplementary file 1 — Supplementary Information 1. [file 41598_2024_54835_MOESM1_ESM.zip › 5000/train/Negative/00491.jpg]

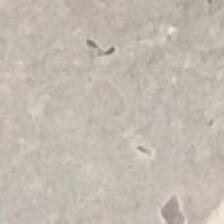

Supplement: Supplementary file 1 — Supplementary Information 1. [file 41598_2024_54835_MOESM1_ESM.zip › 5000/train/Negative/00492.jpg]

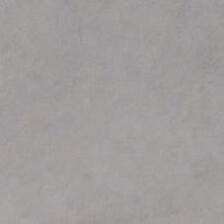

Supplement: Supplementary file 1 — Supplementary Information 1. [file 41598_2024_54835_MOESM1_ESM.zip › 5000/train/Negative/00493.jpg]

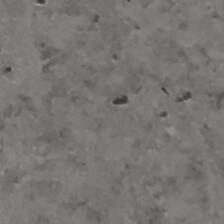

Supplement: Supplementary file 1 — Supplementary Information 1. [file 41598_2024_54835_MOESM1_ESM.zip › 5000/train/Negative/00494.jpg]

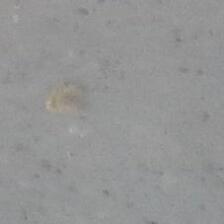

Supplement: Supplementary file 1 — Supplementary Information 1. [file 41598_2024_54835_MOESM1_ESM.zip › 5000/train/Negative/00495.jpg]

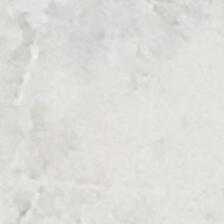

Supplement: Supplementary file 1 — Supplementary Information 1. [file 41598_2024_54835_MOESM1_ESM.zip › 5000/train/Negative/00496.jpg]

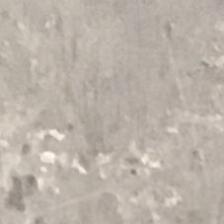

Supplement: Supplementary file 1 — Supplementary Information 1. [file 41598_2024_54835_MOESM1_ESM.zip › 5000/train/Negative/00497.jpg]

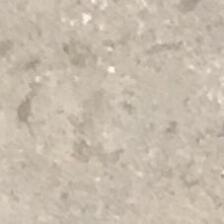

Supplement: Supplementary file 1 — Supplementary Information 1. [file 41598_2024_54835_MOESM1_ESM.zip › 5000/train/Negative/00498.jpg]

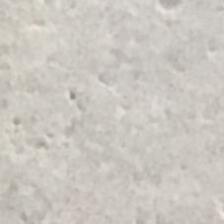

Supplement: Supplementary file 1 — Supplementary Information 1. [file 41598_2024_54835_MOESM1_ESM.zip › 5000/train/Negative/00499.jpg]

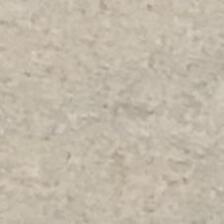

Supplement: Supplementary file 1 — Supplementary Information 1. [file 41598_2024_54835_MOESM1_ESM.zip › 5000/train/Negative/00500.jpg]

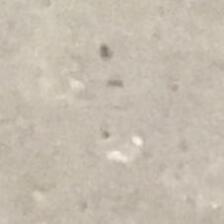

Supplement: Supplementary file 1 — Supplementary Information 1. [file 41598_2024_54835_MOESM1_ESM.zip › 5000/train/Negative/00501.jpg]

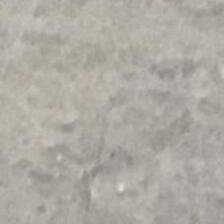

Supplement: Supplementary file 1 — Supplementary Information 1. [file 41598_2024_54835_MOESM1_ESM.zip › 5000/train/Negative/00502.jpg]

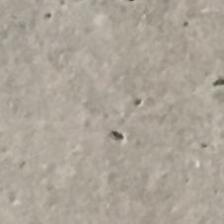

Supplement: Supplementary file 1 — Supplementary Information 1. [file 41598_2024_54835_MOESM1_ESM.zip › 5000/train/Negative/00503.jpg]

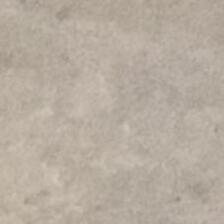

Supplement: Supplementary file 1 — Supplementary Information 1. [file 41598_2024_54835_MOESM1_ESM.zip › 5000/train/Negative/00504.jpg]

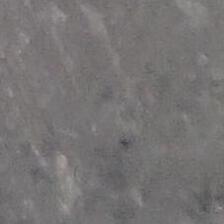

Supplement: Supplementary file 1 — Supplementary Information 1. [file 41598_2024_54835_MOESM1_ESM.zip › 5000/train/Negative/00505.jpg]

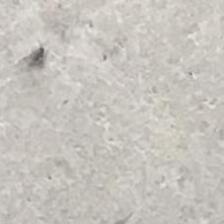

Supplement: Supplementary file 1 — Supplementary Information 1. [file 41598_2024_54835_MOESM1_ESM.zip › 5000/train/Negative/00506.jpg]

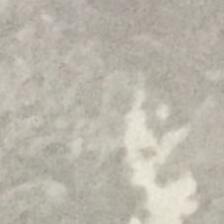

Supplement: Supplementary file 1 — Supplementary Information 1. [file 41598_2024_54835_MOESM1_ESM.zip › 5000/train/Negative/00507.jpg]

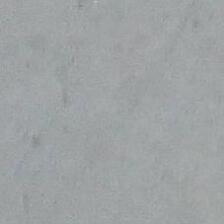

Supplement: Supplementary file 1 — Supplementary Information 1. [file 41598_2024_54835_MOESM1_ESM.zip › 5000/train/Negative/00508.jpg]

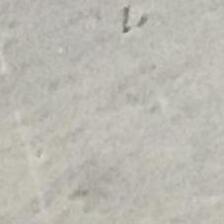

Supplement: Supplementary file 1 — Supplementary Information 1. [file 41598_2024_54835_MOESM1_ESM.zip › 5000/train/Negative/00509.jpg]

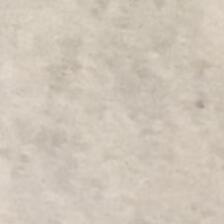

Supplement: Supplementary file 1 — Supplementary Information 1. [file 41598_2024_54835_MOESM1_ESM.zip › 5000/train/Negative/00510.jpg]

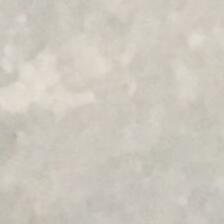

Supplement: Supplementary file 1 — Supplementary Information 1. [file 41598_2024_54835_MOESM1_ESM.zip › 5000/train/Negative/00511.jpg]

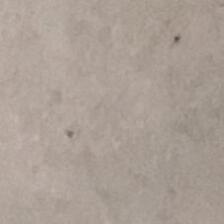

Supplement: Supplementary file 1 — Supplementary Information 1. [file 41598_2024_54835_MOESM1_ESM.zip › 5000/train/Negative/00512.jpg]

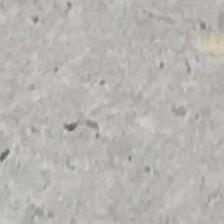

Supplement: Supplementary file 1 — Supplementary Information 1. [file 41598_2024_54835_MOESM1_ESM.zip › 5000/train/Negative/00513.jpg]

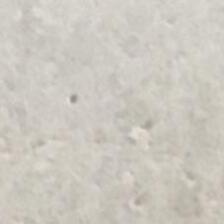

Supplement: Supplementary file 1 — Supplementary Information 1. [file 41598_2024_54835_MOESM1_ESM.zip › 5000/train/Negative/00514.jpg]

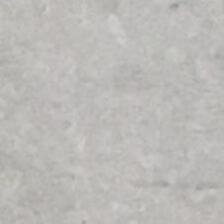

Supplement: Supplementary file 1 — Supplementary Information 1. [file 41598_2024_54835_MOESM1_ESM.zip › 5000/train/Negative/00515.jpg]

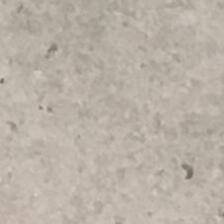

Supplement: Supplementary file 1 — Supplementary Information 1. [file 41598_2024_54835_MOESM1_ESM.zip › 5000/train/Negative/00516.jpg]

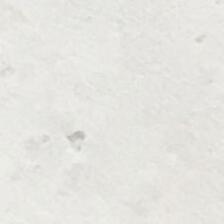

Supplement: Supplementary file 1 — Supplementary Information 1. [file 41598_2024_54835_MOESM1_ESM.zip › 5000/train/Negative/00517.jpg]

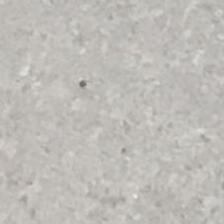

Supplement: Supplementary file 1 — Supplementary Information 1. [file 41598_2024_54835_MOESM1_ESM.zip › 5000/train/Negative/00518.jpg]

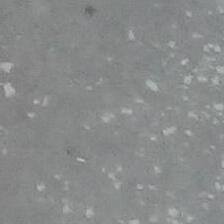

Supplement: Supplementary file 1 — Supplementary Information 1. [file 41598_2024_54835_MOESM1_ESM.zip › 5000/train/Negative/00519.jpg]

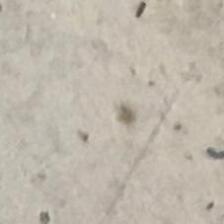

Supplement: Supplementary file 1 — Supplementary Information 1. [file 41598_2024_54835_MOESM1_ESM.zip › 5000/train/Negative/00520.jpg]

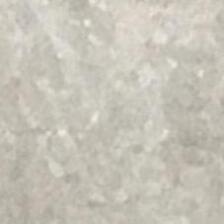

Supplement: Supplementary file 1 — Supplementary Information 1. [file 41598_2024_54835_MOESM1_ESM.zip › 5000/train/Negative/00521.jpg]

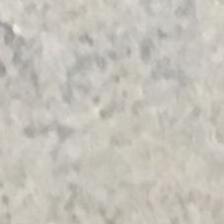

Supplement: Supplementary file 1 — Supplementary Information 1. [file 41598_2024_54835_MOESM1_ESM.zip › 5000/train/Negative/00522.jpg]

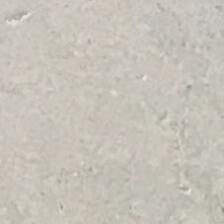

Supplement: Supplementary file 1 — Supplementary Information 1. [file 41598_2024_54835_MOESM1_ESM.zip › 5000/train/Negative/00523.jpg]

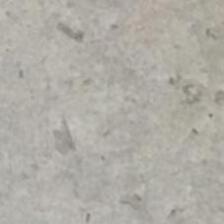

Supplement: Supplementary file 1 — Supplementary Information 1. [file 41598_2024_54835_MOESM1_ESM.zip › 5000/train/Negative/00524.jpg]

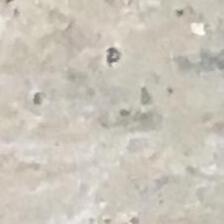

Supplement: Supplementary file 1 — Supplementary Information 1. [file 41598_2024_54835_MOESM1_ESM.zip › 5000/train/Negative/00525.jpg]

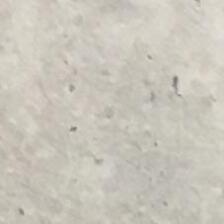

Supplement: Supplementary file 1 — Supplementary Information 1. [file 41598_2024_54835_MOESM1_ESM.zip › 5000/train/Negative/00526.jpg]

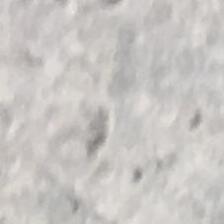

Supplement: Supplementary file 1 — Supplementary Information 1. [file 41598_2024_54835_MOESM1_ESM.zip › 5000/train/Negative/00527.jpg]

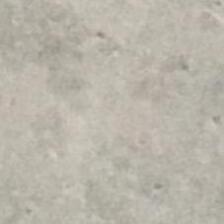

Supplement: Supplementary file 1 — Supplementary Information 1. [file 41598_2024_54835_MOESM1_ESM.zip › 5000/train/Negative/00528.jpg]

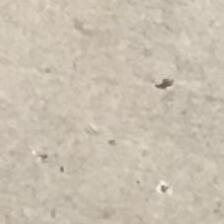

Supplement: Supplementary file 1 — Supplementary Information 1. [file 41598_2024_54835_MOESM1_ESM.zip › 5000/train/Negative/00529.jpg]

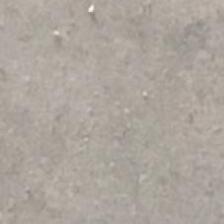

Supplement: Supplementary file 1 — Supplementary Information 1. [file 41598_2024_54835_MOESM1_ESM.zip › 5000/train/Negative/00530.jpg]

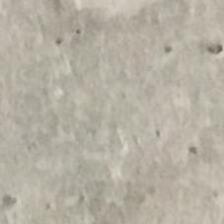

Supplement: Supplementary file 1 — Supplementary Information 1. [file 41598_2024_54835_MOESM1_ESM.zip › 5000/train/Negative/00531.jpg]

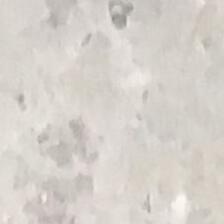

Supplement: Supplementary file 1 — Supplementary Information 1. [file 41598_2024_54835_MOESM1_ESM.zip › 5000/train/Negative/00532.jpg]

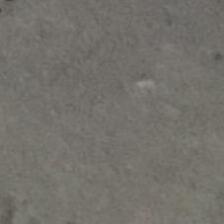

Supplement: Supplementary file 1 — Supplementary Information 1. [file 41598_2024_54835_MOESM1_ESM.zip › 5000/train/Negative/00533.jpg]

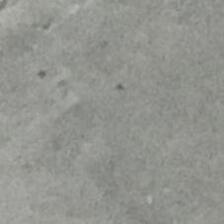

Supplement: Supplementary file 1 — Supplementary Information 1. [file 41598_2024_54835_MOESM1_ESM.zip › 5000/train/Negative/00534.jpg]

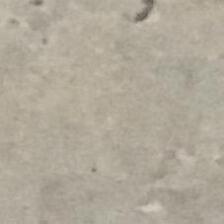

Supplement: Supplementary file 1 — Supplementary Information 1. [file 41598_2024_54835_MOESM1_ESM.zip › 5000/train/Negative/00535.jpg]

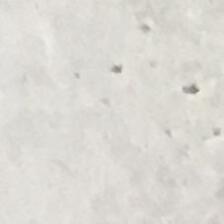

Supplement: Supplementary file 1 — Supplementary Information 1. [file 41598_2024_54835_MOESM1_ESM.zip › 5000/train/Negative/00536.jpg]

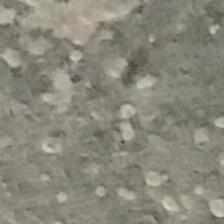

Supplement: Supplementary file 1 — Supplementary Information 1. [file 41598_2024_54835_MOESM1_ESM.zip › 5000/train/Negative/00537.jpg]

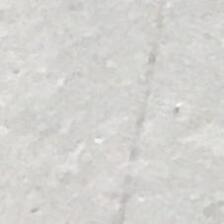

Supplement: Supplementary file 1 — Supplementary Information 1. [file 41598_2024_54835_MOESM1_ESM.zip › 5000/train/Negative/00538.jpg]

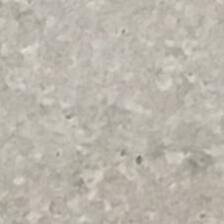

Supplement: Supplementary file 1 — Supplementary Information 1. [file 41598_2024_54835_MOESM1_ESM.zip › 5000/train/Negative/00539.jpg]

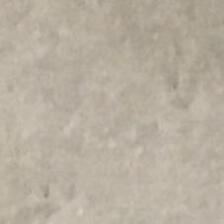

Supplement: Supplementary file 1 — Supplementary Information 1. [file 41598_2024_54835_MOESM1_ESM.zip › 5000/train/Negative/00540.jpg]

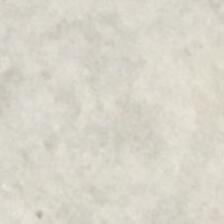

Supplement: Supplementary file 1 — Supplementary Information 1. [file 41598_2024_54835_MOESM1_ESM.zip › 5000/train/Negative/00541.jpg]

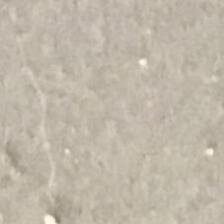

Supplement: Supplementary file 1 — Supplementary Information 1. [file 41598_2024_54835_MOESM1_ESM.zip › 5000/train/Negative/00542.jpg]

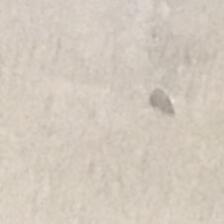

Supplement: Supplementary file 1 — Supplementary Information 1. [file 41598_2024_54835_MOESM1_ESM.zip › 5000/train/Negative/00543.jpg]

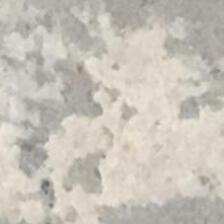

Supplement: Supplementary file 1 — Supplementary Information 1. [file 41598_2024_54835_MOESM1_ESM.zip › 5000/train/Negative/00544.jpg]

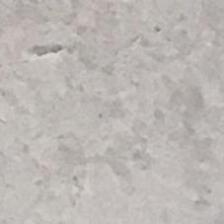

Supplement: Supplementary file 1 — Supplementary Information 1. [file 41598_2024_54835_MOESM1_ESM.zip › 5000/train/Negative/00545.jpg]

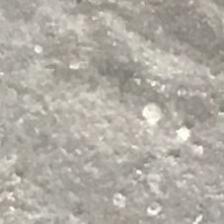

Supplement: Supplementary file 1 — Supplementary Information 1. [file 41598_2024_54835_MOESM1_ESM.zip › 5000/train/Negative/00546.jpg]

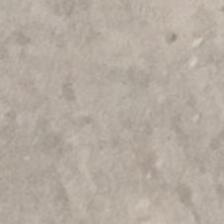

Supplement: Supplementary file 1 — Supplementary Information 1. [file 41598_2024_54835_MOESM1_ESM.zip › 5000/train/Negative/00547.jpg]

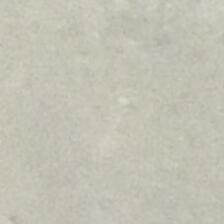

Supplement: Supplementary file 1 — Supplementary Information 1. [file 41598_2024_54835_MOESM1_ESM.zip › 5000/train/Negative/00548.jpg]

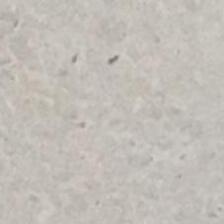

Supplement: Supplementary file 1 — Supplementary Information 1. [file 41598_2024_54835_MOESM1_ESM.zip › 5000/train/Negative/00549.jpg]

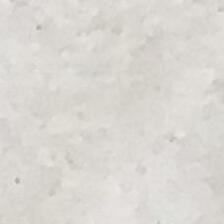

Supplement: Supplementary file 1 — Supplementary Information 1. [file 41598_2024_54835_MOESM1_ESM.zip › 5000/train/Negative/00550.jpg]

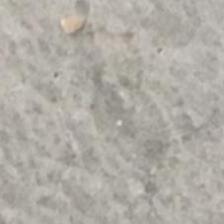

Supplement: Supplementary file 1 — Supplementary Information 1. [file 41598_2024_54835_MOESM1_ESM.zip › 5000/train/Negative/00551.jpg]

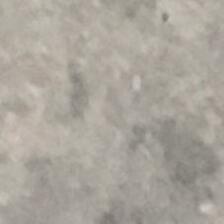

Supplement: Supplementary file 1 — Supplementary Information 1. [file 41598_2024_54835_MOESM1_ESM.zip › 5000/train/Negative/00552.jpg]

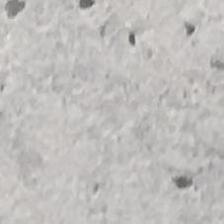

Supplement: Supplementary file 1 — Supplementary Information 1. [file 41598_2024_54835_MOESM1_ESM.zip › 5000/train/Negative/00553.jpg]

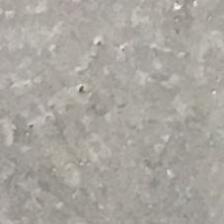

Supplement: Supplementary file 1 — Supplementary Information 1. [file 41598_2024_54835_MOESM1_ESM.zip › 5000/train/Negative/00554.jpg]

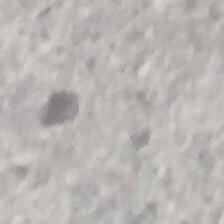

Supplement: Supplementary file 1 — Supplementary Information 1. [file 41598_2024_54835_MOESM1_ESM.zip › 5000/train/Negative/00555.jpg]

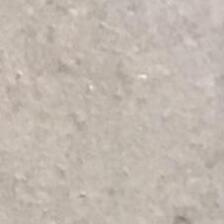

Supplement: Supplementary file 1 — Supplementary Information 1. [file 41598_2024_54835_MOESM1_ESM.zip › 5000/train/Negative/00556.jpg]

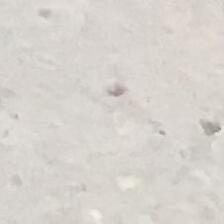

Supplement: Supplementary file 1 — Supplementary Information 1. [file 41598_2024_54835_MOESM1_ESM.zip › 5000/train/Negative/00557.jpg]

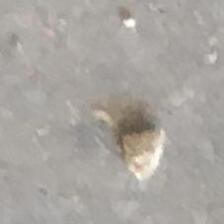

Supplement: Supplementary file 1 — Supplementary Information 1. [file 41598_2024_54835_MOESM1_ESM.zip › 5000/train/Negative/00558.jpg]

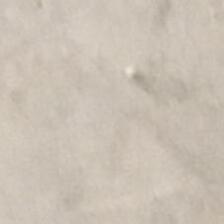

Supplement: Supplementary file 1 — Supplementary Information 1. [file 41598_2024_54835_MOESM1_ESM.zip › 5000/train/Negative/00559.jpg]

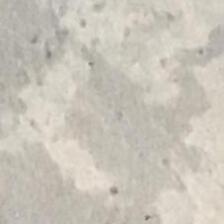

Supplement: Supplementary file 1 — Supplementary Information 1. [file 41598_2024_54835_MOESM1_ESM.zip › 5000/train/Negative/00560.jpg]
