# Supplementary figures and images for: An improved transformer-based concrete crack classification method (part 3 of 7)
Source: Sci Rep. 2024 Mar 14;14:6226. doi: 10.1038/s41598-024-54835-x (PMC10940720; doi:10.1038/s41598-024-54835-x)

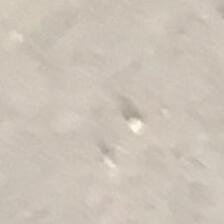

Supplement: Supplementary file 2 — Supplementary Information 2. [file 41598_2024_54835_MOESM2_ESM.zip › 10000/train/Negative/01601.jpg]

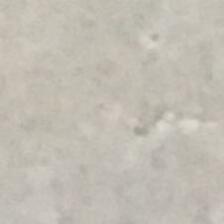

Supplement: Supplementary file 2 — Supplementary Information 2. [file 41598_2024_54835_MOESM2_ESM.zip › 10000/train/Negative/01602.jpg]

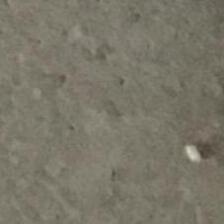

Supplement: Supplementary file 2 — Supplementary Information 2. [file 41598_2024_54835_MOESM2_ESM.zip › 10000/train/Negative/01603.jpg]

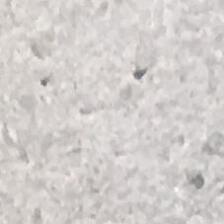

Supplement: Supplementary file 2 — Supplementary Information 2. [file 41598_2024_54835_MOESM2_ESM.zip › 10000/train/Negative/01604.jpg]

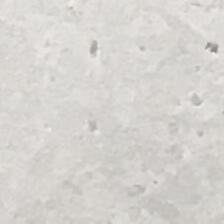

Supplement: Supplementary file 2 — Supplementary Information 2. [file 41598_2024_54835_MOESM2_ESM.zip › 10000/train/Negative/01605.jpg]

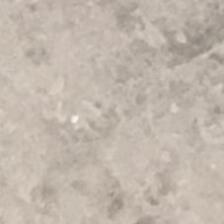

Supplement: Supplementary file 2 — Supplementary Information 2. [file 41598_2024_54835_MOESM2_ESM.zip › 10000/train/Negative/01606.jpg]

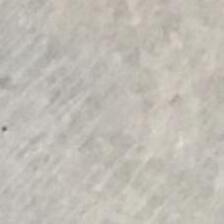

Supplement: Supplementary file 2 — Supplementary Information 2. [file 41598_2024_54835_MOESM2_ESM.zip › 10000/train/Negative/01607.jpg]

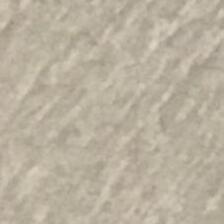

Supplement: Supplementary file 2 — Supplementary Information 2. [file 41598_2024_54835_MOESM2_ESM.zip › 10000/train/Negative/01608.jpg]

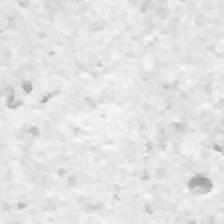

Supplement: Supplementary file 2 — Supplementary Information 2. [file 41598_2024_54835_MOESM2_ESM.zip › 10000/train/Negative/01609.jpg]

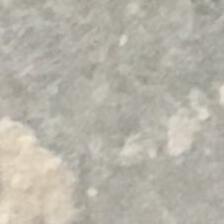

Supplement: Supplementary file 2 — Supplementary Information 2. [file 41598_2024_54835_MOESM2_ESM.zip › 10000/train/Negative/01610.jpg]

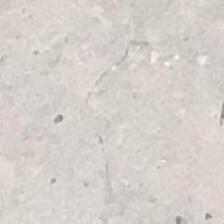

Supplement: Supplementary file 2 — Supplementary Information 2. [file 41598_2024_54835_MOESM2_ESM.zip › 10000/train/Negative/01611.jpg]

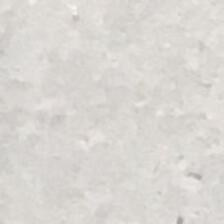

Supplement: Supplementary file 2 — Supplementary Information 2. [file 41598_2024_54835_MOESM2_ESM.zip › 10000/train/Negative/01612.jpg]

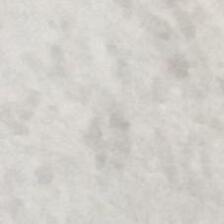

Supplement: Supplementary file 2 — Supplementary Information 2. [file 41598_2024_54835_MOESM2_ESM.zip › 10000/train/Negative/01613.jpg]

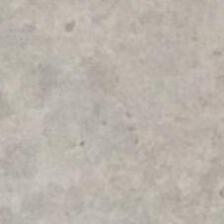

Supplement: Supplementary file 2 — Supplementary Information 2. [file 41598_2024_54835_MOESM2_ESM.zip › 10000/train/Negative/01614.jpg]

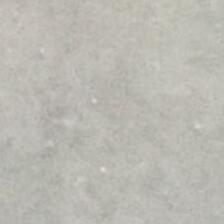

Supplement: Supplementary file 2 — Supplementary Information 2. [file 41598_2024_54835_MOESM2_ESM.zip › 10000/train/Negative/01615.jpg]

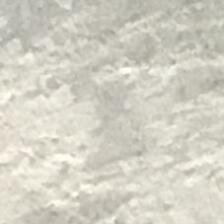

Supplement: Supplementary file 2 — Supplementary Information 2. [file 41598_2024_54835_MOESM2_ESM.zip › 10000/train/Negative/01616.jpg]

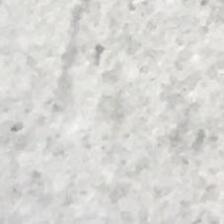

Supplement: Supplementary file 2 — Supplementary Information 2. [file 41598_2024_54835_MOESM2_ESM.zip › 10000/train/Negative/01617.jpg]

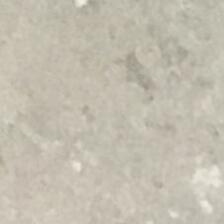

Supplement: Supplementary file 2 — Supplementary Information 2. [file 41598_2024_54835_MOESM2_ESM.zip › 10000/train/Negative/01618.jpg]

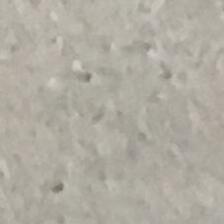

Supplement: Supplementary file 2 — Supplementary Information 2. [file 41598_2024_54835_MOESM2_ESM.zip › 10000/train/Negative/01619.jpg]

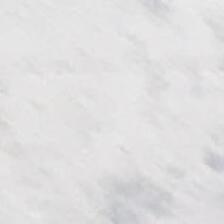

Supplement: Supplementary file 2 — Supplementary Information 2. [file 41598_2024_54835_MOESM2_ESM.zip › 10000/train/Negative/01620.jpg]

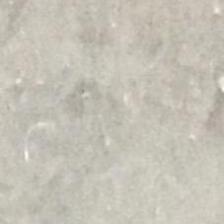

Supplement: Supplementary file 2 — Supplementary Information 2. [file 41598_2024_54835_MOESM2_ESM.zip › 10000/train/Negative/01621.jpg]

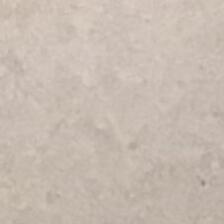

Supplement: Supplementary file 2 — Supplementary Information 2. [file 41598_2024_54835_MOESM2_ESM.zip › 10000/train/Negative/01622.jpg]

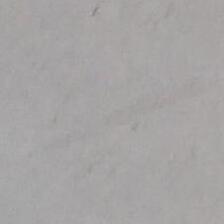

Supplement: Supplementary file 2 — Supplementary Information 2. [file 41598_2024_54835_MOESM2_ESM.zip › 10000/train/Negative/01623.jpg]

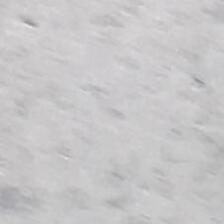

Supplement: Supplementary file 2 — Supplementary Information 2. [file 41598_2024_54835_MOESM2_ESM.zip › 10000/train/Negative/01624.jpg]

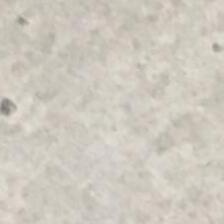

Supplement: Supplementary file 2 — Supplementary Information 2. [file 41598_2024_54835_MOESM2_ESM.zip › 10000/train/Negative/01625.jpg]

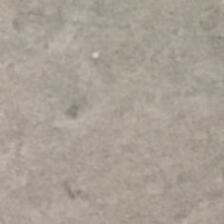

Supplement: Supplementary file 2 — Supplementary Information 2. [file 41598_2024_54835_MOESM2_ESM.zip › 10000/train/Negative/01626.jpg]

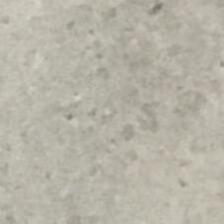

Supplement: Supplementary file 2 — Supplementary Information 2. [file 41598_2024_54835_MOESM2_ESM.zip › 10000/train/Negative/01627.jpg]

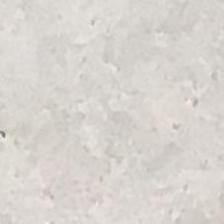

Supplement: Supplementary file 2 — Supplementary Information 2. [file 41598_2024_54835_MOESM2_ESM.zip › 10000/train/Negative/01628.jpg]

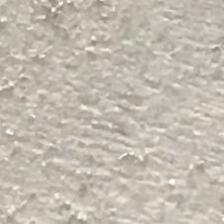

Supplement: Supplementary file 2 — Supplementary Information 2. [file 41598_2024_54835_MOESM2_ESM.zip › 10000/train/Negative/01629.jpg]

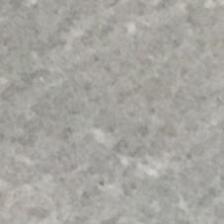

Supplement: Supplementary file 2 — Supplementary Information 2. [file 41598_2024_54835_MOESM2_ESM.zip › 10000/train/Negative/01630.jpg]

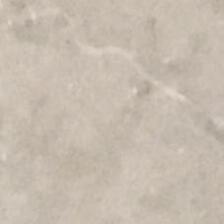

Supplement: Supplementary file 2 — Supplementary Information 2. [file 41598_2024_54835_MOESM2_ESM.zip › 10000/train/Negative/01631.jpg]

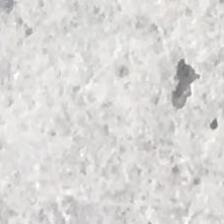

Supplement: Supplementary file 2 — Supplementary Information 2. [file 41598_2024_54835_MOESM2_ESM.zip › 10000/train/Negative/01632.jpg]

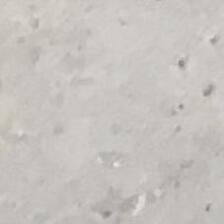

Supplement: Supplementary file 2 — Supplementary Information 2. [file 41598_2024_54835_MOESM2_ESM.zip › 10000/train/Negative/01633.jpg]

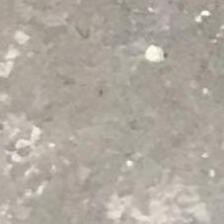

Supplement: Supplementary file 2 — Supplementary Information 2. [file 41598_2024_54835_MOESM2_ESM.zip › 10000/train/Negative/01634.jpg]

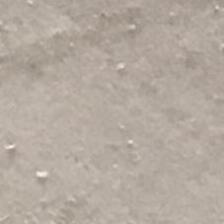

Supplement: Supplementary file 2 — Supplementary Information 2. [file 41598_2024_54835_MOESM2_ESM.zip › 10000/train/Negative/01635.jpg]

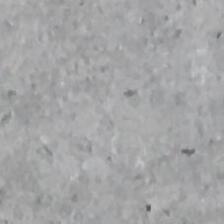

Supplement: Supplementary file 2 — Supplementary Information 2. [file 41598_2024_54835_MOESM2_ESM.zip › 10000/train/Negative/01636.jpg]

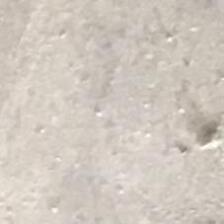

Supplement: Supplementary file 2 — Supplementary Information 2. [file 41598_2024_54835_MOESM2_ESM.zip › 10000/train/Negative/01637.jpg]

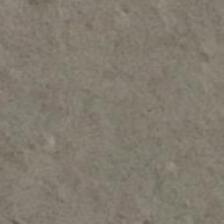

Supplement: Supplementary file 2 — Supplementary Information 2. [file 41598_2024_54835_MOESM2_ESM.zip › 10000/train/Negative/01638.jpg]

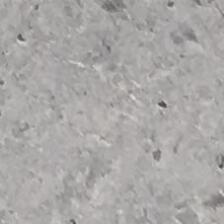

Supplement: Supplementary file 2 — Supplementary Information 2. [file 41598_2024_54835_MOESM2_ESM.zip › 10000/train/Negative/01639.jpg]

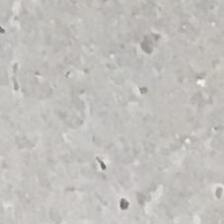

Supplement: Supplementary file 2 — Supplementary Information 2. [file 41598_2024_54835_MOESM2_ESM.zip › 10000/train/Negative/01640.jpg]

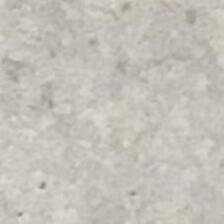

Supplement: Supplementary file 2 — Supplementary Information 2. [file 41598_2024_54835_MOESM2_ESM.zip › 10000/train/Negative/01641.jpg]

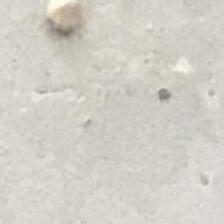

Supplement: Supplementary file 2 — Supplementary Information 2. [file 41598_2024_54835_MOESM2_ESM.zip › 10000/train/Negative/01642.jpg]

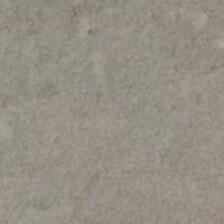

Supplement: Supplementary file 2 — Supplementary Information 2. [file 41598_2024_54835_MOESM2_ESM.zip › 10000/train/Negative/01643.jpg]

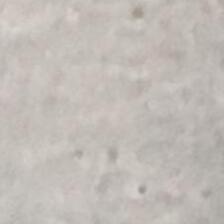

Supplement: Supplementary file 2 — Supplementary Information 2. [file 41598_2024_54835_MOESM2_ESM.zip › 10000/train/Negative/01644.jpg]

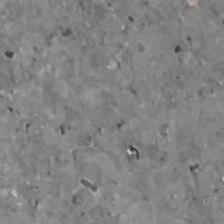

Supplement: Supplementary file 2 — Supplementary Information 2. [file 41598_2024_54835_MOESM2_ESM.zip › 10000/train/Negative/01645.jpg]

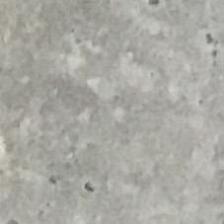

Supplement: Supplementary file 2 — Supplementary Information 2. [file 41598_2024_54835_MOESM2_ESM.zip › 10000/train/Negative/01646.jpg]

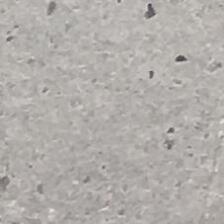

Supplement: Supplementary file 2 — Supplementary Information 2. [file 41598_2024_54835_MOESM2_ESM.zip › 10000/train/Negative/01647.jpg]

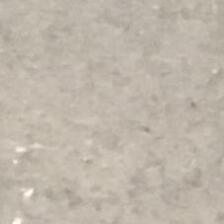

Supplement: Supplementary file 2 — Supplementary Information 2. [file 41598_2024_54835_MOESM2_ESM.zip › 10000/train/Negative/01648.jpg]

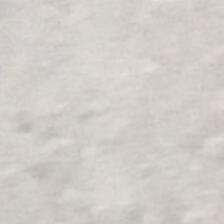

Supplement: Supplementary file 2 — Supplementary Information 2. [file 41598_2024_54835_MOESM2_ESM.zip › 10000/train/Negative/01649.jpg]

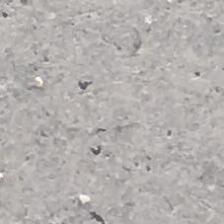

Supplement: Supplementary file 2 — Supplementary Information 2. [file 41598_2024_54835_MOESM2_ESM.zip › 10000/train/Negative/01650.jpg]

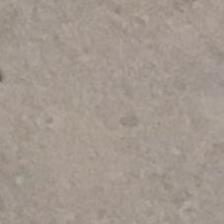

Supplement: Supplementary file 2 — Supplementary Information 2. [file 41598_2024_54835_MOESM2_ESM.zip › 10000/train/Negative/01651.jpg]

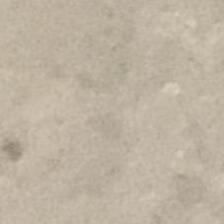

Supplement: Supplementary file 2 — Supplementary Information 2. [file 41598_2024_54835_MOESM2_ESM.zip › 10000/train/Negative/01652.jpg]

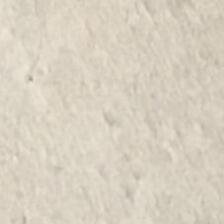

Supplement: Supplementary file 2 — Supplementary Information 2. [file 41598_2024_54835_MOESM2_ESM.zip › 10000/train/Negative/01653.jpg]

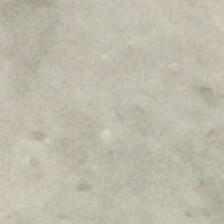

Supplement: Supplementary file 2 — Supplementary Information 2. [file 41598_2024_54835_MOESM2_ESM.zip › 10000/train/Negative/01654.jpg]

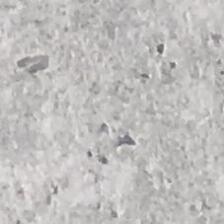

Supplement: Supplementary file 2 — Supplementary Information 2. [file 41598_2024_54835_MOESM2_ESM.zip › 10000/train/Negative/01655.jpg]

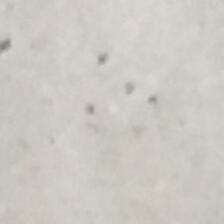

Supplement: Supplementary file 2 — Supplementary Information 2. [file 41598_2024_54835_MOESM2_ESM.zip › 10000/train/Negative/01656.jpg]

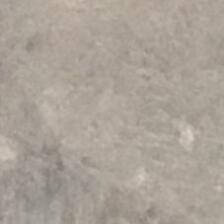

Supplement: Supplementary file 2 — Supplementary Information 2. [file 41598_2024_54835_MOESM2_ESM.zip › 10000/train/Negative/01657.jpg]

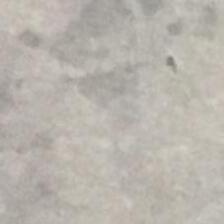

Supplement: Supplementary file 2 — Supplementary Information 2. [file 41598_2024_54835_MOESM2_ESM.zip › 10000/train/Negative/01658.jpg]

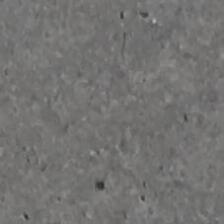

Supplement: Supplementary file 2 — Supplementary Information 2. [file 41598_2024_54835_MOESM2_ESM.zip › 10000/train/Negative/01659.jpg]

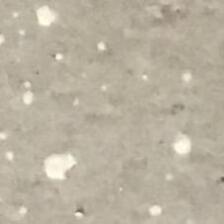

Supplement: Supplementary file 2 — Supplementary Information 2. [file 41598_2024_54835_MOESM2_ESM.zip › 10000/train/Negative/01660.jpg]

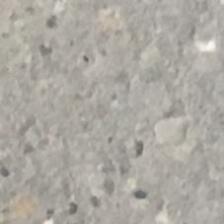

Supplement: Supplementary file 2 — Supplementary Information 2. [file 41598_2024_54835_MOESM2_ESM.zip › 10000/train/Negative/01661.jpg]

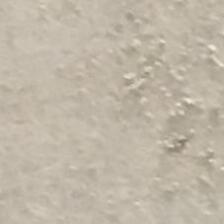

Supplement: Supplementary file 2 — Supplementary Information 2. [file 41598_2024_54835_MOESM2_ESM.zip › 10000/train/Negative/01662.jpg]

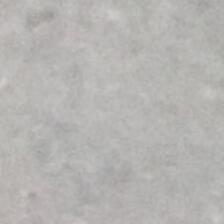

Supplement: Supplementary file 2 — Supplementary Information 2. [file 41598_2024_54835_MOESM2_ESM.zip › 10000/train/Negative/01663.jpg]

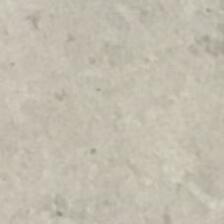

Supplement: Supplementary file 2 — Supplementary Information 2. [file 41598_2024_54835_MOESM2_ESM.zip › 10000/train/Negative/01664.jpg]

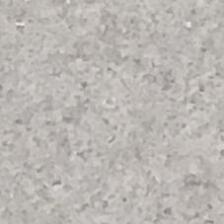

Supplement: Supplementary file 2 — Supplementary Information 2. [file 41598_2024_54835_MOESM2_ESM.zip › 10000/train/Negative/01665.jpg]

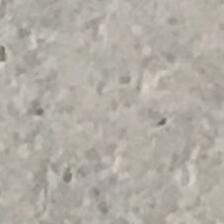

Supplement: Supplementary file 2 — Supplementary Information 2. [file 41598_2024_54835_MOESM2_ESM.zip › 10000/train/Negative/01666.jpg]

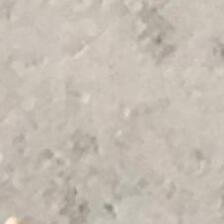

Supplement: Supplementary file 2 — Supplementary Information 2. [file 41598_2024_54835_MOESM2_ESM.zip › 10000/train/Negative/01667.jpg]

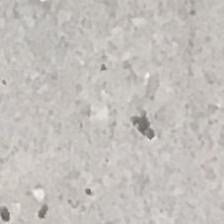

Supplement: Supplementary file 2 — Supplementary Information 2. [file 41598_2024_54835_MOESM2_ESM.zip › 10000/train/Negative/01668.jpg]

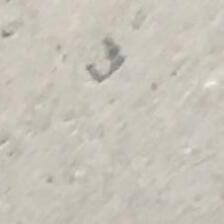

Supplement: Supplementary file 2 — Supplementary Information 2. [file 41598_2024_54835_MOESM2_ESM.zip › 10000/train/Negative/01669.jpg]

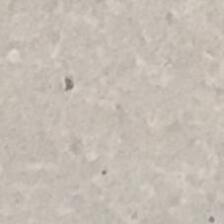

Supplement: Supplementary file 2 — Supplementary Information 2. [file 41598_2024_54835_MOESM2_ESM.zip › 10000/train/Negative/01670.jpg]

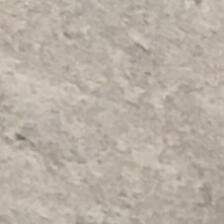

Supplement: Supplementary file 2 — Supplementary Information 2. [file 41598_2024_54835_MOESM2_ESM.zip › 10000/train/Negative/01671.jpg]

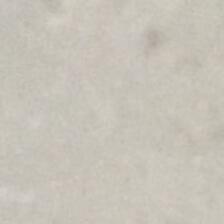

Supplement: Supplementary file 2 — Supplementary Information 2. [file 41598_2024_54835_MOESM2_ESM.zip › 10000/train/Negative/01672.jpg]

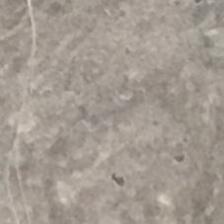

Supplement: Supplementary file 2 — Supplementary Information 2. [file 41598_2024_54835_MOESM2_ESM.zip › 10000/train/Negative/01673.jpg]

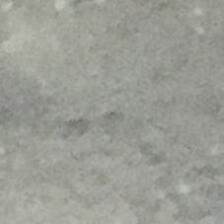

Supplement: Supplementary file 2 — Supplementary Information 2. [file 41598_2024_54835_MOESM2_ESM.zip › 10000/train/Negative/01674.jpg]

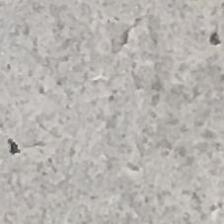

Supplement: Supplementary file 2 — Supplementary Information 2. [file 41598_2024_54835_MOESM2_ESM.zip › 10000/train/Negative/01675.jpg]

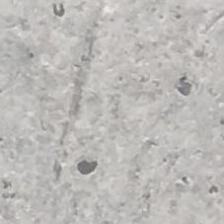

Supplement: Supplementary file 2 — Supplementary Information 2. [file 41598_2024_54835_MOESM2_ESM.zip › 10000/train/Negative/01676.jpg]

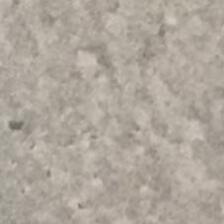

Supplement: Supplementary file 2 — Supplementary Information 2. [file 41598_2024_54835_MOESM2_ESM.zip › 10000/train/Negative/01677.jpg]

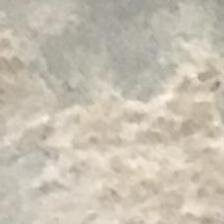

Supplement: Supplementary file 2 — Supplementary Information 2. [file 41598_2024_54835_MOESM2_ESM.zip › 10000/train/Negative/01678.jpg]

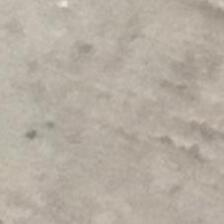

Supplement: Supplementary file 2 — Supplementary Information 2. [file 41598_2024_54835_MOESM2_ESM.zip › 10000/train/Negative/01679.jpg]

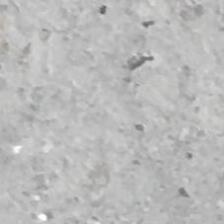

Supplement: Supplementary file 2 — Supplementary Information 2. [file 41598_2024_54835_MOESM2_ESM.zip › 10000/train/Negative/01680.jpg]

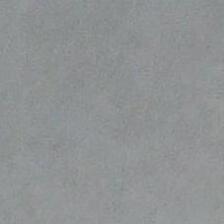

Supplement: Supplementary file 2 — Supplementary Information 2. [file 41598_2024_54835_MOESM2_ESM.zip › 10000/train/Negative/01681.jpg]

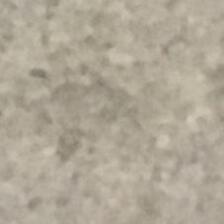

Supplement: Supplementary file 2 — Supplementary Information 2. [file 41598_2024_54835_MOESM2_ESM.zip › 10000/train/Negative/01682.jpg]

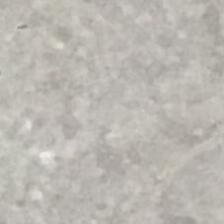

Supplement: Supplementary file 2 — Supplementary Information 2. [file 41598_2024_54835_MOESM2_ESM.zip › 10000/train/Negative/01683.jpg]

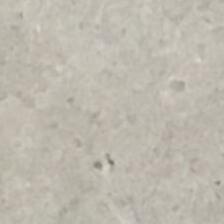

Supplement: Supplementary file 2 — Supplementary Information 2. [file 41598_2024_54835_MOESM2_ESM.zip › 10000/train/Negative/01684.jpg]

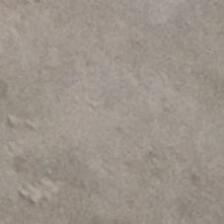

Supplement: Supplementary file 2 — Supplementary Information 2. [file 41598_2024_54835_MOESM2_ESM.zip › 10000/train/Negative/01685.jpg]

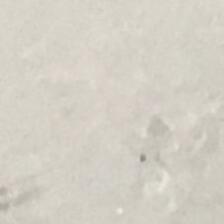

Supplement: Supplementary file 2 — Supplementary Information 2. [file 41598_2024_54835_MOESM2_ESM.zip › 10000/train/Negative/01686.jpg]

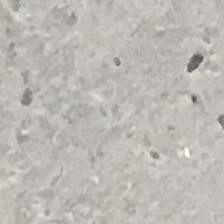

Supplement: Supplementary file 2 — Supplementary Information 2. [file 41598_2024_54835_MOESM2_ESM.zip › 10000/train/Negative/01687.jpg]

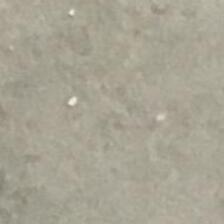

Supplement: Supplementary file 2 — Supplementary Information 2. [file 41598_2024_54835_MOESM2_ESM.zip › 10000/train/Negative/01688.jpg]

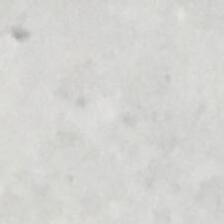

Supplement: Supplementary file 2 — Supplementary Information 2. [file 41598_2024_54835_MOESM2_ESM.zip › 10000/train/Negative/01689.jpg]

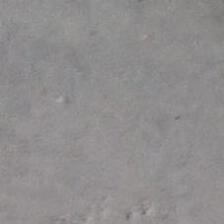

Supplement: Supplementary file 2 — Supplementary Information 2. [file 41598_2024_54835_MOESM2_ESM.zip › 10000/train/Negative/01690.jpg]

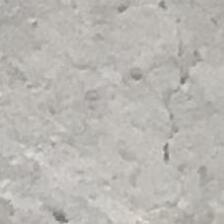

Supplement: Supplementary file 2 — Supplementary Information 2. [file 41598_2024_54835_MOESM2_ESM.zip › 10000/train/Negative/01691.jpg]

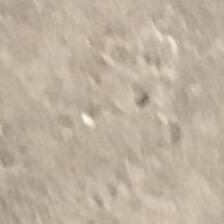

Supplement: Supplementary file 2 — Supplementary Information 2. [file 41598_2024_54835_MOESM2_ESM.zip › 10000/train/Negative/01692.jpg]

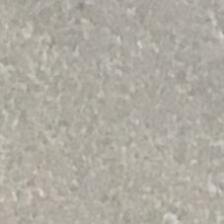

Supplement: Supplementary file 2 — Supplementary Information 2. [file 41598_2024_54835_MOESM2_ESM.zip › 10000/train/Negative/01693.jpg]

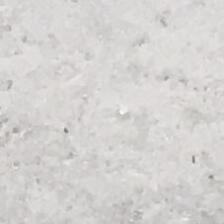

Supplement: Supplementary file 2 — Supplementary Information 2. [file 41598_2024_54835_MOESM2_ESM.zip › 10000/train/Negative/01694.jpg]

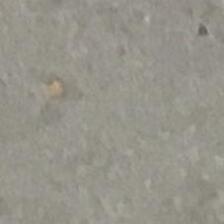

Supplement: Supplementary file 2 — Supplementary Information 2. [file 41598_2024_54835_MOESM2_ESM.zip › 10000/train/Negative/01695.jpg]

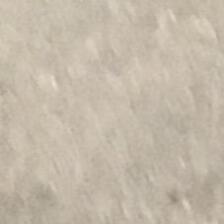

Supplement: Supplementary file 2 — Supplementary Information 2. [file 41598_2024_54835_MOESM2_ESM.zip › 10000/train/Negative/01696.jpg]

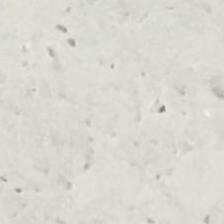

Supplement: Supplementary file 2 — Supplementary Information 2. [file 41598_2024_54835_MOESM2_ESM.zip › 10000/train/Negative/01697.jpg]

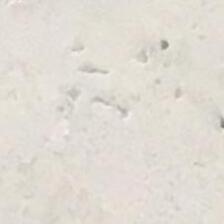

Supplement: Supplementary file 2 — Supplementary Information 2. [file 41598_2024_54835_MOESM2_ESM.zip › 10000/train/Negative/01698.jpg]

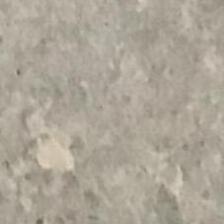

Supplement: Supplementary file 2 — Supplementary Information 2. [file 41598_2024_54835_MOESM2_ESM.zip › 10000/train/Negative/01699.jpg]

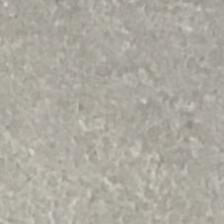

Supplement: Supplementary file 2 — Supplementary Information 2. [file 41598_2024_54835_MOESM2_ESM.zip › 10000/train/Negative/01700.jpg]
