# Supplementary figures and images for: An improved transformer-based concrete crack classification method (part 5 of 7)
Source: Sci Rep. 2024 Mar 14;14:6226. doi: 10.1038/s41598-024-54835-x (PMC10940720; doi:10.1038/s41598-024-54835-x)

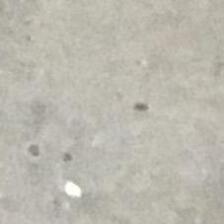

Supplement: Supplementary file 3 — Supplementary Information 3. [file 41598_2024_54835_MOESM3_ESM.zip › 15000/train/Negative/00371.jpg]

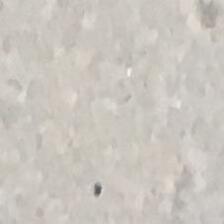

Supplement: Supplementary file 3 — Supplementary Information 3. [file 41598_2024_54835_MOESM3_ESM.zip › 15000/train/Negative/00372.jpg]

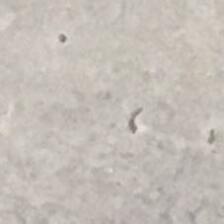

Supplement: Supplementary file 3 — Supplementary Information 3. [file 41598_2024_54835_MOESM3_ESM.zip › 15000/train/Negative/00373.jpg]

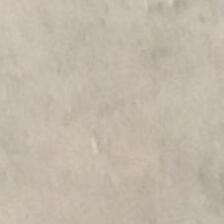

Supplement: Supplementary file 3 — Supplementary Information 3. [file 41598_2024_54835_MOESM3_ESM.zip › 15000/train/Negative/00374.jpg]

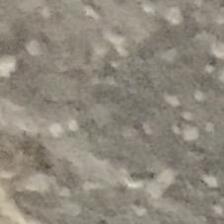

Supplement: Supplementary file 3 — Supplementary Information 3. [file 41598_2024_54835_MOESM3_ESM.zip › 15000/train/Negative/00375.jpg]

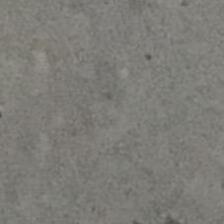

Supplement: Supplementary file 3 — Supplementary Information 3. [file 41598_2024_54835_MOESM3_ESM.zip › 15000/train/Negative/00376.jpg]

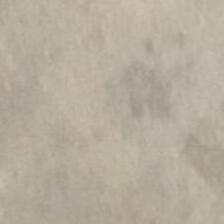

Supplement: Supplementary file 3 — Supplementary Information 3. [file 41598_2024_54835_MOESM3_ESM.zip › 15000/train/Negative/00377.jpg]

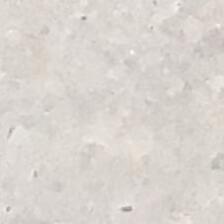

Supplement: Supplementary file 3 — Supplementary Information 3. [file 41598_2024_54835_MOESM3_ESM.zip › 15000/train/Negative/00378.jpg]

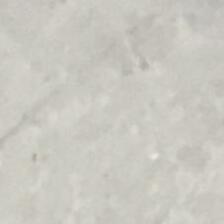

Supplement: Supplementary file 3 — Supplementary Information 3. [file 41598_2024_54835_MOESM3_ESM.zip › 15000/train/Negative/00379.jpg]

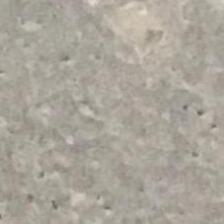

Supplement: Supplementary file 3 — Supplementary Information 3. [file 41598_2024_54835_MOESM3_ESM.zip › 15000/train/Negative/00380.jpg]

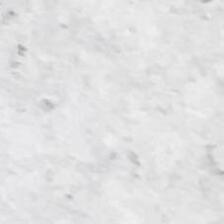

Supplement: Supplementary file 3 — Supplementary Information 3. [file 41598_2024_54835_MOESM3_ESM.zip › 15000/train/Negative/00381.jpg]

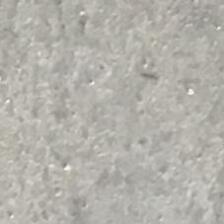

Supplement: Supplementary file 3 — Supplementary Information 3. [file 41598_2024_54835_MOESM3_ESM.zip › 15000/train/Negative/00382.jpg]

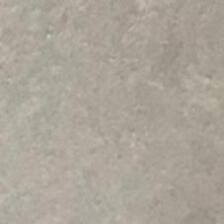

Supplement: Supplementary file 3 — Supplementary Information 3. [file 41598_2024_54835_MOESM3_ESM.zip › 15000/train/Negative/00383.jpg]

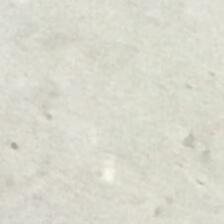

Supplement: Supplementary file 3 — Supplementary Information 3. [file 41598_2024_54835_MOESM3_ESM.zip › 15000/train/Negative/00384.jpg]

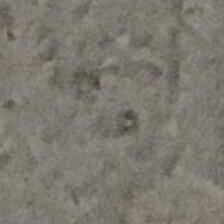

Supplement: Supplementary file 3 — Supplementary Information 3. [file 41598_2024_54835_MOESM3_ESM.zip › 15000/train/Negative/00385.jpg]

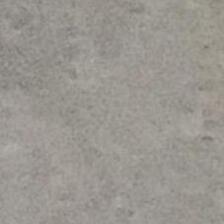

Supplement: Supplementary file 3 — Supplementary Information 3. [file 41598_2024_54835_MOESM3_ESM.zip › 15000/train/Negative/00386.jpg]

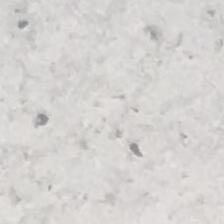

Supplement: Supplementary file 3 — Supplementary Information 3. [file 41598_2024_54835_MOESM3_ESM.zip › 15000/train/Negative/00387.jpg]

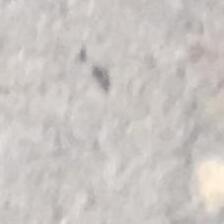

Supplement: Supplementary file 3 — Supplementary Information 3. [file 41598_2024_54835_MOESM3_ESM.zip › 15000/train/Negative/00388.jpg]

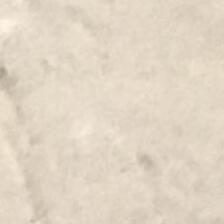

Supplement: Supplementary file 3 — Supplementary Information 3. [file 41598_2024_54835_MOESM3_ESM.zip › 15000/train/Negative/00389.jpg]

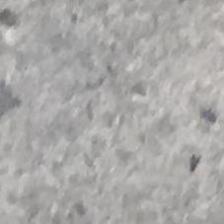

Supplement: Supplementary file 3 — Supplementary Information 3. [file 41598_2024_54835_MOESM3_ESM.zip › 15000/train/Negative/00390.jpg]

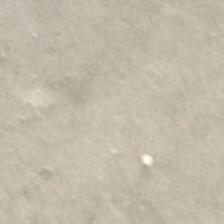

Supplement: Supplementary file 3 — Supplementary Information 3. [file 41598_2024_54835_MOESM3_ESM.zip › 15000/train/Negative/00391.jpg]

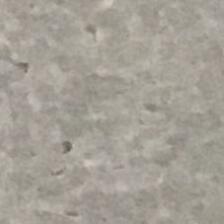

Supplement: Supplementary file 3 — Supplementary Information 3. [file 41598_2024_54835_MOESM3_ESM.zip › 15000/train/Negative/00392.jpg]

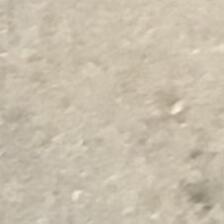

Supplement: Supplementary file 3 — Supplementary Information 3. [file 41598_2024_54835_MOESM3_ESM.zip › 15000/train/Negative/00393.jpg]

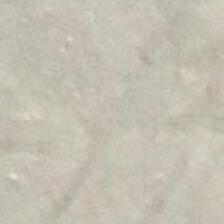

Supplement: Supplementary file 3 — Supplementary Information 3. [file 41598_2024_54835_MOESM3_ESM.zip › 15000/train/Negative/00394.jpg]

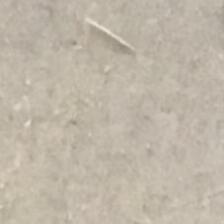

Supplement: Supplementary file 3 — Supplementary Information 3. [file 41598_2024_54835_MOESM3_ESM.zip › 15000/train/Negative/00395.jpg]

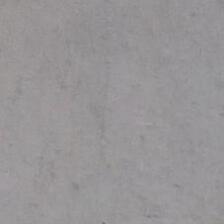

Supplement: Supplementary file 3 — Supplementary Information 3. [file 41598_2024_54835_MOESM3_ESM.zip › 15000/train/Negative/00396.jpg]

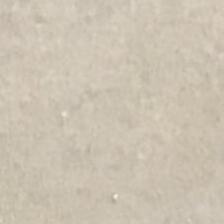

Supplement: Supplementary file 3 — Supplementary Information 3. [file 41598_2024_54835_MOESM3_ESM.zip › 15000/train/Negative/00397.jpg]

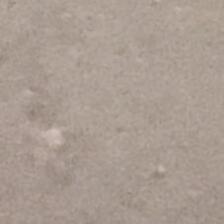

Supplement: Supplementary file 3 — Supplementary Information 3. [file 41598_2024_54835_MOESM3_ESM.zip › 15000/train/Negative/00398.jpg]

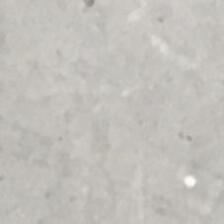

Supplement: Supplementary file 3 — Supplementary Information 3. [file 41598_2024_54835_MOESM3_ESM.zip › 15000/train/Negative/00399.jpg]

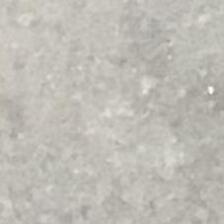

Supplement: Supplementary file 3 — Supplementary Information 3. [file 41598_2024_54835_MOESM3_ESM.zip › 15000/train/Negative/00400.jpg]

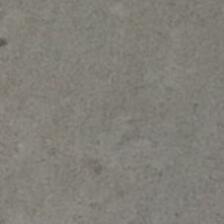

Supplement: Supplementary file 3 — Supplementary Information 3. [file 41598_2024_54835_MOESM3_ESM.zip › 15000/train/Negative/00401.jpg]

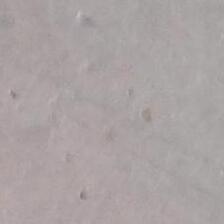

Supplement: Supplementary file 3 — Supplementary Information 3. [file 41598_2024_54835_MOESM3_ESM.zip › 15000/train/Negative/00402.jpg]

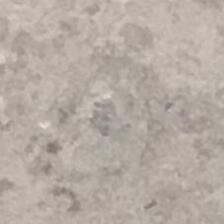

Supplement: Supplementary file 3 — Supplementary Information 3. [file 41598_2024_54835_MOESM3_ESM.zip › 15000/train/Negative/00403.jpg]

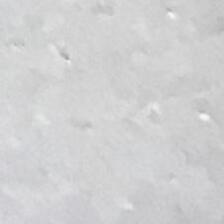

Supplement: Supplementary file 3 — Supplementary Information 3. [file 41598_2024_54835_MOESM3_ESM.zip › 15000/train/Negative/00404.jpg]

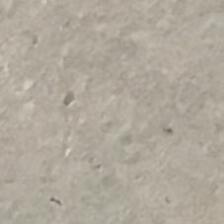

Supplement: Supplementary file 3 — Supplementary Information 3. [file 41598_2024_54835_MOESM3_ESM.zip › 15000/train/Negative/00405.jpg]

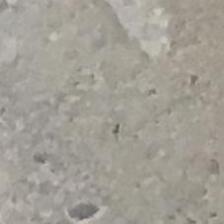

Supplement: Supplementary file 3 — Supplementary Information 3. [file 41598_2024_54835_MOESM3_ESM.zip › 15000/train/Negative/00406.jpg]

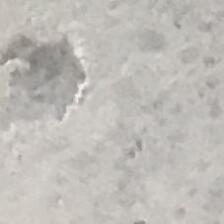

Supplement: Supplementary file 3 — Supplementary Information 3. [file 41598_2024_54835_MOESM3_ESM.zip › 15000/train/Negative/00407.jpg]

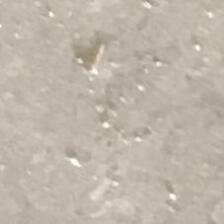

Supplement: Supplementary file 3 — Supplementary Information 3. [file 41598_2024_54835_MOESM3_ESM.zip › 15000/train/Negative/00408.jpg]

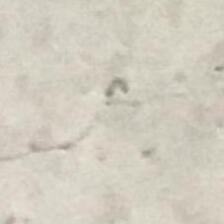

Supplement: Supplementary file 3 — Supplementary Information 3. [file 41598_2024_54835_MOESM3_ESM.zip › 15000/train/Negative/00409.jpg]

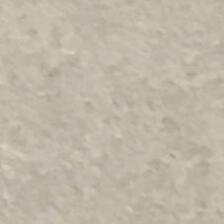

Supplement: Supplementary file 3 — Supplementary Information 3. [file 41598_2024_54835_MOESM3_ESM.zip › 15000/train/Negative/00410.jpg]

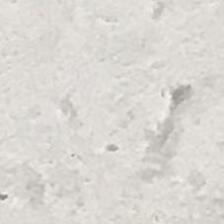

Supplement: Supplementary file 3 — Supplementary Information 3. [file 41598_2024_54835_MOESM3_ESM.zip › 15000/train/Negative/00411.jpg]

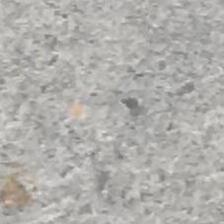

Supplement: Supplementary file 3 — Supplementary Information 3. [file 41598_2024_54835_MOESM3_ESM.zip › 15000/train/Negative/00412.jpg]

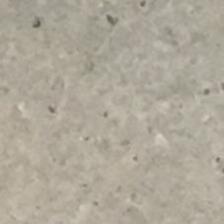

Supplement: Supplementary file 3 — Supplementary Information 3. [file 41598_2024_54835_MOESM3_ESM.zip › 15000/train/Negative/00413.jpg]

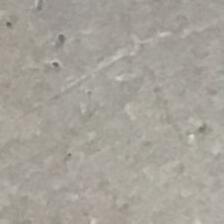

Supplement: Supplementary file 3 — Supplementary Information 3. [file 41598_2024_54835_MOESM3_ESM.zip › 15000/train/Negative/00414.jpg]

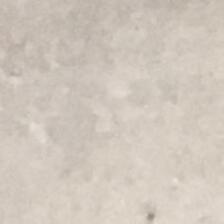

Supplement: Supplementary file 3 — Supplementary Information 3. [file 41598_2024_54835_MOESM3_ESM.zip › 15000/train/Negative/00415.jpg]

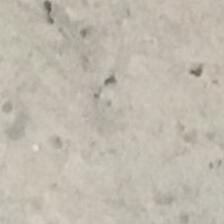

Supplement: Supplementary file 3 — Supplementary Information 3. [file 41598_2024_54835_MOESM3_ESM.zip › 15000/train/Negative/00416.jpg]

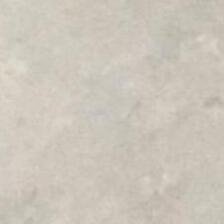

Supplement: Supplementary file 3 — Supplementary Information 3. [file 41598_2024_54835_MOESM3_ESM.zip › 15000/train/Negative/00417.jpg]

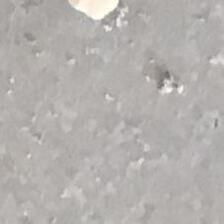

Supplement: Supplementary file 3 — Supplementary Information 3. [file 41598_2024_54835_MOESM3_ESM.zip › 15000/train/Negative/00418.jpg]

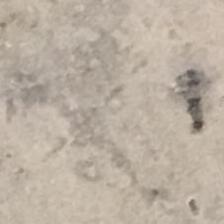

Supplement: Supplementary file 3 — Supplementary Information 3. [file 41598_2024_54835_MOESM3_ESM.zip › 15000/train/Negative/00419.jpg]

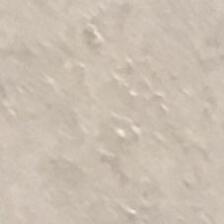

Supplement: Supplementary file 3 — Supplementary Information 3. [file 41598_2024_54835_MOESM3_ESM.zip › 15000/train/Negative/00420.jpg]

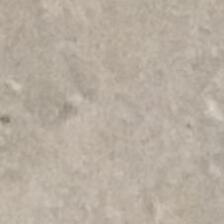

Supplement: Supplementary file 3 — Supplementary Information 3. [file 41598_2024_54835_MOESM3_ESM.zip › 15000/train/Negative/00421.jpg]

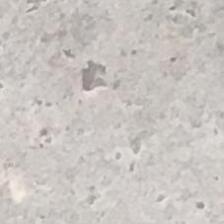

Supplement: Supplementary file 3 — Supplementary Information 3. [file 41598_2024_54835_MOESM3_ESM.zip › 15000/train/Negative/00422.jpg]

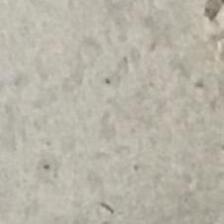

Supplement: Supplementary file 3 — Supplementary Information 3. [file 41598_2024_54835_MOESM3_ESM.zip › 15000/train/Negative/00423.jpg]

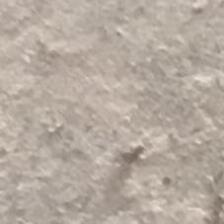

Supplement: Supplementary file 3 — Supplementary Information 3. [file 41598_2024_54835_MOESM3_ESM.zip › 15000/train/Negative/00424.jpg]

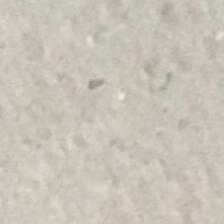

Supplement: Supplementary file 3 — Supplementary Information 3. [file 41598_2024_54835_MOESM3_ESM.zip › 15000/train/Negative/00425.jpg]

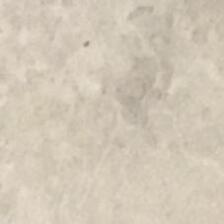

Supplement: Supplementary file 3 — Supplementary Information 3. [file 41598_2024_54835_MOESM3_ESM.zip › 15000/train/Negative/00426.jpg]

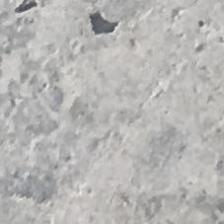

Supplement: Supplementary file 3 — Supplementary Information 3. [file 41598_2024_54835_MOESM3_ESM.zip › 15000/train/Negative/00427.jpg]

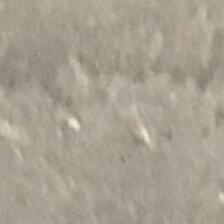

Supplement: Supplementary file 3 — Supplementary Information 3. [file 41598_2024_54835_MOESM3_ESM.zip › 15000/train/Negative/00428.jpg]

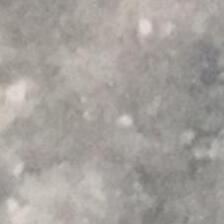

Supplement: Supplementary file 3 — Supplementary Information 3. [file 41598_2024_54835_MOESM3_ESM.zip › 15000/train/Negative/00429.jpg]

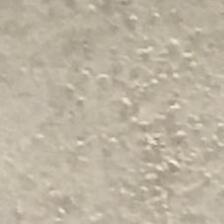

Supplement: Supplementary file 3 — Supplementary Information 3. [file 41598_2024_54835_MOESM3_ESM.zip › 15000/train/Negative/00430.jpg]

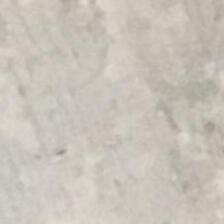

Supplement: Supplementary file 3 — Supplementary Information 3. [file 41598_2024_54835_MOESM3_ESM.zip › 15000/train/Negative/00431.jpg]

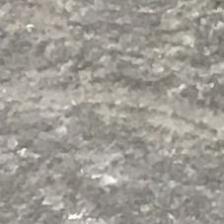

Supplement: Supplementary file 3 — Supplementary Information 3. [file 41598_2024_54835_MOESM3_ESM.zip › 15000/train/Negative/00432.jpg]

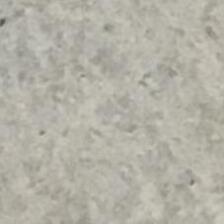

Supplement: Supplementary file 3 — Supplementary Information 3. [file 41598_2024_54835_MOESM3_ESM.zip › 15000/train/Negative/00433.jpg]

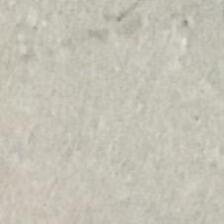

Supplement: Supplementary file 3 — Supplementary Information 3. [file 41598_2024_54835_MOESM3_ESM.zip › 15000/train/Negative/00434.jpg]

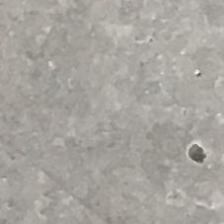

Supplement: Supplementary file 3 — Supplementary Information 3. [file 41598_2024_54835_MOESM3_ESM.zip › 15000/train/Negative/00435.jpg]

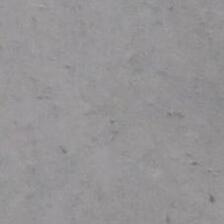

Supplement: Supplementary file 3 — Supplementary Information 3. [file 41598_2024_54835_MOESM3_ESM.zip › 15000/train/Negative/00436.jpg]

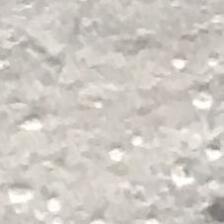

Supplement: Supplementary file 3 — Supplementary Information 3. [file 41598_2024_54835_MOESM3_ESM.zip › 15000/train/Negative/00437.jpg]

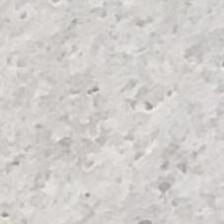

Supplement: Supplementary file 3 — Supplementary Information 3. [file 41598_2024_54835_MOESM3_ESM.zip › 15000/train/Negative/00438.jpg]

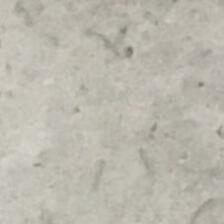

Supplement: Supplementary file 3 — Supplementary Information 3. [file 41598_2024_54835_MOESM3_ESM.zip › 15000/train/Negative/00439.jpg]

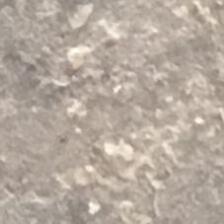

Supplement: Supplementary file 3 — Supplementary Information 3. [file 41598_2024_54835_MOESM3_ESM.zip › 15000/train/Negative/00440.jpg]

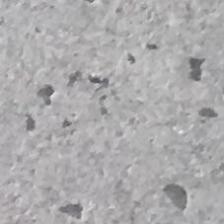

Supplement: Supplementary file 3 — Supplementary Information 3. [file 41598_2024_54835_MOESM3_ESM.zip › 15000/train/Negative/00441.jpg]

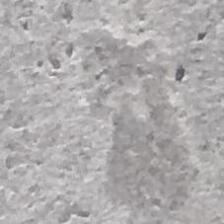

Supplement: Supplementary file 3 — Supplementary Information 3. [file 41598_2024_54835_MOESM3_ESM.zip › 15000/train/Negative/00442.jpg]

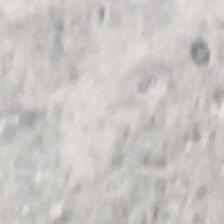

Supplement: Supplementary file 3 — Supplementary Information 3. [file 41598_2024_54835_MOESM3_ESM.zip › 15000/train/Negative/00443.jpg]

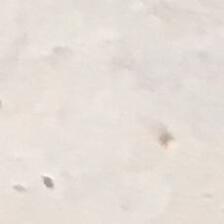

Supplement: Supplementary file 3 — Supplementary Information 3. [file 41598_2024_54835_MOESM3_ESM.zip › 15000/train/Negative/00444.jpg]

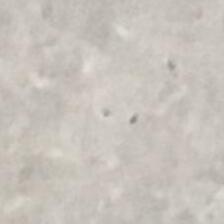

Supplement: Supplementary file 3 — Supplementary Information 3. [file 41598_2024_54835_MOESM3_ESM.zip › 15000/train/Negative/00445.jpg]

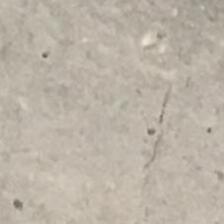

Supplement: Supplementary file 3 — Supplementary Information 3. [file 41598_2024_54835_MOESM3_ESM.zip › 15000/train/Negative/00446.jpg]

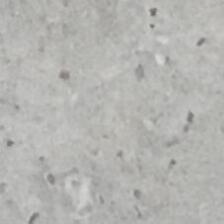

Supplement: Supplementary file 3 — Supplementary Information 3. [file 41598_2024_54835_MOESM3_ESM.zip › 15000/train/Negative/00447.jpg]

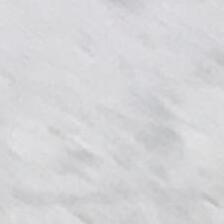

Supplement: Supplementary file 3 — Supplementary Information 3. [file 41598_2024_54835_MOESM3_ESM.zip › 15000/train/Negative/00448.jpg]

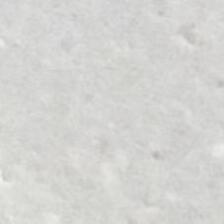

Supplement: Supplementary file 3 — Supplementary Information 3. [file 41598_2024_54835_MOESM3_ESM.zip › 15000/train/Negative/00449.jpg]

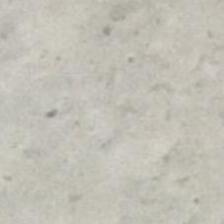

Supplement: Supplementary file 3 — Supplementary Information 3. [file 41598_2024_54835_MOESM3_ESM.zip › 15000/train/Negative/00450.jpg]

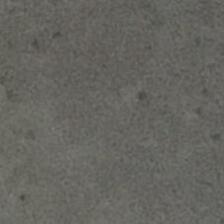

Supplement: Supplementary file 3 — Supplementary Information 3. [file 41598_2024_54835_MOESM3_ESM.zip › 15000/train/Negative/00451.jpg]

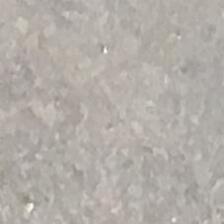

Supplement: Supplementary file 3 — Supplementary Information 3. [file 41598_2024_54835_MOESM3_ESM.zip › 15000/train/Negative/00452.jpg]

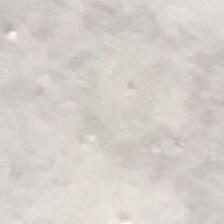

Supplement: Supplementary file 3 — Supplementary Information 3. [file 41598_2024_54835_MOESM3_ESM.zip › 15000/train/Negative/00453.jpg]

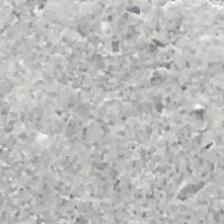

Supplement: Supplementary file 3 — Supplementary Information 3. [file 41598_2024_54835_MOESM3_ESM.zip › 15000/train/Negative/00454.jpg]

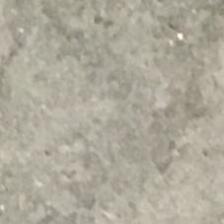

Supplement: Supplementary file 3 — Supplementary Information 3. [file 41598_2024_54835_MOESM3_ESM.zip › 15000/train/Negative/00455.jpg]

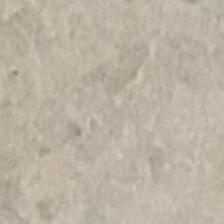

Supplement: Supplementary file 3 — Supplementary Information 3. [file 41598_2024_54835_MOESM3_ESM.zip › 15000/train/Negative/00456.jpg]

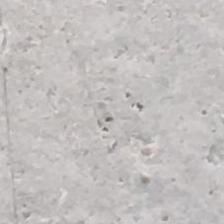

Supplement: Supplementary file 3 — Supplementary Information 3. [file 41598_2024_54835_MOESM3_ESM.zip › 15000/train/Negative/00457.jpg]

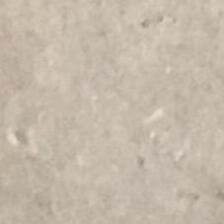

Supplement: Supplementary file 3 — Supplementary Information 3. [file 41598_2024_54835_MOESM3_ESM.zip › 15000/train/Negative/00458.jpg]

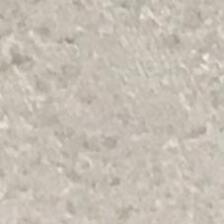

Supplement: Supplementary file 3 — Supplementary Information 3. [file 41598_2024_54835_MOESM3_ESM.zip › 15000/train/Negative/00459.jpg]

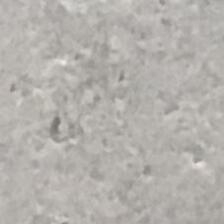

Supplement: Supplementary file 3 — Supplementary Information 3. [file 41598_2024_54835_MOESM3_ESM.zip › 15000/train/Negative/00460.jpg]

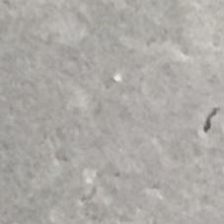

Supplement: Supplementary file 4 — Supplementary Information 4. [file 41598_2024_54835_MOESM4_ESM.zip › ╩2╛▌╝» - ╕▒▒╛/train/Negative/00001.jpg]

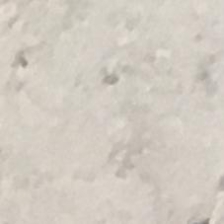

Supplement: Supplementary file 4 — Supplementary Information 4. [file 41598_2024_54835_MOESM4_ESM.zip › ╩2╛▌╝» - ╕▒▒╛/train/Negative/00002.jpg]

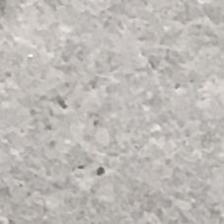

Supplement: Supplementary file 4 — Supplementary Information 4. [file 41598_2024_54835_MOESM4_ESM.zip › ╩2╛▌╝» - ╕▒▒╛/train/Negative/00003.jpg]

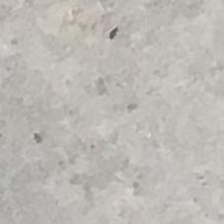

Supplement: Supplementary file 4 — Supplementary Information 4. [file 41598_2024_54835_MOESM4_ESM.zip › ╩2╛▌╝» - ╕▒▒╛/train/Negative/00004.jpg]

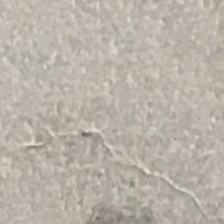

Supplement: Supplementary file 4 — Supplementary Information 4. [file 41598_2024_54835_MOESM4_ESM.zip › ╩2╛▌╝» - ╕▒▒╛/train/Negative/00005.jpg]

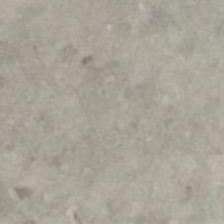

Supplement: Supplementary file 4 — Supplementary Information 4. [file 41598_2024_54835_MOESM4_ESM.zip › ╩2╛▌╝» - ╕▒▒╛/train/Negative/00006.jpg]

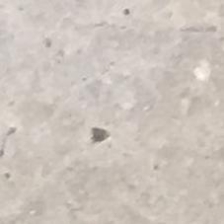

Supplement: Supplementary file 4 — Supplementary Information 4. [file 41598_2024_54835_MOESM4_ESM.zip › ╩2╛▌╝» - ╕▒▒╛/train/Negative/00007.jpg]

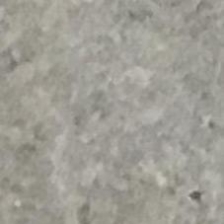

Supplement: Supplementary file 4 — Supplementary Information 4. [file 41598_2024_54835_MOESM4_ESM.zip › ╩2╛▌╝» - ╕▒▒╛/train/Negative/00008.jpg]

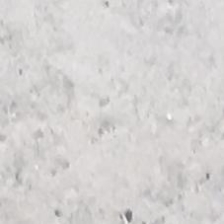

Supplement: Supplementary file 4 — Supplementary Information 4. [file 41598_2024_54835_MOESM4_ESM.zip › ╩2╛▌╝» - ╕▒▒╛/train/Negative/00009.jpg]

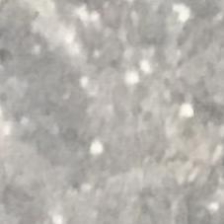

Supplement: Supplementary file 4 — Supplementary Information 4. [file 41598_2024_54835_MOESM4_ESM.zip › ╩2╛▌╝» - ╕▒▒╛/train/Negative/00010.jpg]
