# Supplementary figures and images for: An improved transformer-based concrete crack classification method (part 4 of 7)
Source: Sci Rep. 2024 Mar 14;14:6226. doi: 10.1038/s41598-024-54835-x (PMC10940720; doi:10.1038/s41598-024-54835-x)

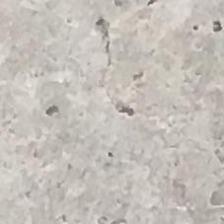

Supplement: Supplementary file 3 — Supplementary Information 3. [file 41598_2024_54835_MOESM3_ESM.zip › 15000/train/Negative/00271.jpg]

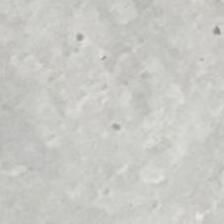

Supplement: Supplementary file 3 — Supplementary Information 3. [file 41598_2024_54835_MOESM3_ESM.zip › 15000/train/Negative/00272.jpg]

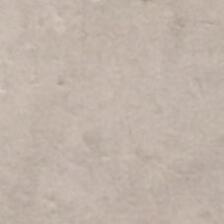

Supplement: Supplementary file 3 — Supplementary Information 3. [file 41598_2024_54835_MOESM3_ESM.zip › 15000/train/Negative/00273.jpg]

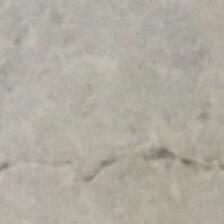

Supplement: Supplementary file 3 — Supplementary Information 3. [file 41598_2024_54835_MOESM3_ESM.zip › 15000/train/Negative/00274.jpg]

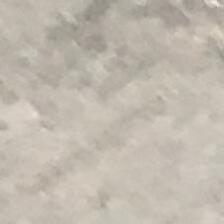

Supplement: Supplementary file 3 — Supplementary Information 3. [file 41598_2024_54835_MOESM3_ESM.zip › 15000/train/Negative/00275.jpg]

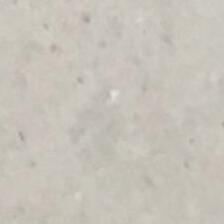

Supplement: Supplementary file 3 — Supplementary Information 3. [file 41598_2024_54835_MOESM3_ESM.zip › 15000/train/Negative/00276.jpg]

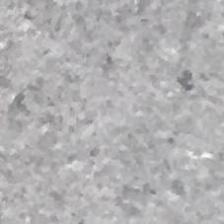

Supplement: Supplementary file 3 — Supplementary Information 3. [file 41598_2024_54835_MOESM3_ESM.zip › 15000/train/Negative/00277.jpg]

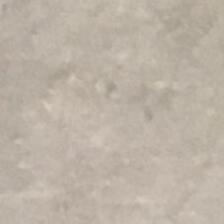

Supplement: Supplementary file 3 — Supplementary Information 3. [file 41598_2024_54835_MOESM3_ESM.zip › 15000/train/Negative/00278.jpg]

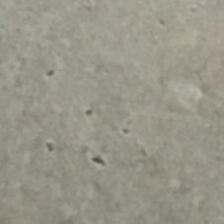

Supplement: Supplementary file 3 — Supplementary Information 3. [file 41598_2024_54835_MOESM3_ESM.zip › 15000/train/Negative/00279.jpg]

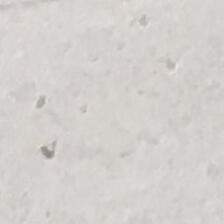

Supplement: Supplementary file 3 — Supplementary Information 3. [file 41598_2024_54835_MOESM3_ESM.zip › 15000/train/Negative/00280.jpg]

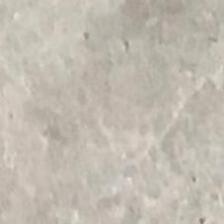

Supplement: Supplementary file 3 — Supplementary Information 3. [file 41598_2024_54835_MOESM3_ESM.zip › 15000/train/Negative/00281.jpg]

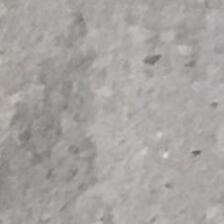

Supplement: Supplementary file 3 — Supplementary Information 3. [file 41598_2024_54835_MOESM3_ESM.zip › 15000/train/Negative/00282.jpg]

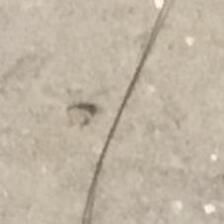

Supplement: Supplementary file 3 — Supplementary Information 3. [file 41598_2024_54835_MOESM3_ESM.zip › 15000/train/Negative/00283.jpg]

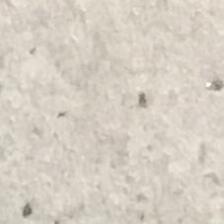

Supplement: Supplementary file 3 — Supplementary Information 3. [file 41598_2024_54835_MOESM3_ESM.zip › 15000/train/Negative/00284.jpg]

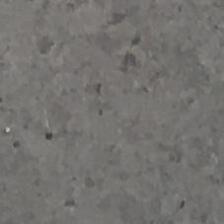

Supplement: Supplementary file 3 — Supplementary Information 3. [file 41598_2024_54835_MOESM3_ESM.zip › 15000/train/Negative/00285.jpg]

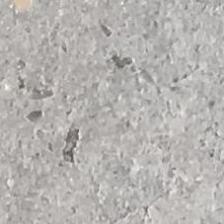

Supplement: Supplementary file 3 — Supplementary Information 3. [file 41598_2024_54835_MOESM3_ESM.zip › 15000/train/Negative/00286.jpg]

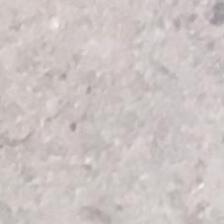

Supplement: Supplementary file 3 — Supplementary Information 3. [file 41598_2024_54835_MOESM3_ESM.zip › 15000/train/Negative/00287.jpg]

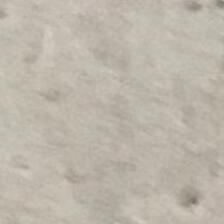

Supplement: Supplementary file 3 — Supplementary Information 3. [file 41598_2024_54835_MOESM3_ESM.zip › 15000/train/Negative/00288.jpg]

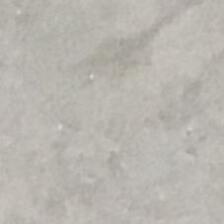

Supplement: Supplementary file 3 — Supplementary Information 3. [file 41598_2024_54835_MOESM3_ESM.zip › 15000/train/Negative/00289.jpg]

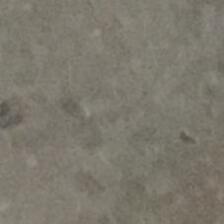

Supplement: Supplementary file 3 — Supplementary Information 3. [file 41598_2024_54835_MOESM3_ESM.zip › 15000/train/Negative/00290.jpg]

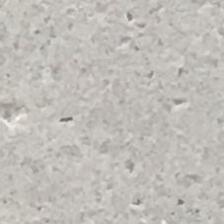

Supplement: Supplementary file 3 — Supplementary Information 3. [file 41598_2024_54835_MOESM3_ESM.zip › 15000/train/Negative/00291.jpg]

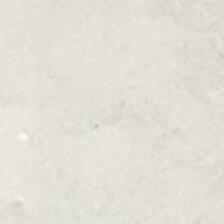

Supplement: Supplementary file 3 — Supplementary Information 3. [file 41598_2024_54835_MOESM3_ESM.zip › 15000/train/Negative/00292.jpg]

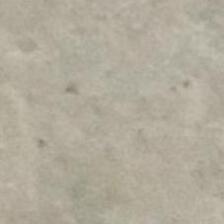

Supplement: Supplementary file 3 — Supplementary Information 3. [file 41598_2024_54835_MOESM3_ESM.zip › 15000/train/Negative/00293.jpg]

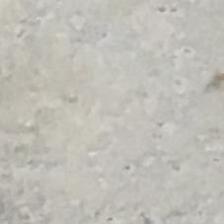

Supplement: Supplementary file 3 — Supplementary Information 3. [file 41598_2024_54835_MOESM3_ESM.zip › 15000/train/Negative/00294.jpg]

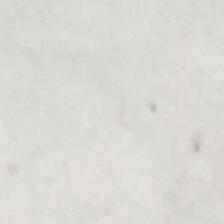

Supplement: Supplementary file 3 — Supplementary Information 3. [file 41598_2024_54835_MOESM3_ESM.zip › 15000/train/Negative/00295.jpg]

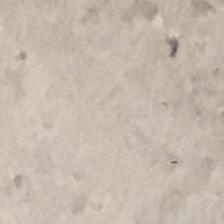

Supplement: Supplementary file 3 — Supplementary Information 3. [file 41598_2024_54835_MOESM3_ESM.zip › 15000/train/Negative/00296.jpg]

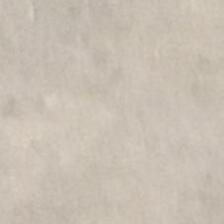

Supplement: Supplementary file 3 — Supplementary Information 3. [file 41598_2024_54835_MOESM3_ESM.zip › 15000/train/Negative/00297.jpg]

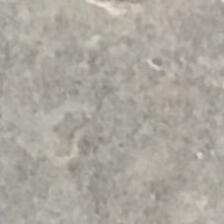

Supplement: Supplementary file 3 — Supplementary Information 3. [file 41598_2024_54835_MOESM3_ESM.zip › 15000/train/Negative/00298.jpg]

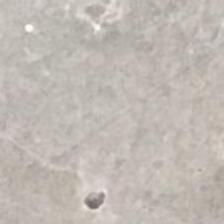

Supplement: Supplementary file 3 — Supplementary Information 3. [file 41598_2024_54835_MOESM3_ESM.zip › 15000/train/Negative/00299.jpg]

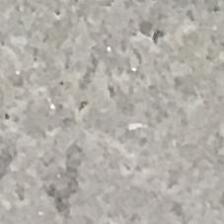

Supplement: Supplementary file 3 — Supplementary Information 3. [file 41598_2024_54835_MOESM3_ESM.zip › 15000/train/Negative/00300.jpg]

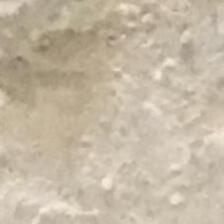

Supplement: Supplementary file 3 — Supplementary Information 3. [file 41598_2024_54835_MOESM3_ESM.zip › 15000/train/Negative/00301.jpg]

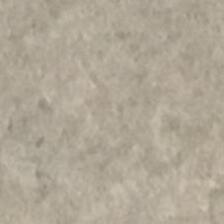

Supplement: Supplementary file 3 — Supplementary Information 3. [file 41598_2024_54835_MOESM3_ESM.zip › 15000/train/Negative/00302.jpg]

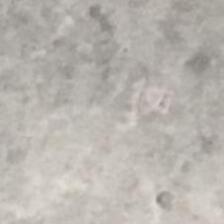

Supplement: Supplementary file 3 — Supplementary Information 3. [file 41598_2024_54835_MOESM3_ESM.zip › 15000/train/Negative/00303.jpg]

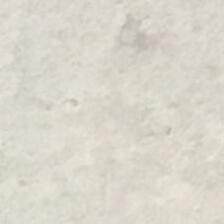

Supplement: Supplementary file 3 — Supplementary Information 3. [file 41598_2024_54835_MOESM3_ESM.zip › 15000/train/Negative/00304.jpg]

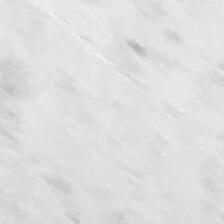

Supplement: Supplementary file 3 — Supplementary Information 3. [file 41598_2024_54835_MOESM3_ESM.zip › 15000/train/Negative/00305.jpg]

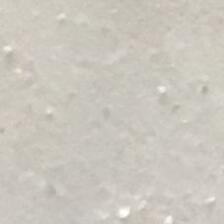

Supplement: Supplementary file 3 — Supplementary Information 3. [file 41598_2024_54835_MOESM3_ESM.zip › 15000/train/Negative/00306.jpg]

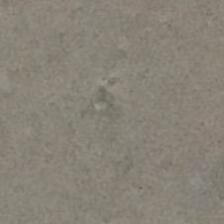

Supplement: Supplementary file 3 — Supplementary Information 3. [file 41598_2024_54835_MOESM3_ESM.zip › 15000/train/Negative/00307.jpg]

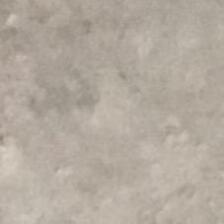

Supplement: Supplementary file 3 — Supplementary Information 3. [file 41598_2024_54835_MOESM3_ESM.zip › 15000/train/Negative/00308.jpg]

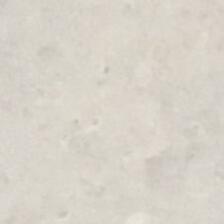

Supplement: Supplementary file 3 — Supplementary Information 3. [file 41598_2024_54835_MOESM3_ESM.zip › 15000/train/Negative/00309.jpg]

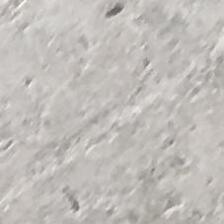

Supplement: Supplementary file 3 — Supplementary Information 3. [file 41598_2024_54835_MOESM3_ESM.zip › 15000/train/Negative/00310.jpg]

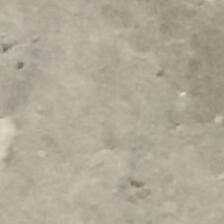

Supplement: Supplementary file 3 — Supplementary Information 3. [file 41598_2024_54835_MOESM3_ESM.zip › 15000/train/Negative/00311.jpg]

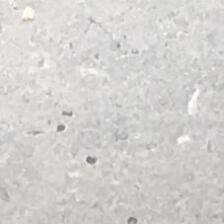

Supplement: Supplementary file 3 — Supplementary Information 3. [file 41598_2024_54835_MOESM3_ESM.zip › 15000/train/Negative/00312.jpg]

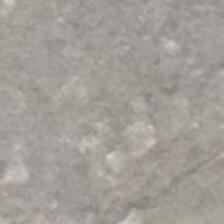

Supplement: Supplementary file 3 — Supplementary Information 3. [file 41598_2024_54835_MOESM3_ESM.zip › 15000/train/Negative/00313.jpg]

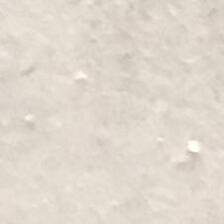

Supplement: Supplementary file 3 — Supplementary Information 3. [file 41598_2024_54835_MOESM3_ESM.zip › 15000/train/Negative/00314.jpg]

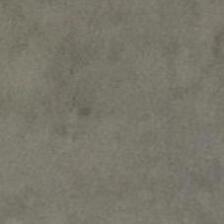

Supplement: Supplementary file 3 — Supplementary Information 3. [file 41598_2024_54835_MOESM3_ESM.zip › 15000/train/Negative/00315.jpg]

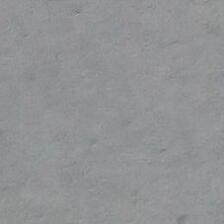

Supplement: Supplementary file 3 — Supplementary Information 3. [file 41598_2024_54835_MOESM3_ESM.zip › 15000/train/Negative/00316.jpg]

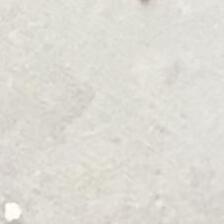

Supplement: Supplementary file 3 — Supplementary Information 3. [file 41598_2024_54835_MOESM3_ESM.zip › 15000/train/Negative/00317.jpg]

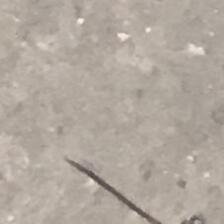

Supplement: Supplementary file 3 — Supplementary Information 3. [file 41598_2024_54835_MOESM3_ESM.zip › 15000/train/Negative/00318.jpg]

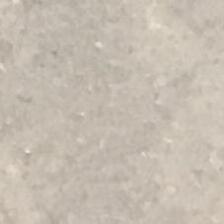

Supplement: Supplementary file 3 — Supplementary Information 3. [file 41598_2024_54835_MOESM3_ESM.zip › 15000/train/Negative/00319.jpg]

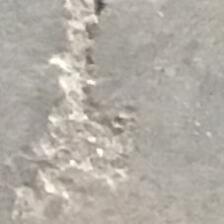

Supplement: Supplementary file 3 — Supplementary Information 3. [file 41598_2024_54835_MOESM3_ESM.zip › 15000/train/Negative/00320.jpg]

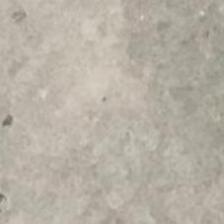

Supplement: Supplementary file 3 — Supplementary Information 3. [file 41598_2024_54835_MOESM3_ESM.zip › 15000/train/Negative/00321.jpg]

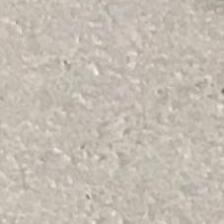

Supplement: Supplementary file 3 — Supplementary Information 3. [file 41598_2024_54835_MOESM3_ESM.zip › 15000/train/Negative/00322.jpg]

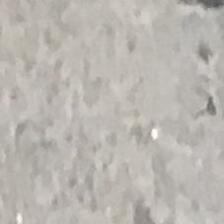

Supplement: Supplementary file 3 — Supplementary Information 3. [file 41598_2024_54835_MOESM3_ESM.zip › 15000/train/Negative/00323.jpg]

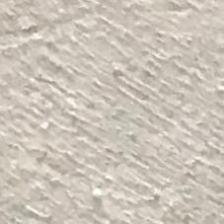

Supplement: Supplementary file 3 — Supplementary Information 3. [file 41598_2024_54835_MOESM3_ESM.zip › 15000/train/Negative/00324.jpg]

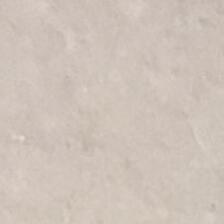

Supplement: Supplementary file 3 — Supplementary Information 3. [file 41598_2024_54835_MOESM3_ESM.zip › 15000/train/Negative/00325.jpg]

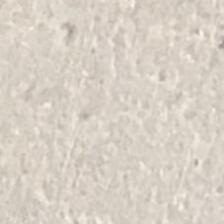

Supplement: Supplementary file 3 — Supplementary Information 3. [file 41598_2024_54835_MOESM3_ESM.zip › 15000/train/Negative/00326.jpg]

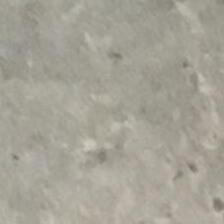

Supplement: Supplementary file 3 — Supplementary Information 3. [file 41598_2024_54835_MOESM3_ESM.zip › 15000/train/Negative/00327.jpg]

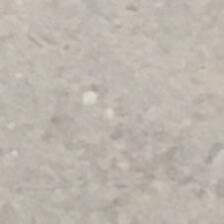

Supplement: Supplementary file 3 — Supplementary Information 3. [file 41598_2024_54835_MOESM3_ESM.zip › 15000/train/Negative/00328.jpg]

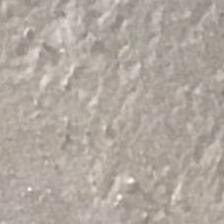

Supplement: Supplementary file 3 — Supplementary Information 3. [file 41598_2024_54835_MOESM3_ESM.zip › 15000/train/Negative/00329.jpg]

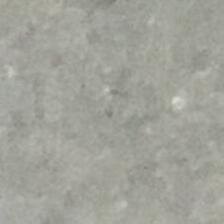

Supplement: Supplementary file 3 — Supplementary Information 3. [file 41598_2024_54835_MOESM3_ESM.zip › 15000/train/Negative/00330.jpg]

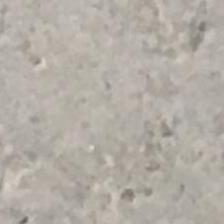

Supplement: Supplementary file 3 — Supplementary Information 3. [file 41598_2024_54835_MOESM3_ESM.zip › 15000/train/Negative/00331.jpg]

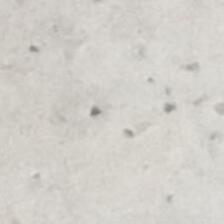

Supplement: Supplementary file 3 — Supplementary Information 3. [file 41598_2024_54835_MOESM3_ESM.zip › 15000/train/Negative/00332.jpg]

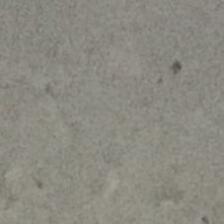

Supplement: Supplementary file 3 — Supplementary Information 3. [file 41598_2024_54835_MOESM3_ESM.zip › 15000/train/Negative/00333.jpg]

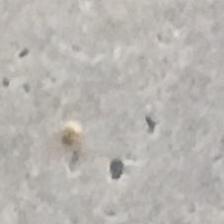

Supplement: Supplementary file 3 — Supplementary Information 3. [file 41598_2024_54835_MOESM3_ESM.zip › 15000/train/Negative/00334.jpg]

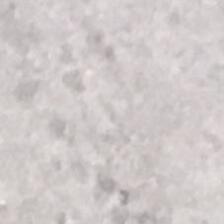

Supplement: Supplementary file 3 — Supplementary Information 3. [file 41598_2024_54835_MOESM3_ESM.zip › 15000/train/Negative/00335.jpg]

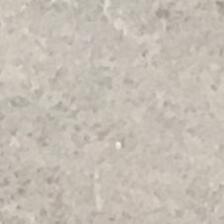

Supplement: Supplementary file 3 — Supplementary Information 3. [file 41598_2024_54835_MOESM3_ESM.zip › 15000/train/Negative/00336.jpg]

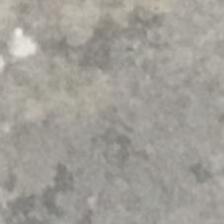

Supplement: Supplementary file 3 — Supplementary Information 3. [file 41598_2024_54835_MOESM3_ESM.zip › 15000/train/Negative/00337.jpg]

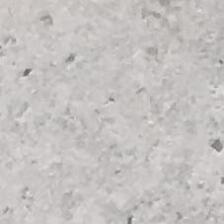

Supplement: Supplementary file 3 — Supplementary Information 3. [file 41598_2024_54835_MOESM3_ESM.zip › 15000/train/Negative/00338.jpg]

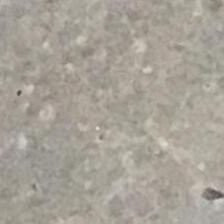

Supplement: Supplementary file 3 — Supplementary Information 3. [file 41598_2024_54835_MOESM3_ESM.zip › 15000/train/Negative/00339.jpg]

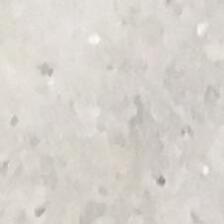

Supplement: Supplementary file 3 — Supplementary Information 3. [file 41598_2024_54835_MOESM3_ESM.zip › 15000/train/Negative/00340.jpg]

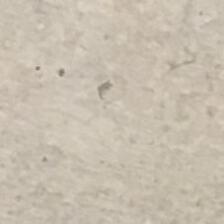

Supplement: Supplementary file 3 — Supplementary Information 3. [file 41598_2024_54835_MOESM3_ESM.zip › 15000/train/Negative/00341.jpg]

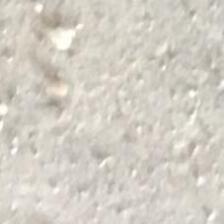

Supplement: Supplementary file 3 — Supplementary Information 3. [file 41598_2024_54835_MOESM3_ESM.zip › 15000/train/Negative/00342.jpg]

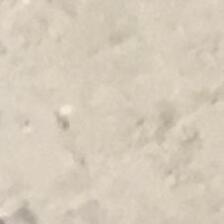

Supplement: Supplementary file 3 — Supplementary Information 3. [file 41598_2024_54835_MOESM3_ESM.zip › 15000/train/Negative/00343.jpg]

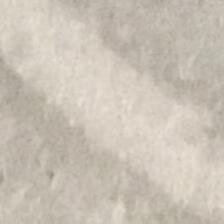

Supplement: Supplementary file 3 — Supplementary Information 3. [file 41598_2024_54835_MOESM3_ESM.zip › 15000/train/Negative/00344.jpg]

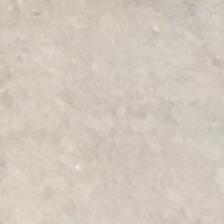

Supplement: Supplementary file 3 — Supplementary Information 3. [file 41598_2024_54835_MOESM3_ESM.zip › 15000/train/Negative/00345.jpg]

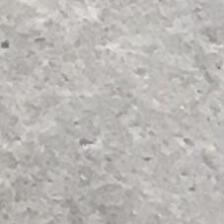

Supplement: Supplementary file 3 — Supplementary Information 3. [file 41598_2024_54835_MOESM3_ESM.zip › 15000/train/Negative/00346.jpg]

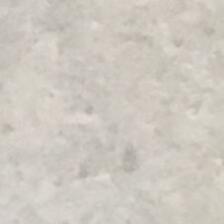

Supplement: Supplementary file 3 — Supplementary Information 3. [file 41598_2024_54835_MOESM3_ESM.zip › 15000/train/Negative/00347.jpg]

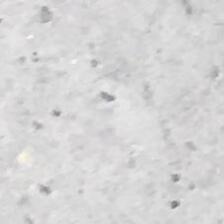

Supplement: Supplementary file 3 — Supplementary Information 3. [file 41598_2024_54835_MOESM3_ESM.zip › 15000/train/Negative/00348.jpg]

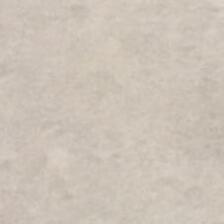

Supplement: Supplementary file 3 — Supplementary Information 3. [file 41598_2024_54835_MOESM3_ESM.zip › 15000/train/Negative/00349.jpg]

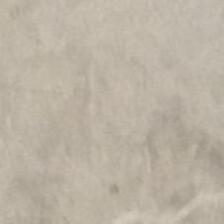

Supplement: Supplementary file 3 — Supplementary Information 3. [file 41598_2024_54835_MOESM3_ESM.zip › 15000/train/Negative/00350.jpg]

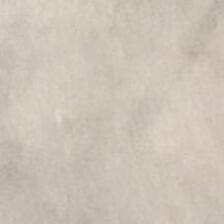

Supplement: Supplementary file 3 — Supplementary Information 3. [file 41598_2024_54835_MOESM3_ESM.zip › 15000/train/Negative/00351.jpg]

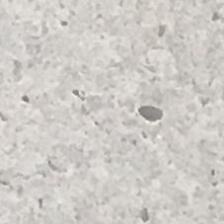

Supplement: Supplementary file 3 — Supplementary Information 3. [file 41598_2024_54835_MOESM3_ESM.zip › 15000/train/Negative/00352.jpg]

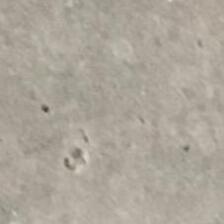

Supplement: Supplementary file 3 — Supplementary Information 3. [file 41598_2024_54835_MOESM3_ESM.zip › 15000/train/Negative/00353.jpg]

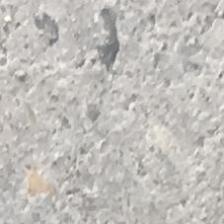

Supplement: Supplementary file 3 — Supplementary Information 3. [file 41598_2024_54835_MOESM3_ESM.zip › 15000/train/Negative/00354.jpg]

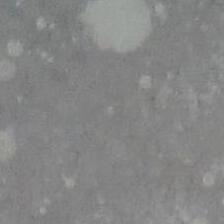

Supplement: Supplementary file 3 — Supplementary Information 3. [file 41598_2024_54835_MOESM3_ESM.zip › 15000/train/Negative/00355.jpg]

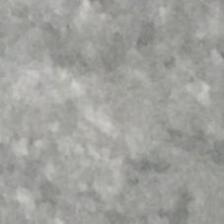

Supplement: Supplementary file 3 — Supplementary Information 3. [file 41598_2024_54835_MOESM3_ESM.zip › 15000/train/Negative/00356.jpg]

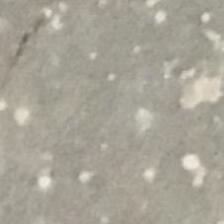

Supplement: Supplementary file 3 — Supplementary Information 3. [file 41598_2024_54835_MOESM3_ESM.zip › 15000/train/Negative/00357.jpg]

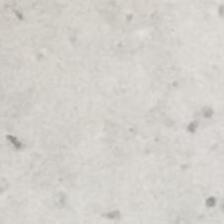

Supplement: Supplementary file 3 — Supplementary Information 3. [file 41598_2024_54835_MOESM3_ESM.zip › 15000/train/Negative/00358.jpg]

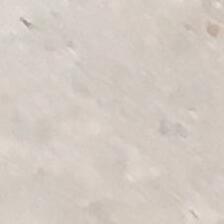

Supplement: Supplementary file 3 — Supplementary Information 3. [file 41598_2024_54835_MOESM3_ESM.zip › 15000/train/Negative/00359.jpg]

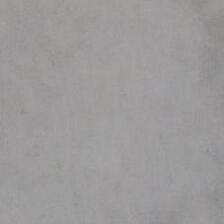

Supplement: Supplementary file 3 — Supplementary Information 3. [file 41598_2024_54835_MOESM3_ESM.zip › 15000/train/Negative/00360.jpg]

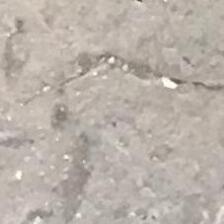

Supplement: Supplementary file 3 — Supplementary Information 3. [file 41598_2024_54835_MOESM3_ESM.zip › 15000/train/Negative/00361.jpg]

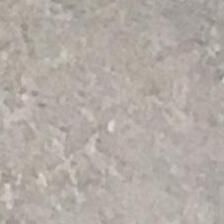

Supplement: Supplementary file 3 — Supplementary Information 3. [file 41598_2024_54835_MOESM3_ESM.zip › 15000/train/Negative/00362.jpg]

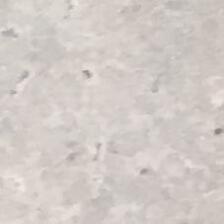

Supplement: Supplementary file 3 — Supplementary Information 3. [file 41598_2024_54835_MOESM3_ESM.zip › 15000/train/Negative/00363.jpg]

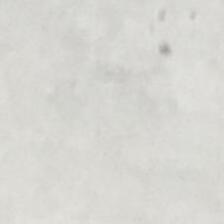

Supplement: Supplementary file 3 — Supplementary Information 3. [file 41598_2024_54835_MOESM3_ESM.zip › 15000/train/Negative/00364.jpg]

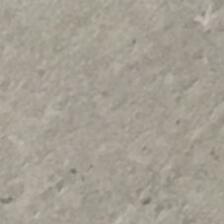

Supplement: Supplementary file 3 — Supplementary Information 3. [file 41598_2024_54835_MOESM3_ESM.zip › 15000/train/Negative/00365.jpg]

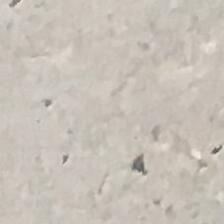

Supplement: Supplementary file 3 — Supplementary Information 3. [file 41598_2024_54835_MOESM3_ESM.zip › 15000/train/Negative/00366.jpg]

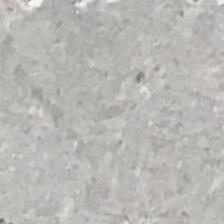

Supplement: Supplementary file 3 — Supplementary Information 3. [file 41598_2024_54835_MOESM3_ESM.zip › 15000/train/Negative/00367.jpg]

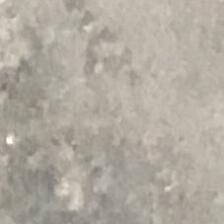

Supplement: Supplementary file 3 — Supplementary Information 3. [file 41598_2024_54835_MOESM3_ESM.zip › 15000/train/Negative/00368.jpg]

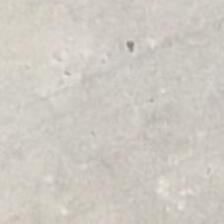

Supplement: Supplementary file 3 — Supplementary Information 3. [file 41598_2024_54835_MOESM3_ESM.zip › 15000/train/Negative/00369.jpg]

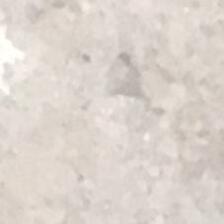

Supplement: Supplementary file 3 — Supplementary Information 3. [file 41598_2024_54835_MOESM3_ESM.zip › 15000/train/Negative/00370.jpg]
