# Supplementary figures and images for: An improved transformer-based concrete crack classification method (part 7 of 7)
Source: Sci Rep. 2024 Mar 14;14:6226. doi: 10.1038/s41598-024-54835-x (PMC10940720; doi:10.1038/s41598-024-54835-x)

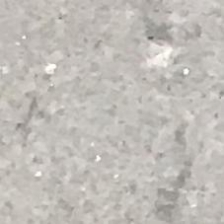

Supplement: Supplementary file 4 — Supplementary Information 4. [file 41598_2024_54835_MOESM4_ESM.zip › ╩2╛▌╝» - ╕▒▒╛/train/Negative/00111.jpg]

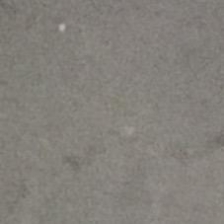

Supplement: Supplementary file 4 — Supplementary Information 4. [file 41598_2024_54835_MOESM4_ESM.zip › ╩2╛▌╝» - ╕▒▒╛/train/Negative/00112.jpg]

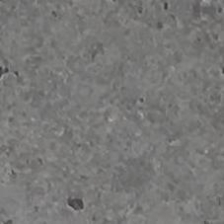

Supplement: Supplementary file 4 — Supplementary Information 4. [file 41598_2024_54835_MOESM4_ESM.zip › ╩2╛▌╝» - ╕▒▒╛/train/Negative/00113.jpg]

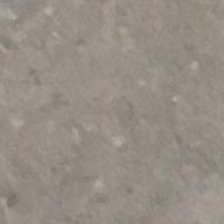

Supplement: Supplementary file 4 — Supplementary Information 4. [file 41598_2024_54835_MOESM4_ESM.zip › ╩2╛▌╝» - ╕▒▒╛/train/Negative/00114.jpg]

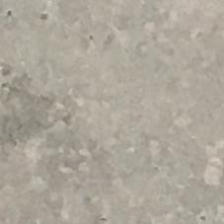

Supplement: Supplementary file 4 — Supplementary Information 4. [file 41598_2024_54835_MOESM4_ESM.zip › ╩2╛▌╝» - ╕▒▒╛/train/Negative/00115.jpg]

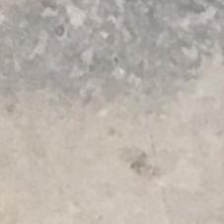

Supplement: Supplementary file 4 — Supplementary Information 4. [file 41598_2024_54835_MOESM4_ESM.zip › ╩2╛▌╝» - ╕▒▒╛/train/Negative/00116.jpg]

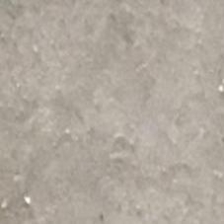

Supplement: Supplementary file 4 — Supplementary Information 4. [file 41598_2024_54835_MOESM4_ESM.zip › ╩2╛▌╝» - ╕▒▒╛/train/Negative/00117.jpg]

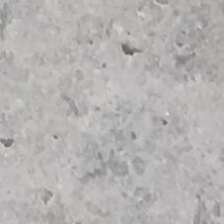

Supplement: Supplementary file 4 — Supplementary Information 4. [file 41598_2024_54835_MOESM4_ESM.zip › ╩2╛▌╝» - ╕▒▒╛/train/Negative/00118.jpg]

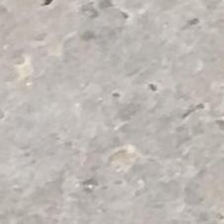

Supplement: Supplementary file 4 — Supplementary Information 4. [file 41598_2024_54835_MOESM4_ESM.zip › ╩2╛▌╝» - ╕▒▒╛/train/Negative/00119.jpg]

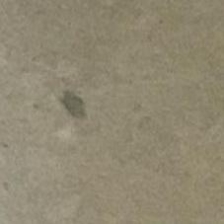

Supplement: Supplementary file 4 — Supplementary Information 4. [file 41598_2024_54835_MOESM4_ESM.zip › ╩2╛▌╝» - ╕▒▒╛/train/Negative/00120.jpg]

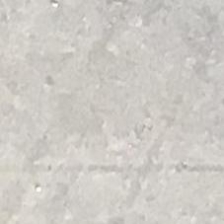

Supplement: Supplementary file 4 — Supplementary Information 4. [file 41598_2024_54835_MOESM4_ESM.zip › ╩2╛▌╝» - ╕▒▒╛/train/Negative/00121.jpg]

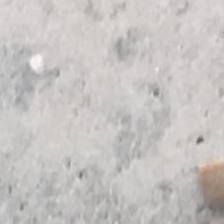

Supplement: Supplementary file 4 — Supplementary Information 4. [file 41598_2024_54835_MOESM4_ESM.zip › ╩2╛▌╝» - ╕▒▒╛/train/Negative/00122.jpg]

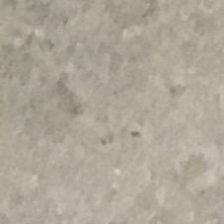

Supplement: Supplementary file 4 — Supplementary Information 4. [file 41598_2024_54835_MOESM4_ESM.zip › ╩2╛▌╝» - ╕▒▒╛/train/Negative/00123.jpg]

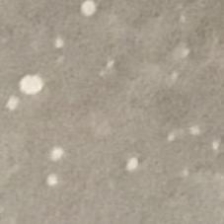

Supplement: Supplementary file 4 — Supplementary Information 4. [file 41598_2024_54835_MOESM4_ESM.zip › ╩2╛▌╝» - ╕▒▒╛/train/Negative/00124.jpg]

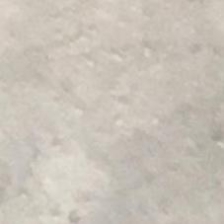

Supplement: Supplementary file 4 — Supplementary Information 4. [file 41598_2024_54835_MOESM4_ESM.zip › ╩2╛▌╝» - ╕▒▒╛/train/Negative/00125.jpg]

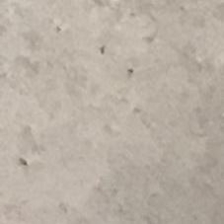

Supplement: Supplementary file 4 — Supplementary Information 4. [file 41598_2024_54835_MOESM4_ESM.zip › ╩2╛▌╝» - ╕▒▒╛/train/Negative/00126.jpg]

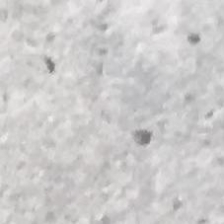

Supplement: Supplementary file 4 — Supplementary Information 4. [file 41598_2024_54835_MOESM4_ESM.zip › ╩2╛▌╝» - ╕▒▒╛/train/Negative/00127.jpg]

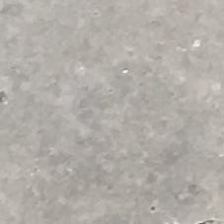

Supplement: Supplementary file 4 — Supplementary Information 4. [file 41598_2024_54835_MOESM4_ESM.zip › ╩2╛▌╝» - ╕▒▒╛/train/Negative/00128.jpg]

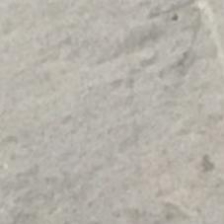

Supplement: Supplementary file 4 — Supplementary Information 4. [file 41598_2024_54835_MOESM4_ESM.zip › ╩2╛▌╝» - ╕▒▒╛/train/Negative/00129.jpg]

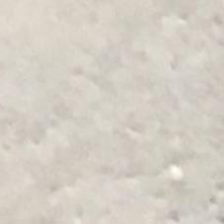

Supplement: Supplementary file 4 — Supplementary Information 4. [file 41598_2024_54835_MOESM4_ESM.zip › ╩2╛▌╝» - ╕▒▒╛/train/Negative/00130.jpg]

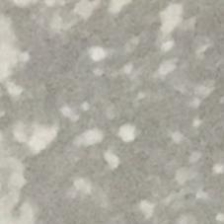

Supplement: Supplementary file 4 — Supplementary Information 4. [file 41598_2024_54835_MOESM4_ESM.zip › ╩2╛▌╝» - ╕▒▒╛/train/Negative/00131.jpg]

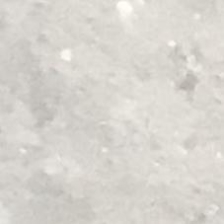

Supplement: Supplementary file 4 — Supplementary Information 4. [file 41598_2024_54835_MOESM4_ESM.zip › ╩2╛▌╝» - ╕▒▒╛/train/Negative/00132.jpg]

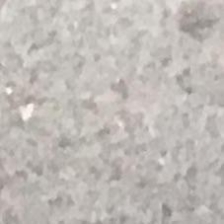

Supplement: Supplementary file 4 — Supplementary Information 4. [file 41598_2024_54835_MOESM4_ESM.zip › ╩2╛▌╝» - ╕▒▒╛/train/Negative/00133.jpg]

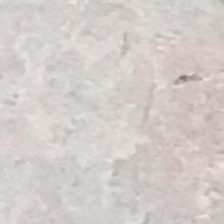

Supplement: Supplementary file 4 — Supplementary Information 4. [file 41598_2024_54835_MOESM4_ESM.zip › ╩2╛▌╝» - ╕▒▒╛/train/Negative/00134.jpg]

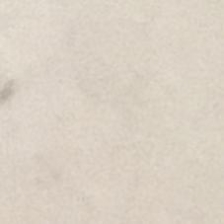

Supplement: Supplementary file 4 — Supplementary Information 4. [file 41598_2024_54835_MOESM4_ESM.zip › ╩2╛▌╝» - ╕▒▒╛/train/Negative/00135.jpg]

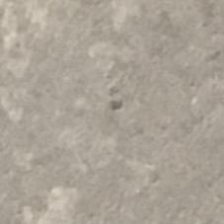

Supplement: Supplementary file 4 — Supplementary Information 4. [file 41598_2024_54835_MOESM4_ESM.zip › ╩2╛▌╝» - ╕▒▒╛/train/Negative/00136.jpg]

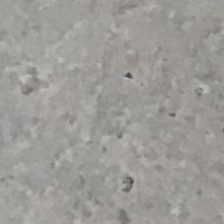

Supplement: Supplementary file 4 — Supplementary Information 4. [file 41598_2024_54835_MOESM4_ESM.zip › ╩2╛▌╝» - ╕▒▒╛/train/Negative/00137.jpg]

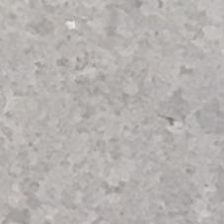

Supplement: Supplementary file 4 — Supplementary Information 4. [file 41598_2024_54835_MOESM4_ESM.zip › ╩2╛▌╝» - ╕▒▒╛/train/Negative/00138.jpg]

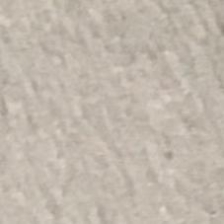

Supplement: Supplementary file 4 — Supplementary Information 4. [file 41598_2024_54835_MOESM4_ESM.zip › ╩2╛▌╝» - ╕▒▒╛/train/Negative/00139.jpg]

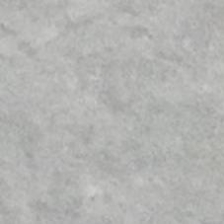

Supplement: Supplementary file 4 — Supplementary Information 4. [file 41598_2024_54835_MOESM4_ESM.zip › ╩2╛▌╝» - ╕▒▒╛/train/Negative/00140.jpg]

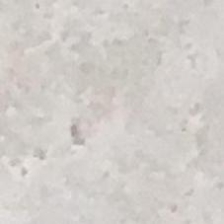

Supplement: Supplementary file 4 — Supplementary Information 4. [file 41598_2024_54835_MOESM4_ESM.zip › ╩2╛▌╝» - ╕▒▒╛/train/Negative/00141.jpg]

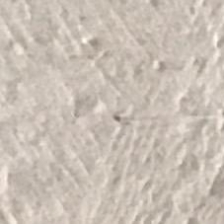

Supplement: Supplementary file 4 — Supplementary Information 4. [file 41598_2024_54835_MOESM4_ESM.zip › ╩2╛▌╝» - ╕▒▒╛/train/Negative/00142.jpg]

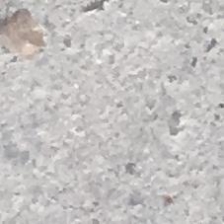

Supplement: Supplementary file 4 — Supplementary Information 4. [file 41598_2024_54835_MOESM4_ESM.zip › ╩2╛▌╝» - ╕▒▒╛/train/Negative/00143.jpg]

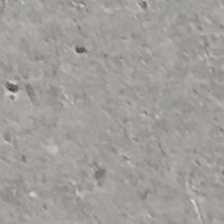

Supplement: Supplementary file 4 — Supplementary Information 4. [file 41598_2024_54835_MOESM4_ESM.zip › ╩2╛▌╝» - ╕▒▒╛/train/Negative/00144.jpg]

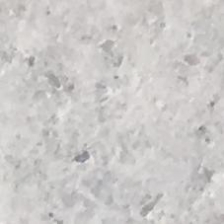

Supplement: Supplementary file 4 — Supplementary Information 4. [file 41598_2024_54835_MOESM4_ESM.zip › ╩2╛▌╝» - ╕▒▒╛/train/Negative/00145.jpg]

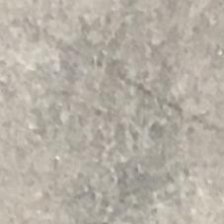

Supplement: Supplementary file 4 — Supplementary Information 4. [file 41598_2024_54835_MOESM4_ESM.zip › ╩2╛▌╝» - ╕▒▒╛/train/Negative/00146.jpg]

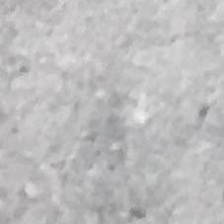

Supplement: Supplementary file 4 — Supplementary Information 4. [file 41598_2024_54835_MOESM4_ESM.zip › ╩2╛▌╝» - ╕▒▒╛/train/Negative/00147.jpg]

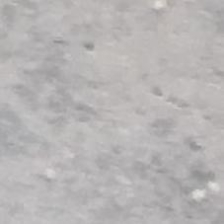

Supplement: Supplementary file 4 — Supplementary Information 4. [file 41598_2024_54835_MOESM4_ESM.zip › ╩2╛▌╝» - ╕▒▒╛/train/Negative/00148.jpg]

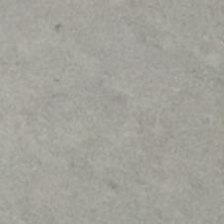

Supplement: Supplementary file 4 — Supplementary Information 4. [file 41598_2024_54835_MOESM4_ESM.zip › ╩2╛▌╝» - ╕▒▒╛/train/Negative/00149.jpg]

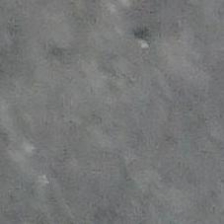

Supplement: Supplementary file 4 — Supplementary Information 4. [file 41598_2024_54835_MOESM4_ESM.zip › ╩2╛▌╝» - ╕▒▒╛/train/Negative/00150.jpg]

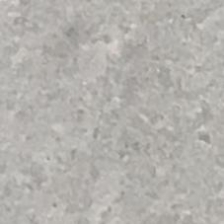

Supplement: Supplementary file 4 — Supplementary Information 4. [file 41598_2024_54835_MOESM4_ESM.zip › ╩2╛▌╝» - ╕▒▒╛/train/Negative/00151.jpg]

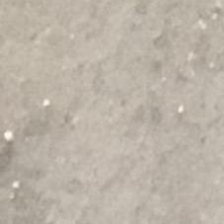

Supplement: Supplementary file 4 — Supplementary Information 4. [file 41598_2024_54835_MOESM4_ESM.zip › ╩2╛▌╝» - ╕▒▒╛/train/Negative/00152.jpg]

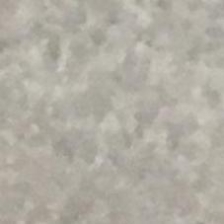

Supplement: Supplementary file 4 — Supplementary Information 4. [file 41598_2024_54835_MOESM4_ESM.zip › ╩2╛▌╝» - ╕▒▒╛/train/Negative/00153.jpg]

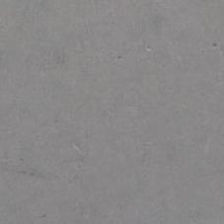

Supplement: Supplementary file 4 — Supplementary Information 4. [file 41598_2024_54835_MOESM4_ESM.zip › ╩2╛▌╝» - ╕▒▒╛/train/Negative/00154.jpg]

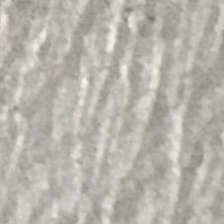

Supplement: Supplementary file 4 — Supplementary Information 4. [file 41598_2024_54835_MOESM4_ESM.zip › ╩2╛▌╝» - ╕▒▒╛/train/Negative/00155.jpg]

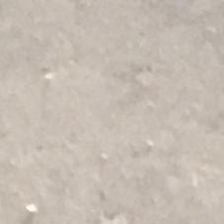

Supplement: Supplementary file 4 — Supplementary Information 4. [file 41598_2024_54835_MOESM4_ESM.zip › ╩2╛▌╝» - ╕▒▒╛/train/Negative/00156.jpg]

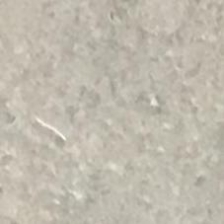

Supplement: Supplementary file 4 — Supplementary Information 4. [file 41598_2024_54835_MOESM4_ESM.zip › ╩2╛▌╝» - ╕▒▒╛/train/Negative/00157.jpg]

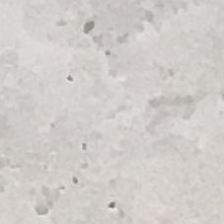

Supplement: Supplementary file 4 — Supplementary Information 4. [file 41598_2024_54835_MOESM4_ESM.zip › ╩2╛▌╝» - ╕▒▒╛/train/Negative/00158.jpg]

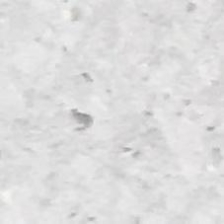

Supplement: Supplementary file 4 — Supplementary Information 4. [file 41598_2024_54835_MOESM4_ESM.zip › ╩2╛▌╝» - ╕▒▒╛/train/Negative/00159.jpg]

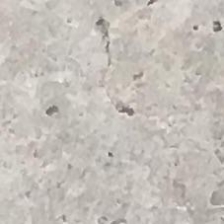

Supplement: Supplementary file 4 — Supplementary Information 4. [file 41598_2024_54835_MOESM4_ESM.zip › ╩2╛▌╝» - ╕▒▒╛/train/Negative/00160.jpg]

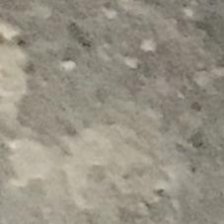

Supplement: Supplementary file 4 — Supplementary Information 4. [file 41598_2024_54835_MOESM4_ESM.zip › ╩2╛▌╝» - ╕▒▒╛/train/Negative/00161.jpg]

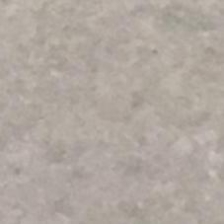

Supplement: Supplementary file 4 — Supplementary Information 4. [file 41598_2024_54835_MOESM4_ESM.zip › ╩2╛▌╝» - ╕▒▒╛/train/Negative/00162.jpg]

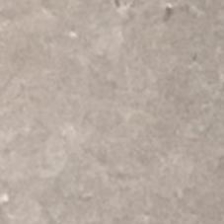

Supplement: Supplementary file 4 — Supplementary Information 4. [file 41598_2024_54835_MOESM4_ESM.zip › ╩2╛▌╝» - ╕▒▒╛/train/Negative/00163.jpg]

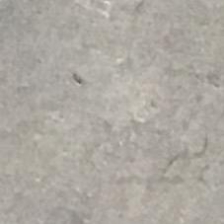

Supplement: Supplementary file 4 — Supplementary Information 4. [file 41598_2024_54835_MOESM4_ESM.zip › ╩2╛▌╝» - ╕▒▒╛/train/Negative/00164.jpg]

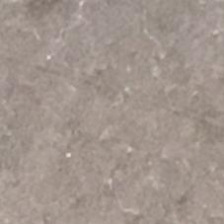

Supplement: Supplementary file 4 — Supplementary Information 4. [file 41598_2024_54835_MOESM4_ESM.zip › ╩2╛▌╝» - ╕▒▒╛/train/Negative/00165.jpg]

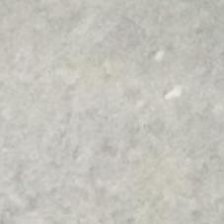

Supplement: Supplementary file 4 — Supplementary Information 4. [file 41598_2024_54835_MOESM4_ESM.zip › ╩2╛▌╝» - ╕▒▒╛/train/Negative/00166.jpg]

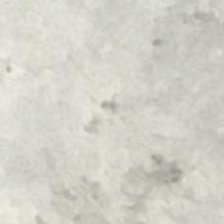

Supplement: Supplementary file 4 — Supplementary Information 4. [file 41598_2024_54835_MOESM4_ESM.zip › ╩2╛▌╝» - ╕▒▒╛/train/Negative/00167.jpg]

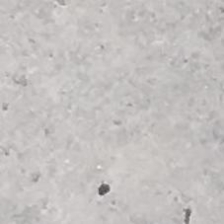

Supplement: Supplementary file 4 — Supplementary Information 4. [file 41598_2024_54835_MOESM4_ESM.zip › ╩2╛▌╝» - ╕▒▒╛/train/Negative/00168.jpg]

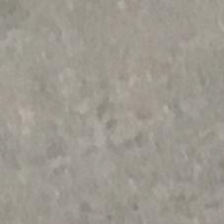

Supplement: Supplementary file 4 — Supplementary Information 4. [file 41598_2024_54835_MOESM4_ESM.zip › ╩2╛▌╝» - ╕▒▒╛/train/Negative/00169.jpg]

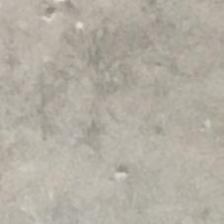

Supplement: Supplementary file 4 — Supplementary Information 4. [file 41598_2024_54835_MOESM4_ESM.zip › ╩2╛▌╝» - ╕▒▒╛/train/Negative/00170.jpg]

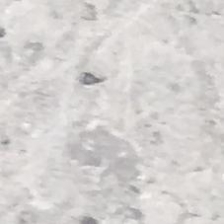

Supplement: Supplementary file 4 — Supplementary Information 4. [file 41598_2024_54835_MOESM4_ESM.zip › ╩2╛▌╝» - ╕▒▒╛/train/Negative/00171.jpg]

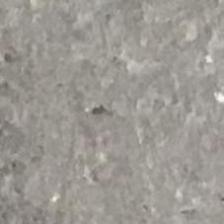

Supplement: Supplementary file 4 — Supplementary Information 4. [file 41598_2024_54835_MOESM4_ESM.zip › ╩2╛▌╝» - ╕▒▒╛/train/Negative/00172.jpg]

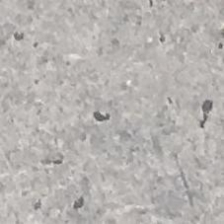

Supplement: Supplementary file 4 — Supplementary Information 4. [file 41598_2024_54835_MOESM4_ESM.zip › ╩2╛▌╝» - ╕▒▒╛/train/Negative/00173.jpg]

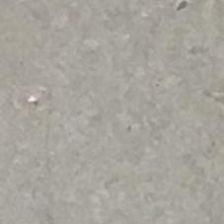

Supplement: Supplementary file 4 — Supplementary Information 4. [file 41598_2024_54835_MOESM4_ESM.zip › ╩2╛▌╝» - ╕▒▒╛/train/Negative/00174.jpg]

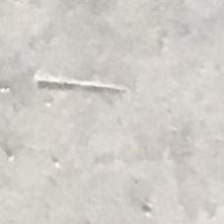

Supplement: Supplementary file 4 — Supplementary Information 4. [file 41598_2024_54835_MOESM4_ESM.zip › ╩2╛▌╝» - ╕▒▒╛/train/Negative/00175.jpg]

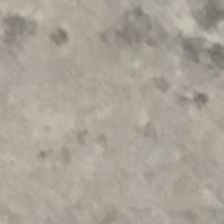

Supplement: Supplementary file 4 — Supplementary Information 4. [file 41598_2024_54835_MOESM4_ESM.zip › ╩2╛▌╝» - ╕▒▒╛/train/Negative/00176.jpg]

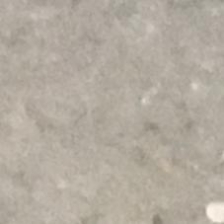

Supplement: Supplementary file 4 — Supplementary Information 4. [file 41598_2024_54835_MOESM4_ESM.zip › ╩2╛▌╝» - ╕▒▒╛/train/Negative/00177.jpg]

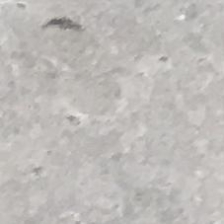

Supplement: Supplementary file 4 — Supplementary Information 4. [file 41598_2024_54835_MOESM4_ESM.zip › ╩2╛▌╝» - ╕▒▒╛/train/Negative/00178.jpg]

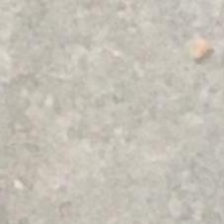

Supplement: Supplementary file 4 — Supplementary Information 4. [file 41598_2024_54835_MOESM4_ESM.zip › ╩2╛▌╝» - ╕▒▒╛/train/Negative/00179.jpg]

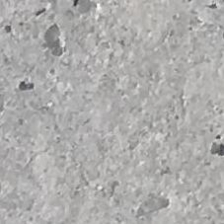

Supplement: Supplementary file 4 — Supplementary Information 4. [file 41598_2024_54835_MOESM4_ESM.zip › ╩2╛▌╝» - ╕▒▒╛/train/Negative/00180.jpg]

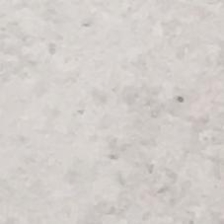

Supplement: Supplementary file 4 — Supplementary Information 4. [file 41598_2024_54835_MOESM4_ESM.zip › ╩2╛▌╝» - ╕▒▒╛/train/Negative/00181.jpg]

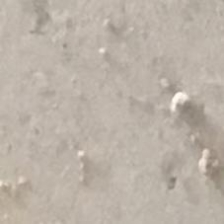

Supplement: Supplementary file 4 — Supplementary Information 4. [file 41598_2024_54835_MOESM4_ESM.zip › ╩2╛▌╝» - ╕▒▒╛/train/Negative/00182.jpg]

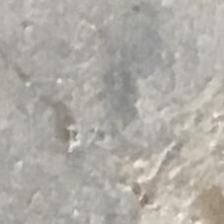

Supplement: Supplementary file 4 — Supplementary Information 4. [file 41598_2024_54835_MOESM4_ESM.zip › ╩2╛▌╝» - ╕▒▒╛/train/Negative/00183.jpg]

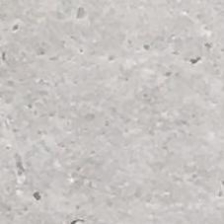

Supplement: Supplementary file 4 — Supplementary Information 4. [file 41598_2024_54835_MOESM4_ESM.zip › ╩2╛▌╝» - ╕▒▒╛/train/Negative/00184.jpg]

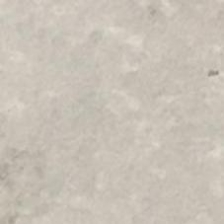

Supplement: Supplementary file 4 — Supplementary Information 4. [file 41598_2024_54835_MOESM4_ESM.zip › ╩2╛▌╝» - ╕▒▒╛/train/Negative/00185.jpg]

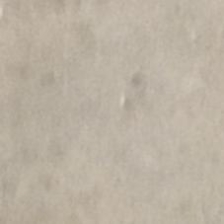

Supplement: Supplementary file 4 — Supplementary Information 4. [file 41598_2024_54835_MOESM4_ESM.zip › ╩2╛▌╝» - ╕▒▒╛/train/Negative/00186.jpg]

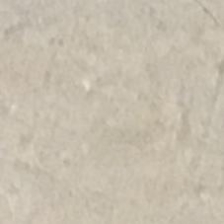

Supplement: Supplementary file 4 — Supplementary Information 4. [file 41598_2024_54835_MOESM4_ESM.zip › ╩2╛▌╝» - ╕▒▒╛/train/Negative/00187.jpg]

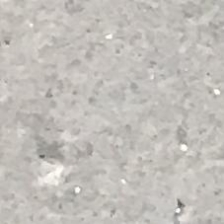

Supplement: Supplementary file 4 — Supplementary Information 4. [file 41598_2024_54835_MOESM4_ESM.zip › ╩2╛▌╝» - ╕▒▒╛/train/Negative/00188.jpg]

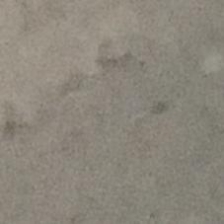

Supplement: Supplementary file 4 — Supplementary Information 4. [file 41598_2024_54835_MOESM4_ESM.zip › ╩2╛▌╝» - ╕▒▒╛/train/Negative/00189.jpg]

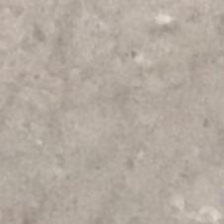

Supplement: Supplementary file 4 — Supplementary Information 4. [file 41598_2024_54835_MOESM4_ESM.zip › ╩2╛▌╝» - ╕▒▒╛/train/Negative/00190.jpg]

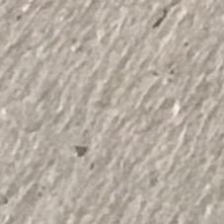

Supplement: Supplementary file 4 — Supplementary Information 4. [file 41598_2024_54835_MOESM4_ESM.zip › ╩2╛▌╝» - ╕▒▒╛/train/Negative/00191.jpg]

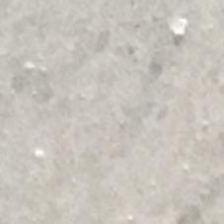

Supplement: Supplementary file 4 — Supplementary Information 4. [file 41598_2024_54835_MOESM4_ESM.zip › ╩2╛▌╝» - ╕▒▒╛/train/Negative/00192.jpg]

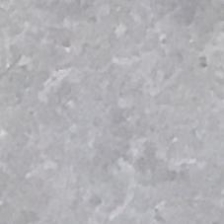

Supplement: Supplementary file 4 — Supplementary Information 4. [file 41598_2024_54835_MOESM4_ESM.zip › ╩2╛▌╝» - ╕▒▒╛/train/Negative/00193.jpg]

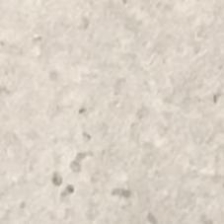

Supplement: Supplementary file 4 — Supplementary Information 4. [file 41598_2024_54835_MOESM4_ESM.zip › ╩2╛▌╝» - ╕▒▒╛/train/Negative/00194.jpg]

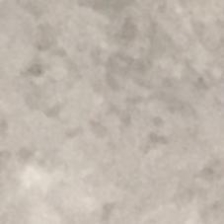

Supplement: Supplementary file 4 — Supplementary Information 4. [file 41598_2024_54835_MOESM4_ESM.zip › ╩2╛▌╝» - ╕▒▒╛/train/Negative/00195.jpg]

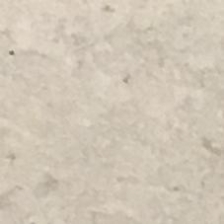

Supplement: Supplementary file 4 — Supplementary Information 4. [file 41598_2024_54835_MOESM4_ESM.zip › ╩2╛▌╝» - ╕▒▒╛/train/Negative/00196.jpg]

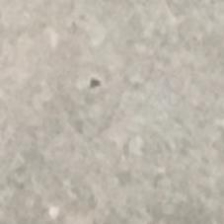

Supplement: Supplementary file 4 — Supplementary Information 4. [file 41598_2024_54835_MOESM4_ESM.zip › ╩2╛▌╝» - ╕▒▒╛/train/Negative/00197.jpg]

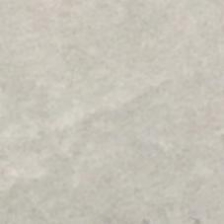

Supplement: Supplementary file 4 — Supplementary Information 4. [file 41598_2024_54835_MOESM4_ESM.zip › ╩2╛▌╝» - ╕▒▒╛/train/Negative/00198.jpg]

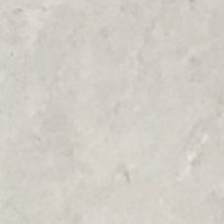

Supplement: Supplementary file 4 — Supplementary Information 4. [file 41598_2024_54835_MOESM4_ESM.zip › ╩2╛▌╝» - ╕▒▒╛/train/Negative/00199.jpg]

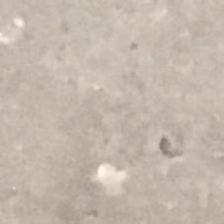

Supplement: Supplementary file 4 — Supplementary Information 4. [file 41598_2024_54835_MOESM4_ESM.zip › ╩2╛▌╝» - ╕▒▒╛/train/Negative/00200.jpg]
