# Supplementary figures and images for: An improved transformer-based concrete crack classification method (part 6 of 7)
Source: Sci Rep. 2024 Mar 14;14:6226. doi: 10.1038/s41598-024-54835-x (PMC10940720; doi:10.1038/s41598-024-54835-x)

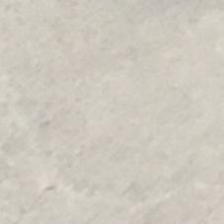

Supplement: Supplementary file 4 — Supplementary Information 4. [file 41598_2024_54835_MOESM4_ESM.zip › ╩2╛▌╝» - ╕▒▒╛/train/Negative/00011.jpg]

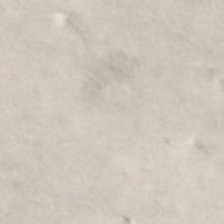

Supplement: Supplementary file 4 — Supplementary Information 4. [file 41598_2024_54835_MOESM4_ESM.zip › ╩2╛▌╝» - ╕▒▒╛/train/Negative/00012.jpg]

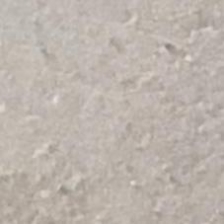

Supplement: Supplementary file 4 — Supplementary Information 4. [file 41598_2024_54835_MOESM4_ESM.zip › ╩2╛▌╝» - ╕▒▒╛/train/Negative/00013.jpg]

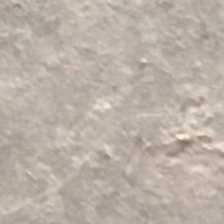

Supplement: Supplementary file 4 — Supplementary Information 4. [file 41598_2024_54835_MOESM4_ESM.zip › ╩2╛▌╝» - ╕▒▒╛/train/Negative/00014.jpg]

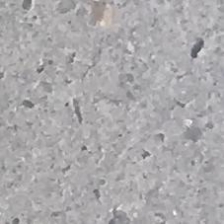

Supplement: Supplementary file 4 — Supplementary Information 4. [file 41598_2024_54835_MOESM4_ESM.zip › ╩2╛▌╝» - ╕▒▒╛/train/Negative/00015.jpg]

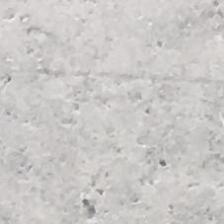

Supplement: Supplementary file 4 — Supplementary Information 4. [file 41598_2024_54835_MOESM4_ESM.zip › ╩2╛▌╝» - ╕▒▒╛/train/Negative/00016.jpg]

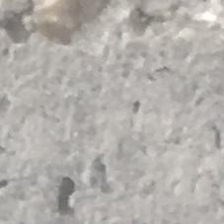

Supplement: Supplementary file 4 — Supplementary Information 4. [file 41598_2024_54835_MOESM4_ESM.zip › ╩2╛▌╝» - ╕▒▒╛/train/Negative/00017.jpg]

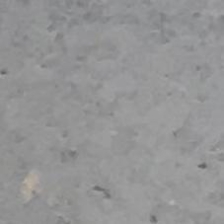

Supplement: Supplementary file 4 — Supplementary Information 4. [file 41598_2024_54835_MOESM4_ESM.zip › ╩2╛▌╝» - ╕▒▒╛/train/Negative/00018.jpg]

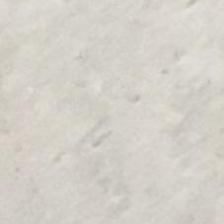

Supplement: Supplementary file 4 — Supplementary Information 4. [file 41598_2024_54835_MOESM4_ESM.zip › ╩2╛▌╝» - ╕▒▒╛/train/Negative/00019.jpg]

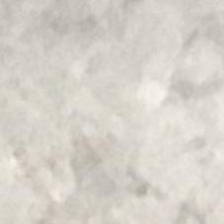

Supplement: Supplementary file 4 — Supplementary Information 4. [file 41598_2024_54835_MOESM4_ESM.zip › ╩2╛▌╝» - ╕▒▒╛/train/Negative/00020.jpg]

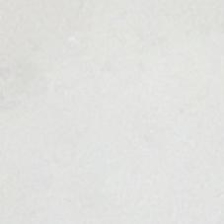

Supplement: Supplementary file 4 — Supplementary Information 4. [file 41598_2024_54835_MOESM4_ESM.zip › ╩2╛▌╝» - ╕▒▒╛/train/Negative/00021.jpg]

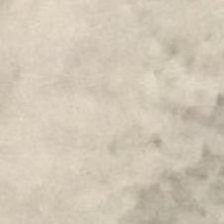

Supplement: Supplementary file 4 — Supplementary Information 4. [file 41598_2024_54835_MOESM4_ESM.zip › ╩2╛▌╝» - ╕▒▒╛/train/Negative/00022.jpg]

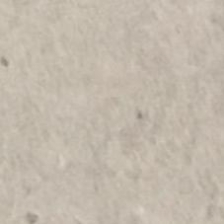

Supplement: Supplementary file 4 — Supplementary Information 4. [file 41598_2024_54835_MOESM4_ESM.zip › ╩2╛▌╝» - ╕▒▒╛/train/Negative/00023.jpg]

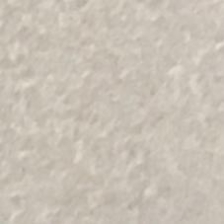

Supplement: Supplementary file 4 — Supplementary Information 4. [file 41598_2024_54835_MOESM4_ESM.zip › ╩2╛▌╝» - ╕▒▒╛/train/Negative/00024.jpg]

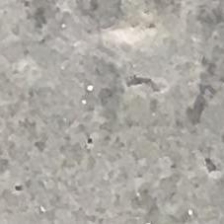

Supplement: Supplementary file 4 — Supplementary Information 4. [file 41598_2024_54835_MOESM4_ESM.zip › ╩2╛▌╝» - ╕▒▒╛/train/Negative/00025.jpg]

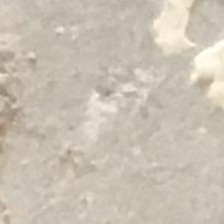

Supplement: Supplementary file 4 — Supplementary Information 4. [file 41598_2024_54835_MOESM4_ESM.zip › ╩2╛▌╝» - ╕▒▒╛/train/Negative/00026.jpg]

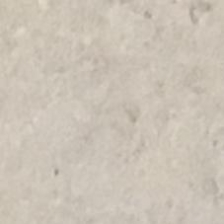

Supplement: Supplementary file 4 — Supplementary Information 4. [file 41598_2024_54835_MOESM4_ESM.zip › ╩2╛▌╝» - ╕▒▒╛/train/Negative/00027.jpg]

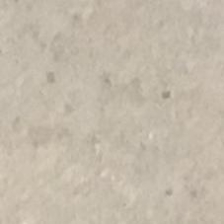

Supplement: Supplementary file 4 — Supplementary Information 4. [file 41598_2024_54835_MOESM4_ESM.zip › ╩2╛▌╝» - ╕▒▒╛/train/Negative/00028.jpg]

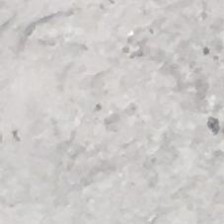

Supplement: Supplementary file 4 — Supplementary Information 4. [file 41598_2024_54835_MOESM4_ESM.zip › ╩2╛▌╝» - ╕▒▒╛/train/Negative/00029.jpg]

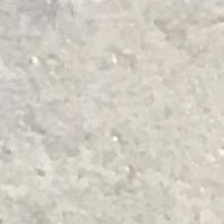

Supplement: Supplementary file 4 — Supplementary Information 4. [file 41598_2024_54835_MOESM4_ESM.zip › ╩2╛▌╝» - ╕▒▒╛/train/Negative/00030.jpg]

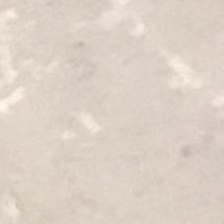

Supplement: Supplementary file 4 — Supplementary Information 4. [file 41598_2024_54835_MOESM4_ESM.zip › ╩2╛▌╝» - ╕▒▒╛/train/Negative/00031.jpg]

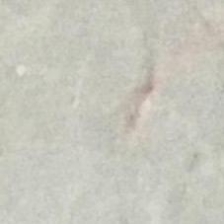

Supplement: Supplementary file 4 — Supplementary Information 4. [file 41598_2024_54835_MOESM4_ESM.zip › ╩2╛▌╝» - ╕▒▒╛/train/Negative/00032.jpg]

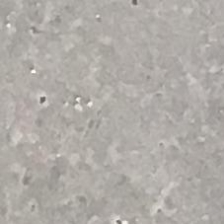

Supplement: Supplementary file 4 — Supplementary Information 4. [file 41598_2024_54835_MOESM4_ESM.zip › ╩2╛▌╝» - ╕▒▒╛/train/Negative/00033.jpg]

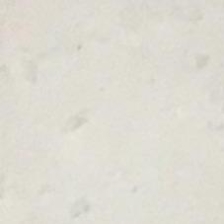

Supplement: Supplementary file 4 — Supplementary Information 4. [file 41598_2024_54835_MOESM4_ESM.zip › ╩2╛▌╝» - ╕▒▒╛/train/Negative/00034.jpg]

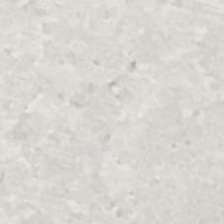

Supplement: Supplementary file 4 — Supplementary Information 4. [file 41598_2024_54835_MOESM4_ESM.zip › ╩2╛▌╝» - ╕▒▒╛/train/Negative/00035.jpg]

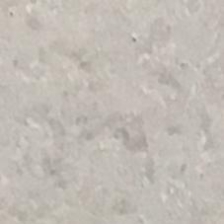

Supplement: Supplementary file 4 — Supplementary Information 4. [file 41598_2024_54835_MOESM4_ESM.zip › ╩2╛▌╝» - ╕▒▒╛/train/Negative/00036.jpg]

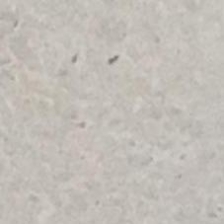

Supplement: Supplementary file 4 — Supplementary Information 4. [file 41598_2024_54835_MOESM4_ESM.zip › ╩2╛▌╝» - ╕▒▒╛/train/Negative/00037.jpg]

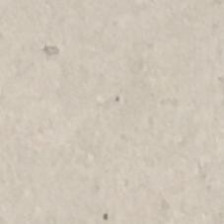

Supplement: Supplementary file 4 — Supplementary Information 4. [file 41598_2024_54835_MOESM4_ESM.zip › ╩2╛▌╝» - ╕▒▒╛/train/Negative/00038.jpg]

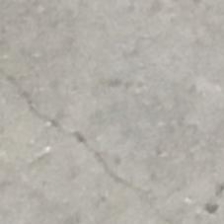

Supplement: Supplementary file 4 — Supplementary Information 4. [file 41598_2024_54835_MOESM4_ESM.zip › ╩2╛▌╝» - ╕▒▒╛/train/Negative/00039.jpg]

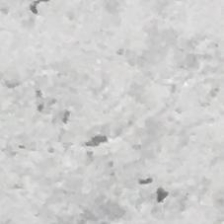

Supplement: Supplementary file 4 — Supplementary Information 4. [file 41598_2024_54835_MOESM4_ESM.zip › ╩2╛▌╝» - ╕▒▒╛/train/Negative/00040.jpg]

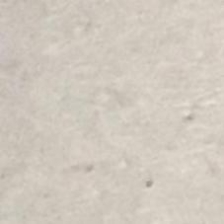

Supplement: Supplementary file 4 — Supplementary Information 4. [file 41598_2024_54835_MOESM4_ESM.zip › ╩2╛▌╝» - ╕▒▒╛/train/Negative/00041.jpg]

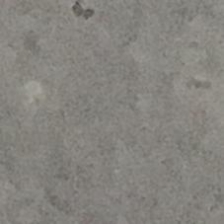

Supplement: Supplementary file 4 — Supplementary Information 4. [file 41598_2024_54835_MOESM4_ESM.zip › ╩2╛▌╝» - ╕▒▒╛/train/Negative/00042.jpg]

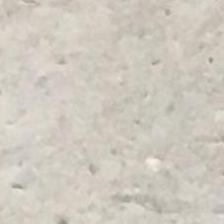

Supplement: Supplementary file 4 — Supplementary Information 4. [file 41598_2024_54835_MOESM4_ESM.zip › ╩2╛▌╝» - ╕▒▒╛/train/Negative/00043.jpg]

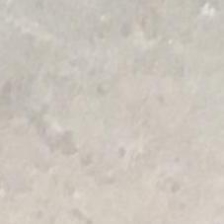

Supplement: Supplementary file 4 — Supplementary Information 4. [file 41598_2024_54835_MOESM4_ESM.zip › ╩2╛▌╝» - ╕▒▒╛/train/Negative/00044.jpg]

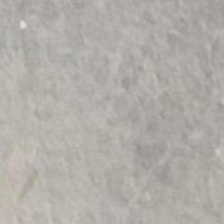

Supplement: Supplementary file 4 — Supplementary Information 4. [file 41598_2024_54835_MOESM4_ESM.zip › ╩2╛▌╝» - ╕▒▒╛/train/Negative/00045.jpg]

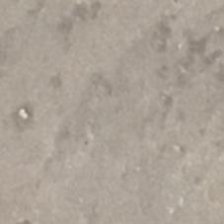

Supplement: Supplementary file 4 — Supplementary Information 4. [file 41598_2024_54835_MOESM4_ESM.zip › ╩2╛▌╝» - ╕▒▒╛/train/Negative/00046.jpg]

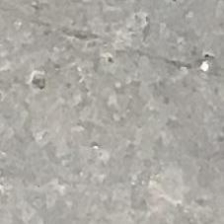

Supplement: Supplementary file 4 — Supplementary Information 4. [file 41598_2024_54835_MOESM4_ESM.zip › ╩2╛▌╝» - ╕▒▒╛/train/Negative/00047.jpg]

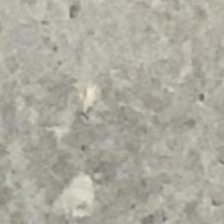

Supplement: Supplementary file 4 — Supplementary Information 4. [file 41598_2024_54835_MOESM4_ESM.zip › ╩2╛▌╝» - ╕▒▒╛/train/Negative/00048.jpg]

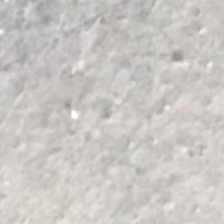

Supplement: Supplementary file 4 — Supplementary Information 4. [file 41598_2024_54835_MOESM4_ESM.zip › ╩2╛▌╝» - ╕▒▒╛/train/Negative/00049.jpg]

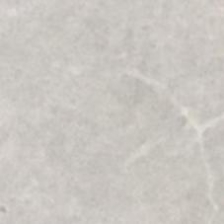

Supplement: Supplementary file 4 — Supplementary Information 4. [file 41598_2024_54835_MOESM4_ESM.zip › ╩2╛▌╝» - ╕▒▒╛/train/Negative/00050.jpg]

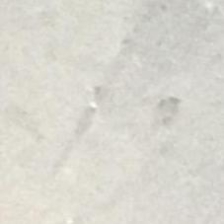

Supplement: Supplementary file 4 — Supplementary Information 4. [file 41598_2024_54835_MOESM4_ESM.zip › ╩2╛▌╝» - ╕▒▒╛/train/Negative/00051.jpg]

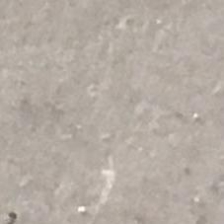

Supplement: Supplementary file 4 — Supplementary Information 4. [file 41598_2024_54835_MOESM4_ESM.zip › ╩2╛▌╝» - ╕▒▒╛/train/Negative/00052.jpg]

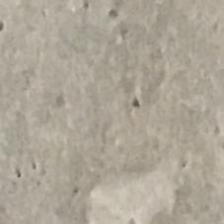

Supplement: Supplementary file 4 — Supplementary Information 4. [file 41598_2024_54835_MOESM4_ESM.zip › ╩2╛▌╝» - ╕▒▒╛/train/Negative/00053.jpg]

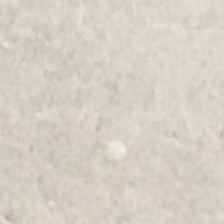

Supplement: Supplementary file 4 — Supplementary Information 4. [file 41598_2024_54835_MOESM4_ESM.zip › ╩2╛▌╝» - ╕▒▒╛/train/Negative/00054.jpg]

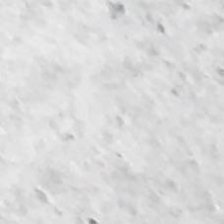

Supplement: Supplementary file 4 — Supplementary Information 4. [file 41598_2024_54835_MOESM4_ESM.zip › ╩2╛▌╝» - ╕▒▒╛/train/Negative/00055.jpg]

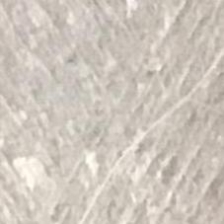

Supplement: Supplementary file 4 — Supplementary Information 4. [file 41598_2024_54835_MOESM4_ESM.zip › ╩2╛▌╝» - ╕▒▒╛/train/Negative/00056.jpg]

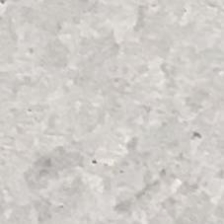

Supplement: Supplementary file 4 — Supplementary Information 4. [file 41598_2024_54835_MOESM4_ESM.zip › ╩2╛▌╝» - ╕▒▒╛/train/Negative/00057.jpg]

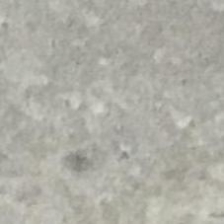

Supplement: Supplementary file 4 — Supplementary Information 4. [file 41598_2024_54835_MOESM4_ESM.zip › ╩2╛▌╝» - ╕▒▒╛/train/Negative/00058.jpg]

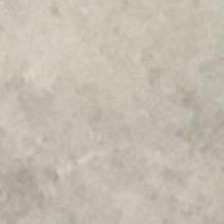

Supplement: Supplementary file 4 — Supplementary Information 4. [file 41598_2024_54835_MOESM4_ESM.zip › ╩2╛▌╝» - ╕▒▒╛/train/Negative/00059.jpg]

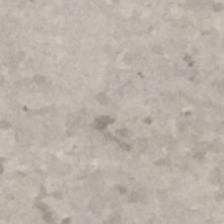

Supplement: Supplementary file 4 — Supplementary Information 4. [file 41598_2024_54835_MOESM4_ESM.zip › ╩2╛▌╝» - ╕▒▒╛/train/Negative/00060.jpg]

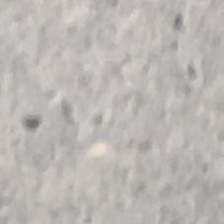

Supplement: Supplementary file 4 — Supplementary Information 4. [file 41598_2024_54835_MOESM4_ESM.zip › ╩2╛▌╝» - ╕▒▒╛/train/Negative/00061.jpg]

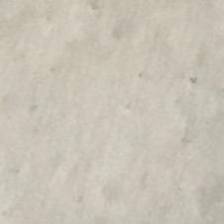

Supplement: Supplementary file 4 — Supplementary Information 4. [file 41598_2024_54835_MOESM4_ESM.zip › ╩2╛▌╝» - ╕▒▒╛/train/Negative/00062.jpg]

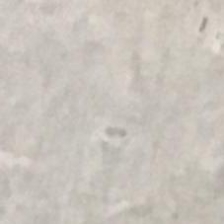

Supplement: Supplementary file 4 — Supplementary Information 4. [file 41598_2024_54835_MOESM4_ESM.zip › ╩2╛▌╝» - ╕▒▒╛/train/Negative/00063.jpg]

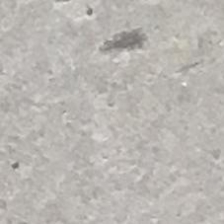

Supplement: Supplementary file 4 — Supplementary Information 4. [file 41598_2024_54835_MOESM4_ESM.zip › ╩2╛▌╝» - ╕▒▒╛/train/Negative/00064.jpg]

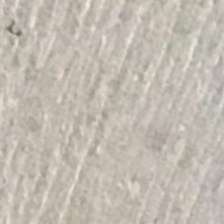

Supplement: Supplementary file 4 — Supplementary Information 4. [file 41598_2024_54835_MOESM4_ESM.zip › ╩2╛▌╝» - ╕▒▒╛/train/Negative/00065.jpg]

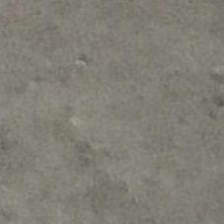

Supplement: Supplementary file 4 — Supplementary Information 4. [file 41598_2024_54835_MOESM4_ESM.zip › ╩2╛▌╝» - ╕▒▒╛/train/Negative/00066.jpg]

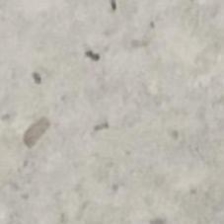

Supplement: Supplementary file 4 — Supplementary Information 4. [file 41598_2024_54835_MOESM4_ESM.zip › ╩2╛▌╝» - ╕▒▒╛/train/Negative/00067.jpg]

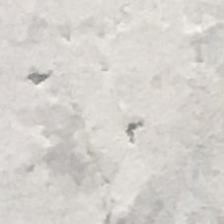

Supplement: Supplementary file 4 — Supplementary Information 4. [file 41598_2024_54835_MOESM4_ESM.zip › ╩2╛▌╝» - ╕▒▒╛/train/Negative/00068.jpg]

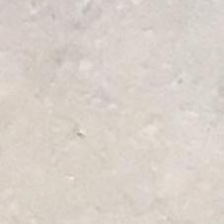

Supplement: Supplementary file 4 — Supplementary Information 4. [file 41598_2024_54835_MOESM4_ESM.zip › ╩2╛▌╝» - ╕▒▒╛/train/Negative/00069.jpg]

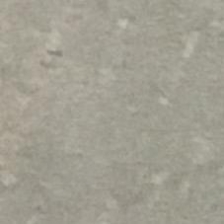

Supplement: Supplementary file 4 — Supplementary Information 4. [file 41598_2024_54835_MOESM4_ESM.zip › ╩2╛▌╝» - ╕▒▒╛/train/Negative/00070.jpg]

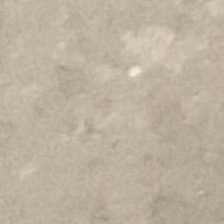

Supplement: Supplementary file 4 — Supplementary Information 4. [file 41598_2024_54835_MOESM4_ESM.zip › ╩2╛▌╝» - ╕▒▒╛/train/Negative/00071.jpg]

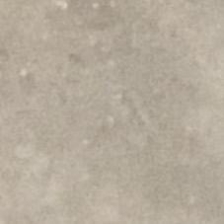

Supplement: Supplementary file 4 — Supplementary Information 4. [file 41598_2024_54835_MOESM4_ESM.zip › ╩2╛▌╝» - ╕▒▒╛/train/Negative/00072.jpg]

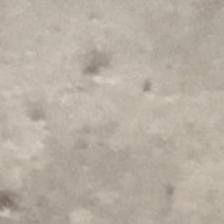

Supplement: Supplementary file 4 — Supplementary Information 4. [file 41598_2024_54835_MOESM4_ESM.zip › ╩2╛▌╝» - ╕▒▒╛/train/Negative/00073.jpg]

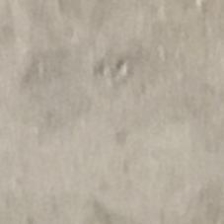

Supplement: Supplementary file 4 — Supplementary Information 4. [file 41598_2024_54835_MOESM4_ESM.zip › ╩2╛▌╝» - ╕▒▒╛/train/Negative/00074.jpg]

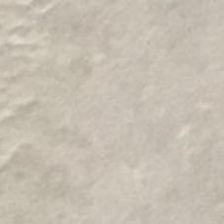

Supplement: Supplementary file 4 — Supplementary Information 4. [file 41598_2024_54835_MOESM4_ESM.zip › ╩2╛▌╝» - ╕▒▒╛/train/Negative/00075.jpg]

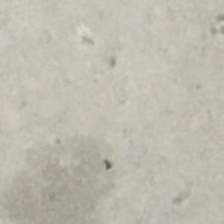

Supplement: Supplementary file 4 — Supplementary Information 4. [file 41598_2024_54835_MOESM4_ESM.zip › ╩2╛▌╝» - ╕▒▒╛/train/Negative/00076.jpg]

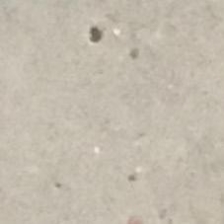

Supplement: Supplementary file 4 — Supplementary Information 4. [file 41598_2024_54835_MOESM4_ESM.zip › ╩2╛▌╝» - ╕▒▒╛/train/Negative/00077.jpg]

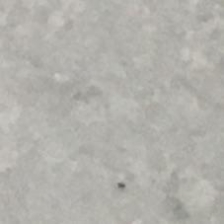

Supplement: Supplementary file 4 — Supplementary Information 4. [file 41598_2024_54835_MOESM4_ESM.zip › ╩2╛▌╝» - ╕▒▒╛/train/Negative/00078.jpg]

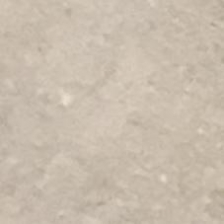

Supplement: Supplementary file 4 — Supplementary Information 4. [file 41598_2024_54835_MOESM4_ESM.zip › ╩2╛▌╝» - ╕▒▒╛/train/Negative/00079.jpg]

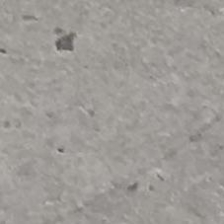

Supplement: Supplementary file 4 — Supplementary Information 4. [file 41598_2024_54835_MOESM4_ESM.zip › ╩2╛▌╝» - ╕▒▒╛/train/Negative/00080.jpg]

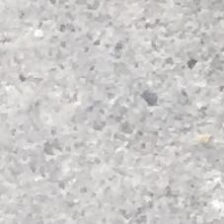

Supplement: Supplementary file 4 — Supplementary Information 4. [file 41598_2024_54835_MOESM4_ESM.zip › ╩2╛▌╝» - ╕▒▒╛/train/Negative/00081.jpg]

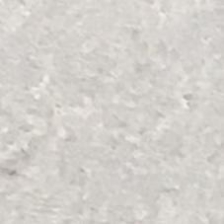

Supplement: Supplementary file 4 — Supplementary Information 4. [file 41598_2024_54835_MOESM4_ESM.zip › ╩2╛▌╝» - ╕▒▒╛/train/Negative/00082.jpg]

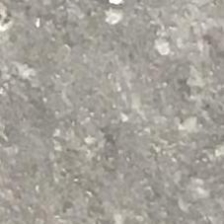

Supplement: Supplementary file 4 — Supplementary Information 4. [file 41598_2024_54835_MOESM4_ESM.zip › ╩2╛▌╝» - ╕▒▒╛/train/Negative/00083.jpg]

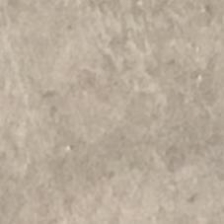

Supplement: Supplementary file 4 — Supplementary Information 4. [file 41598_2024_54835_MOESM4_ESM.zip › ╩2╛▌╝» - ╕▒▒╛/train/Negative/00084.jpg]

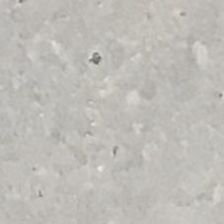

Supplement: Supplementary file 4 — Supplementary Information 4. [file 41598_2024_54835_MOESM4_ESM.zip › ╩2╛▌╝» - ╕▒▒╛/train/Negative/00085.jpg]

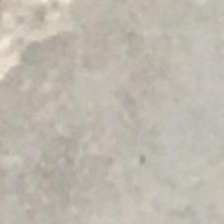

Supplement: Supplementary file 4 — Supplementary Information 4. [file 41598_2024_54835_MOESM4_ESM.zip › ╩2╛▌╝» - ╕▒▒╛/train/Negative/00086.jpg]

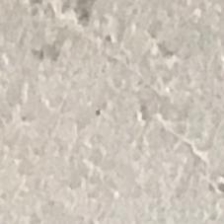

Supplement: Supplementary file 4 — Supplementary Information 4. [file 41598_2024_54835_MOESM4_ESM.zip › ╩2╛▌╝» - ╕▒▒╛/train/Negative/00087.jpg]

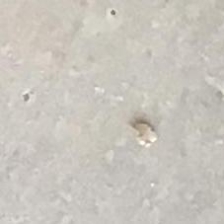

Supplement: Supplementary file 4 — Supplementary Information 4. [file 41598_2024_54835_MOESM4_ESM.zip › ╩2╛▌╝» - ╕▒▒╛/train/Negative/00088.jpg]

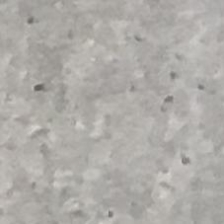

Supplement: Supplementary file 4 — Supplementary Information 4. [file 41598_2024_54835_MOESM4_ESM.zip › ╩2╛▌╝» - ╕▒▒╛/train/Negative/00089.jpg]

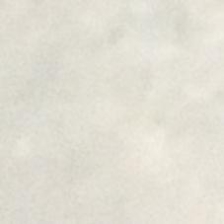

Supplement: Supplementary file 4 — Supplementary Information 4. [file 41598_2024_54835_MOESM4_ESM.zip › ╩2╛▌╝» - ╕▒▒╛/train/Negative/00090.jpg]

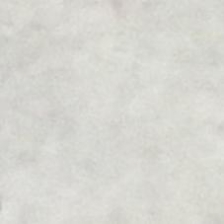

Supplement: Supplementary file 4 — Supplementary Information 4. [file 41598_2024_54835_MOESM4_ESM.zip › ╩2╛▌╝» - ╕▒▒╛/train/Negative/00091.jpg]

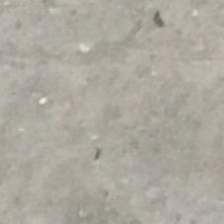

Supplement: Supplementary file 4 — Supplementary Information 4. [file 41598_2024_54835_MOESM4_ESM.zip › ╩2╛▌╝» - ╕▒▒╛/train/Negative/00092.jpg]

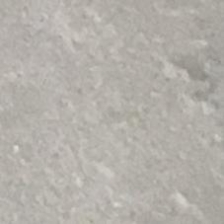

Supplement: Supplementary file 4 — Supplementary Information 4. [file 41598_2024_54835_MOESM4_ESM.zip › ╩2╛▌╝» - ╕▒▒╛/train/Negative/00093.jpg]

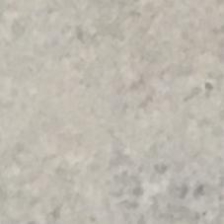

Supplement: Supplementary file 4 — Supplementary Information 4. [file 41598_2024_54835_MOESM4_ESM.zip › ╩2╛▌╝» - ╕▒▒╛/train/Negative/00094.jpg]

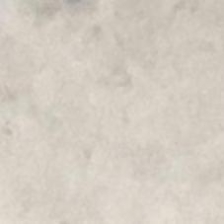

Supplement: Supplementary file 4 — Supplementary Information 4. [file 41598_2024_54835_MOESM4_ESM.zip › ╩2╛▌╝» - ╕▒▒╛/train/Negative/00095.jpg]

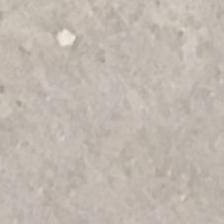

Supplement: Supplementary file 4 — Supplementary Information 4. [file 41598_2024_54835_MOESM4_ESM.zip › ╩2╛▌╝» - ╕▒▒╛/train/Negative/00096.jpg]

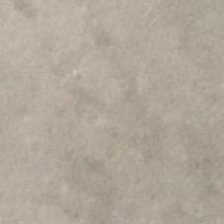

Supplement: Supplementary file 4 — Supplementary Information 4. [file 41598_2024_54835_MOESM4_ESM.zip › ╩2╛▌╝» - ╕▒▒╛/train/Negative/00097.jpg]

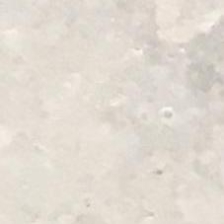

Supplement: Supplementary file 4 — Supplementary Information 4. [file 41598_2024_54835_MOESM4_ESM.zip › ╩2╛▌╝» - ╕▒▒╛/train/Negative/00098.jpg]

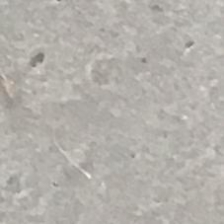

Supplement: Supplementary file 4 — Supplementary Information 4. [file 41598_2024_54835_MOESM4_ESM.zip › ╩2╛▌╝» - ╕▒▒╛/train/Negative/00099.jpg]

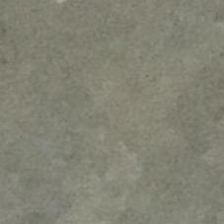

Supplement: Supplementary file 4 — Supplementary Information 4. [file 41598_2024_54835_MOESM4_ESM.zip › ╩2╛▌╝» - ╕▒▒╛/train/Negative/00100.jpg]

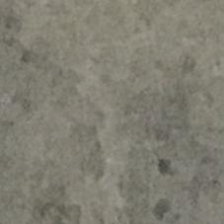

Supplement: Supplementary file 4 — Supplementary Information 4. [file 41598_2024_54835_MOESM4_ESM.zip › ╩2╛▌╝» - ╕▒▒╛/train/Negative/00101.jpg]

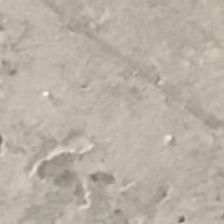

Supplement: Supplementary file 4 — Supplementary Information 4. [file 41598_2024_54835_MOESM4_ESM.zip › ╩2╛▌╝» - ╕▒▒╛/train/Negative/00102.jpg]

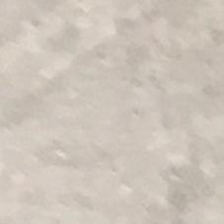

Supplement: Supplementary file 4 — Supplementary Information 4. [file 41598_2024_54835_MOESM4_ESM.zip › ╩2╛▌╝» - ╕▒▒╛/train/Negative/00103.jpg]

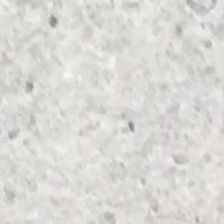

Supplement: Supplementary file 4 — Supplementary Information 4. [file 41598_2024_54835_MOESM4_ESM.zip › ╩2╛▌╝» - ╕▒▒╛/train/Negative/00104.jpg]

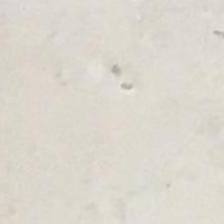

Supplement: Supplementary file 4 — Supplementary Information 4. [file 41598_2024_54835_MOESM4_ESM.zip › ╩2╛▌╝» - ╕▒▒╛/train/Negative/00105.jpg]

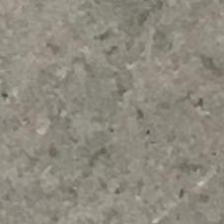

Supplement: Supplementary file 4 — Supplementary Information 4. [file 41598_2024_54835_MOESM4_ESM.zip › ╩2╛▌╝» - ╕▒▒╛/train/Negative/00106.jpg]

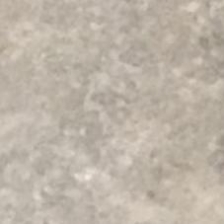

Supplement: Supplementary file 4 — Supplementary Information 4. [file 41598_2024_54835_MOESM4_ESM.zip › ╩2╛▌╝» - ╕▒▒╛/train/Negative/00107.jpg]

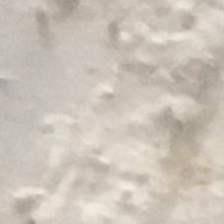

Supplement: Supplementary file 4 — Supplementary Information 4. [file 41598_2024_54835_MOESM4_ESM.zip › ╩2╛▌╝» - ╕▒▒╛/train/Negative/00108.jpg]

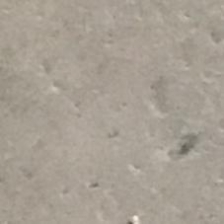

Supplement: Supplementary file 4 — Supplementary Information 4. [file 41598_2024_54835_MOESM4_ESM.zip › ╩2╛▌╝» - ╕▒▒╛/train/Negative/00109.jpg]

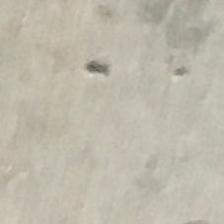

Supplement: Supplementary file 4 — Supplementary Information 4. [file 41598_2024_54835_MOESM4_ESM.zip › ╩2╛▌╝» - ╕▒▒╛/train/Negative/00110.jpg]
